# RNA‐binding protein ELAVL2 plays post‐transcriptional roles in the regulation of spermatogonia proliferation and apoptosis

**DOI:** 10.1111/cpr.13098

**Published:** 2021-07-23

**Authors:** Chao Yang, Chencheng Yao, Zhiyong Ji, Liangyu Zhao, Huixing Chen, Peng Li, Ruhui Tian, Erlei Zhi, Yuhua Huang, Xia Han, Yan Hong, Zhi Zhou, Zheng Li

**Affiliations:** ^1^ Department of Andrology The Center for Men's Health Urologic Medical Center Shanghai General Hospital Shanghai Jiao Tong University School of Medicine Shanghai China; ^2^ State Key Lab of Reproductive Medicine Nanjing Medical University Nanjing China; ^3^ School of Life Science and Technology ShanghaiTech University Shanghai China

**Keywords:** azoospermia, male infertility, post‐transcriptional regulation, RNA‐binding protein, spermatogonial stem cells

## Abstract

**Objectives:**

RNA‐binding proteins (RBPs) play essential post‐transcriptional roles in regulating spermatogonial stem cells (SSCs) maintenance and differentiation. We identified a conserved and SSCs‐enriched RBP ELAVL2 from our single‐cell sequencing data, but its function and mechanism in SSCs were unclear.

**Materials and methods:**

Expressions of ELAVL2 during human and mouse testis development were validated. Stable C18‐4 and TCam‐2 cell lines with overexpression and knockdown of ELAVL2 were established, which were applied to proliferation and apoptosis analysis. RNA immunoprecipitation and sequencing were used to identify ELAVL2 targets, and regulatory functions of ELAVL2 on target mRNAs were studied. Proteins interacting with ELAVL2 in human and mouse testes were identified using immunoprecipitation and mass spectrometric, which were validated by in vivo and in vitro experiments.

**Results:**

ELAVL2 was testis‐enriched and preferentially expressed in human and mouse SSCs. ELAVL2 was down‐regulated in NOA patients. ELAVL2 promoted proliferation and inhibited apoptosis of C18‐4 and TCam‐2 cell lines via activating ERK and AKT pathways. ELAVL2 associated with mRNAs encoding essential regulators of SSCs proliferation and survival, and promoted their protein expression at post‐transcriptional level. ELAVL2 interacted with DAZL in vivo and in vitro in both human and mouse testes.

**Conclusions:**

Taken together, these results indicate that ELAVL2 is a conserved SSCs‐enriched RBP that down‐regulated in NOA, which regulates spermatogonia proliferation and apoptosis by promoting protein expression of targets.

## Graphical abstract

ELAVL2 is enriched in human SSCs and downregulated in NOA SSCs. ELAVL2 is essential for spermatogonia proliferation and survival, which promotes protein expression of target genes.

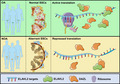

## 1 INTRODUCTION

Male infertility is a common reproductive disorder which contributes to about 10% ~ 15% of infertile couples in the world.^1^ Azoospermia, consisted of obstructive azoospermia (OA) and non‐obstructive azoospermia (NOA), is the severest cause of male infertility. OA patients have normal spermatogenesis, and usually could have their biological offspring with the assistance of microsurgery and assisted reproductive technology. While NOA usually results from testicular failure and aberrant spermatogenesis caused by endocrine diseases, genetic disorders, environmental and medical factors, or other unknown reasons, and the treatment for NOA is still limited and poor.^2^, ^3^ The key to solving this problem is exploring and revealing the mechanisms of human spermatogenesis.

Spermatogenesis comprises the self‐renewal and differentiation of spermatogonial stem cells (SSCs), meiosis of spermatocytes and spermiogenesis, which is an intricate multi‐phase process of continuous proliferation, differentiation and apoptosis of germ cells.^4^, ^5^ SSCs are unique since they are the only stem cells in the body that undergo self‐renewal throughout life and transmit genetic information to offspring. The maintenance of SSCs and thus normal spermatogenesis are meticulously regulated by intrinsic gene expression within the stem cells and the stem cell niche formed by Sertoli cells, the basement membrane, and blood vessels, which provide the growth factors and the extracellular signals for SSCs.^6^, ^7^ It has been demonstrated that glial cell line‐derived neurotrophic factor (GDNF), GDNF‐family receptor α‐1 (GFRα1) and fibroblast growth factor 2 (FGF2) are essential for replication and expansion of mouse SSCs, while stem cell factor (SCF), c‐KIT and retinoic acid (RA) are crucial for SSCs differentiation.^8^, ^9^, ^10^

In SSCs, the expression of genes that mediate stem cells fate determination is under elaborate multi‐layer regulation, including post‐transcriptional regulation, which has been extensively studied during meiosis and spermiogenesis.^11^, ^12^ During homologous recombination of spermatocytes entering early meiosis, the genome is damaged and transcription is blocked.^13^ During the late steps of spermiogenesis, the chromosomes are highly compacted through the substitution of histones with transition proteins and protamines, leading to mass degradation of mRNAs and the gradual decline of translation.^14^ Consequently, the mechanisms of post‐transcriptional regulation, controlled primarily by RNA binding proteins (RBPs), become of utmost importance. Recent researches also revealed the essential role of RBPs‐mediated post‐transcriptional regulation in mouse SSCs self‐renewal, proliferation and differentiation, although transcription is active in SSCs.

RNA‐binding proteins are an extensive class of proteins defined by their ability to recognize and bind to specific sequences of RNA through RNA‐binding domains (RBDs).^15^ RBPs control the whole course of RNA metabolism, from pre‑mRNA splicing to mRNA export, mRNA stability, degradation and translation. RBPs form dynamic interactions with coding, untranslated and non–protein‐coding RNAs in functional units called ribonucleoproteins (RNP) complexes. This enables the RBPs within RNP complexes to remain stably bound to the RNA throughout its journey from synthesis to degradation or to associate with the RNAs in a temporal and spatial manner.^16^ RBPs regulate nearly all aspects of cell fate, like proliferation, apoptosis, stress adaptation, stem cells self‐renewal and differentiation. Genetic and proteomic data and evidence from animal models reveal that aberrant RBP functions are involved in many human diseases including neurologic disorders, cancer and male infertility.^17^, ^18^ Nanos2 is predominantly expressed in gonocytes and SSCs, and the elimination of this RBP results in a complete loss of spermatogonia.^19^ Nanos2 works with other cellular messenger ribonucleoproteins (mRNPs) components to ensure the primitive status of SSCs through a dual mechanism, including direct recruitment and translational repression of genes that promote SSCs differentiation, and repression of the target of rapamycin complex 1 (mTORC1), a well‐known negative pathway for SSC self‐renewal, by sequestration of the core factor mTOR in mRNPs.^20^ Nanos3 is expressed in migrating gonocytes and SSCs after birth, with deletion of this factor resulting in a reduction in germ cell numbers and decreased expression of germ cell‐intrinsic genes required for the maintenance of pluripotency and meiotic initiation and progression.^21^, ^22^ DND1 binds a UU(A/U) trinucleotide motif predominantly in the 3′‐UTR of mRNA, and destabilizes target mRNAs through direct recruitment of the CCR4–NOT deadenylase complex. The spectrum of target RNAs includes positive regulators of apoptosis and inflammation, and modulators of signalling pathways that regulate stem‐cell pluripotency, including the TGF‐β superfamily. This DND1‐dependent mRNA destabilization is required for the survival of mouse PGCs and maintenance of SSCs.^23^, ^24^ These researches proved the significance of RBPs in SSCs, however, they were mainly studied in murine SSCs. Our knowledge about the roles of RBPs in human SSCs and male infertility is still limited.

Previously, our team conducted single‐cell sequence with testicular tissues from OA and NOA patients. Distinct stages of spermatogonia were identified, including three stages of SSCs, differentiating and differentiated spermatogonia.^25^ By comparing the transcriptome of spermatogonia between OA and NOA patients, we focused on several testis‐enriched RBPs, which were abnormally expressed and considered potentially pathogenic. Among these RBPs, ELAVL2 is the most down‐regulated in NOA, and its expression is enriched in human spermatogonia. ELAVL2 (also known as HuB and HEL‐N1) is a member of the ELAVL RNA‐binding proteins, which promotes translation by associating with mRNAs containing AU‐rich elements.^26^, ^27^ ELAVL2 preferentially expresses in human and mouse brains and testes. Another study also showed the expression of ELAVL2 in adipose tissues, adipocytes and preadipocytes.^28^ Strikingly, Wu X et al demonstrated that ELAVL2 was conserved and highly enriched in both human spermatogonia and mouse gonocytes, and ELAVL2 protein was localized to the nucleus in human spermatogonia.^29^ The results were consistent with ours, indicating a conserved role of ELAVL2 in human and mouse spermatogonia. ELAVL2‐regulated transcriptional and splicing networks in human neurons are critical for neuronal function and clinically relevant to autism spectrum disorder.^26^, ^27^ In mouse ovary, ELAVL2 associates with mRNAs encoding components of P‐bodies (cytoplasmic RNP granules involved in the decay and storage of RNA) and directs the assembly of P‐body‐like granules by promoting the translation of DDX6 in oocyte, which is essential to the formation of primordial follicles.^30^ However, the functions of ELAVL2 in testis remain unclear.

In this study, we focused on ELAVL2 according to our previous single‐cell sequence results and explored the expression pattern and functions of ELAVL2 in testis, especially in SSCs. We found that ELAVL2 is expressed in human and mouse spermatogonia and is enriched in SSCs. In vitro studies revealed that ELAVL2 promotes spermatogonia proliferation and inhibits apoptosis. Target mRNAs of ELAVL2 revealed by RIP‐seq were enriched in KEGG and GO terms associated with proliferation and stem cell self‐renewal and pluripotency regulation. ELAVL2 does not affect target mRNA expression, but significantly promotes their translation, including several indispensable genes for SSCs maintenance, like *Id4*, *Sall4*, *Etv5*, etc ELAVL2 interacts with DAZL in both human and mouse SSCs, a well‐recognized RBP that regulate SSCs maintenance and survival. Collectively, this study could offer new post‐transcriptional mechanisms about the regulation of spermatogenesis, as well as novel pathogenesis of male infertility, and provide new targets for potential therapy in the future.

## 2 MATERIALS AND METHODS

### 2.1 Ethics statement

The work was approved by the Institutional Review Board of Shanghai General Hospital (license number: 2018KY052).

### 2.2 Procurement of testicular biopsies from patients

Written informed consents for testicular biopsies were obtained from the patients for the research only, including adult patients and the legal guardian of children. Fresh testicular tissues were obtained from 4 children who underwent testicular biopsies or partial excision for the following indications: testicular torsion (n = 1, 2‐years) and testicular tumours (n = 3, 5‐years, 11‐years, and 17‐years). The 2‐year‐old child presented with scrotum swelling and redness, as well as symptoms of pain including crying and vomiting for about 30 hours. Physical examination and ultrasonography all indicated right testicular torsion. Emergency surgery was conducted, which found that right testicle twisted clockwise for about 270° and the colour was dark purple. Also, the left testicle twisted clockwise for about 90°, but its colour was mostly normal, which may result from congenital development abnormality as we speculated. After detorsion for about 15 minutes, the colour of right testicle did not turn normal, then both testicles were cut open to observe the condition of testicular tissues, which is a standard surgery procedure. A buck of seminiferous tubules of right testicle was deep dark in colour, which were considered as necrotic tissues. The assumed necrotic tissues and adjacent tissues were completely excised for pathological examination. A small part of left testicular tissue in the deep of incision that presented a little dark in colour was also cut for pathological examination in consideration of safety. Both testicles were then fixed in the scrotum. Postoperative pathological examination showed necrosis in the right testicular tissue, while the adjacent tissue and left testicular tissue only showed a little infiltration of inflammatory cells, and the seminiferous tubules were mostly intact. As for the other three children with testicular tumours, their symptoms and treatment were similar. They all presented with testicular mass during physical examination, and imageological examination all indicated testicular tumours with relatively clear outlines. In order to reduce impairment and preserve their fertility as much as possible, testicular tumour resection under microscope was conducted, which helped to better distinguish the outlines of tumours. The tumours and adjacent tissues were resected for pathological examination according to standard surgery principles, which were proved to be benign. The remaining testicular tissues after pathological examination were collected for the research, which had got informed consent from the legal guardian of the children. The original informed consents for the surgery and research were attached in Appendix S3. An additional 5 OA and 3 NOA samples were obtained from the remaining tissues after testicular sperm extraction operation. All children had normal karyotypes, genotypes and sex hormone levels. Patients with OA were caused by inflammation and vasoligation but not by congenital absence of the vas deferens (CBAVD) or other diseases. Patients with NOA were confirmed by surgery and histological analysis, and patients with reproductive congenital diseases such as genomic AZF deletions and loss‐of‐function gene mutations, or other diseases including cancer were excluded in this study.

### 2.3 Single‐cell RNA sequencing (scRNA‐seq) analysis

The detailed analysis was described previously.^25^ In brief, single testicular cells were isolated from both human and mouse testis by enzyme digestion, which were loaded on a Chromium Single Cell Controller instrument (10× Genomics) to generate single‐cell gel beads in emulsions. The scRNA‐seq libraries were prepared using the Chromium Single Cell 3′ Library & Gel Bead Kit. After mapping, sample quality control, and integration, 16 clusters of all testicular cells were identified. Unique cluster‐specific marker genes were identified using the Seurat FindAllMarkers tool, which is based on the normalized UMI count.

### 2.4 Cell culture

The C18‐4 cells were cultured with DMEM/F12 medium containing 10% FBS and 2 mM glutamine. TCam‐2 cells were cultured in high‐glucose DMEM media supplemented with 10% FBS at 37°C and 5% CO_2_ incubation. Primary GS cell lines were established and cultured as previously described.^31^ For inhibition of AKT and ERK signalling pathways, AKT inhibitor GSK690693 and ERK inhibitor SCH772984 were added to C18‐4 cells at a concentration of 150 nM and 500 nM for 24 hours, respectively.

### 2.5 Supplementary methods

Additional methods are shown in Supplementary Material (Appendix S1).

## 3 RESULTS

### 3.1 ELAVL2 is enriched in SSCs and down‐regulated in NOA

**FIGURE 1:**
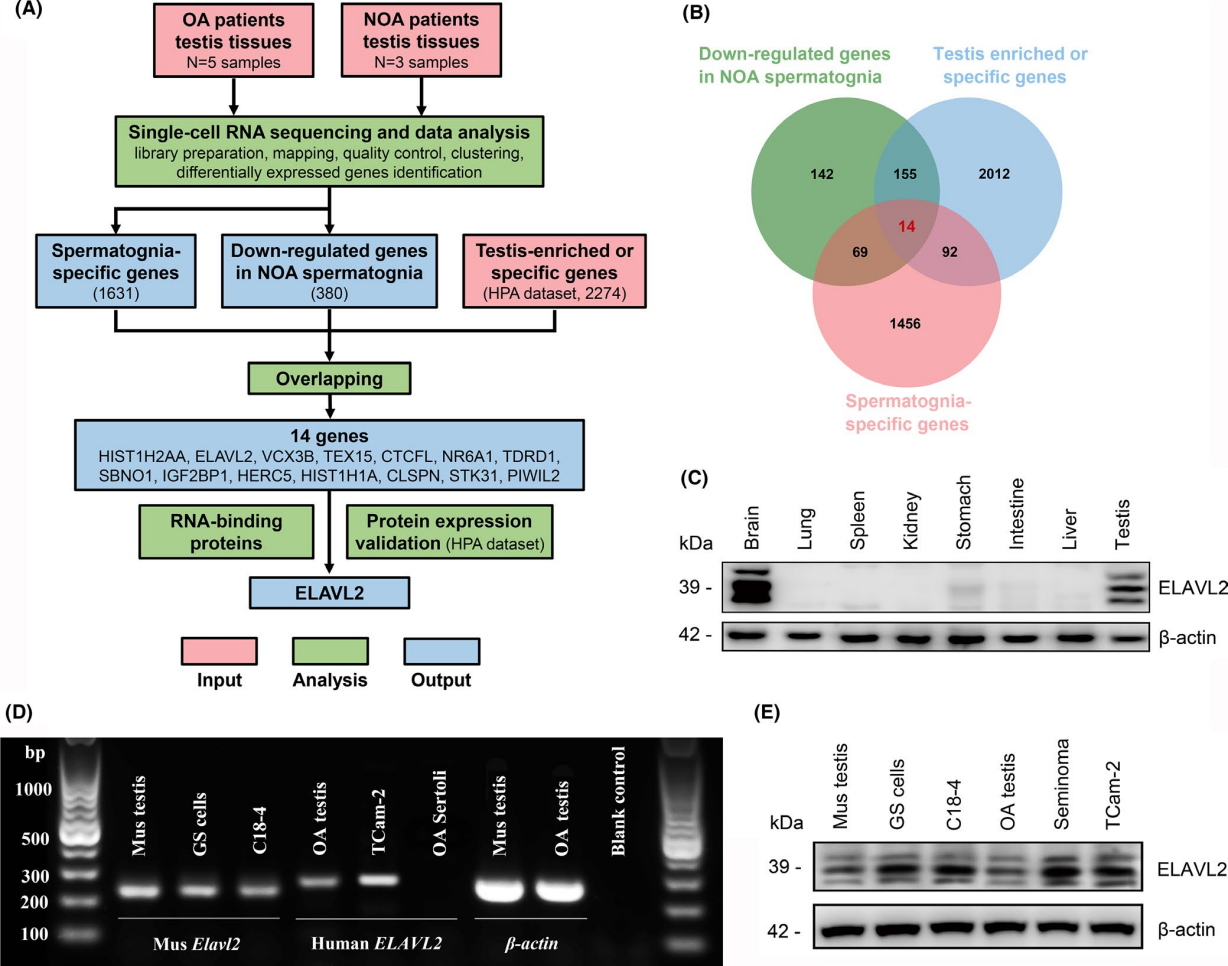
Clinical relevance of ELAVL2 to NOA and its expression in human and mouse tissues and cells. (A) Analysis pipeline for discovery of RNA‐binding proteins that potentially associated with NOA based on comparison of scRNA‐seq results between OA and NOA patients. (B) Venn diagram of all differentially down‐regulated genes in NOA spermatogonia. (C) Protein expression of ELAVL2 in various mouse tissues detected by Western blot. (D, E) mRNA and protein expression of ELAVL2 in human and mouse testis tissues, seminoma tissues, primary GS cells, C18‐4 and TCam‐2 cell lines. Sertoli cell served as negative control

**FIGURE 2:**
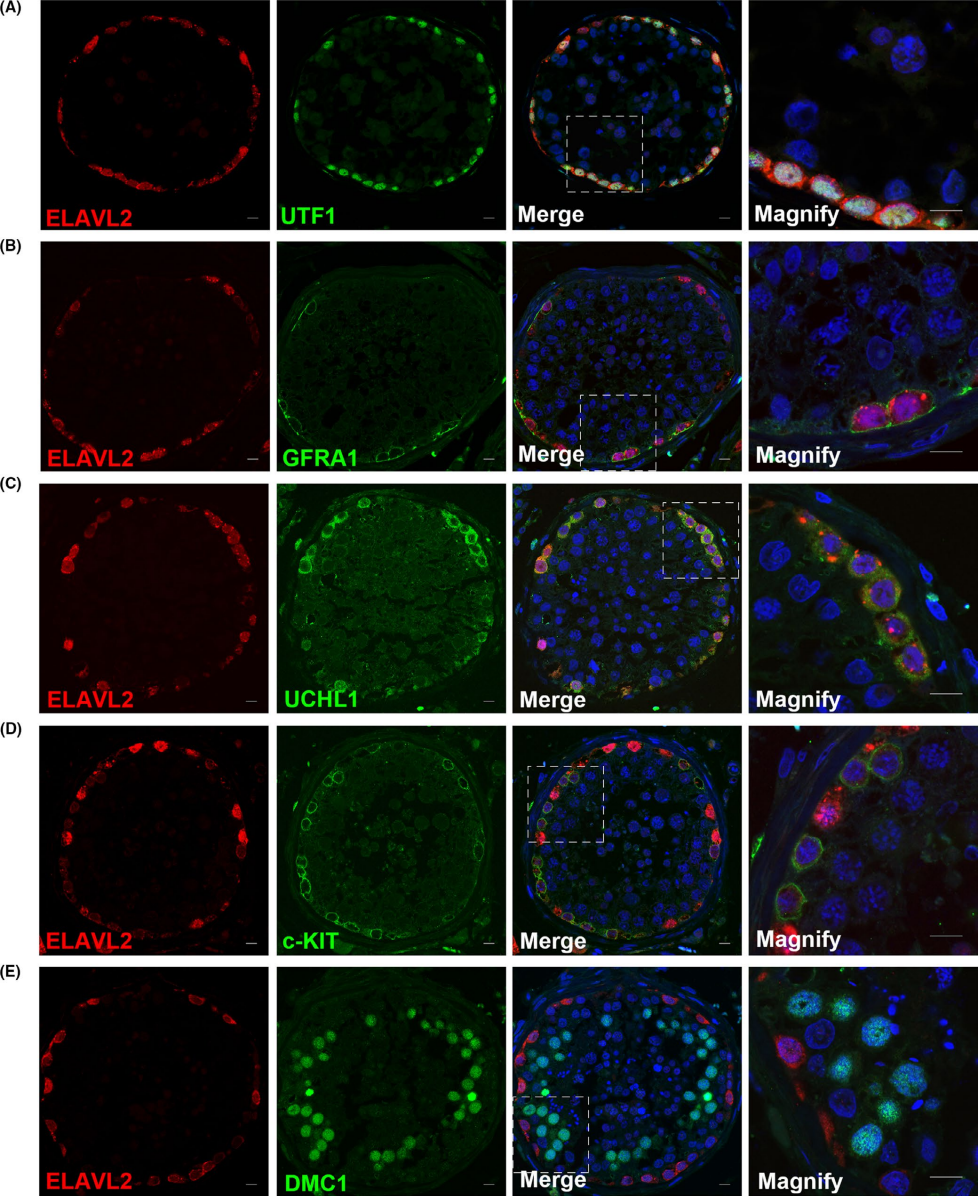
ELAVL2 is enriched in human spermatogonial stem cells (SSCs). (A, B, C) Co‐staining of ELAVL2 with human SSCs markers UTF1 (A), GFRA1 (B) and spermatogonia marker UCHL1 (C). Scale bar: 20 µm. (D) Co‐staining of ELAVL2 with human differentiating spermatogonia marker c‐KIT. Scale bar: 20 µm. (E) Co‐staining of ELAVL2 with spermatocyte marker DMC1. Scale bar: 20 µm. Cell nuclei were stained with DAPI

To identify consistently altered RBPs in spermatogonia (SPG) of NOA patients, we analysed our single‐cell transcriptome data of OA and NOA testis tissues. After quality control, clustering, and differentially expressed genes identification (log_2_ [fold change] > 1, *P* < .05), we focused on spermatogonia‐specific genes (n = 1631, Table S1) and down‐regulated genes in NOA spermatogonia (n = 380, Figure S1A, Table S2). By overlapping these two gene sets with testis‐enriched or specific genes from HPA dataset (The Human Protein Atlas, n = 2274, Table S3), we screened out fourteen genes, and only four of them were RBPs (Figure 1A,B). Ultimately, we focused on ELAVL2 for two reasons: (1) HPA dataset shows that both ELAVL2 mRNA and protein are enriched in human testis and brain, and our Western blot also revealed the preferential expression of ELAVL2 in mouse testis and brain (Figure 1C); (2) ELAVL2 is highly conserved from rodent animals to primates, especially the three functional RNA recognition motifs (RRMs) (Figure S1B).

Single‐cell analysis revealed that ELAVL2 mRNA is enriched in spermatogonia, while the other three members of ELAVL family do not show this expression pattern. Strikingly, the ELAVL2 transcript is highly expressed in human spermatogonia, and is significantly down‐regulated after differentiation (Figure S1C). ELAVL2 mRNA and protein were also detected in human and mouse testis tissues, primary cultured mouse SSCs (Germ line stem cells, GS cells) and mouse SSCs cell line C18‐4 (Figure 1D,E). We also found that ELAVL2 is highly expressed in seminoma tissues and TCam‐2, a well‐recognized seminoma cell line (Figure 1D,E and Figure S2). In phenotype, TCam‐2 resembles progenitor cells of male germline and expresses several gonocytes and SSCs markers, including SALL4, POU5F1, and NANOG.^32^, ^33^ Both C18‐4 and TCcm‐2 were employed as in vitro models for further functional studies.

### 3.2 ELAVL2 stage‐specific expression during testis development and spermatogenesis

To better understand the functions of ELAVL2, ELAVL2 protein enrichment during testis development in both human and mouse were determined. We collected human testis samples from donors of different ages, including children of pre‐puberty (2‐year and 5‐year), peri‐pubety (11‐year) and post‐puberty (17‐year), as well as adult OA patients. In OA testis, ELAVL2 co‐localized with UTF1, GFRA1 and UCHL1, with high expression in both nucleus and cytoplasm (Figure 2A‐C). Both UTF1 and GFRA1 mark SSCs. However, the expression of ELAVL2 rapidly declined in differentiating spermatogonia (c‐KIT^+^) and was completely undetectable in spermatocytes (DMC1^+^) (Figure 2D,E). This expression pattern was consistent and more conspicuous during puberty. Germ cells from the 2‐ and 5‐year‐old samples were GFRA1 positive, and no c‐KIT^+^ spermatogonia were detected (Figure S3A,B). Differentiating spermatogonia (c‐KIT^+^) and meiotic cells began to emerge in the 11‐year‐old sample (Figure S3C,D), and germ cells composition in the 17‐year‐old sample resembled the adult, indicating nearly full spermatogenesis (Figure S3E).^25^, ^34^

**FIGURE 3:**
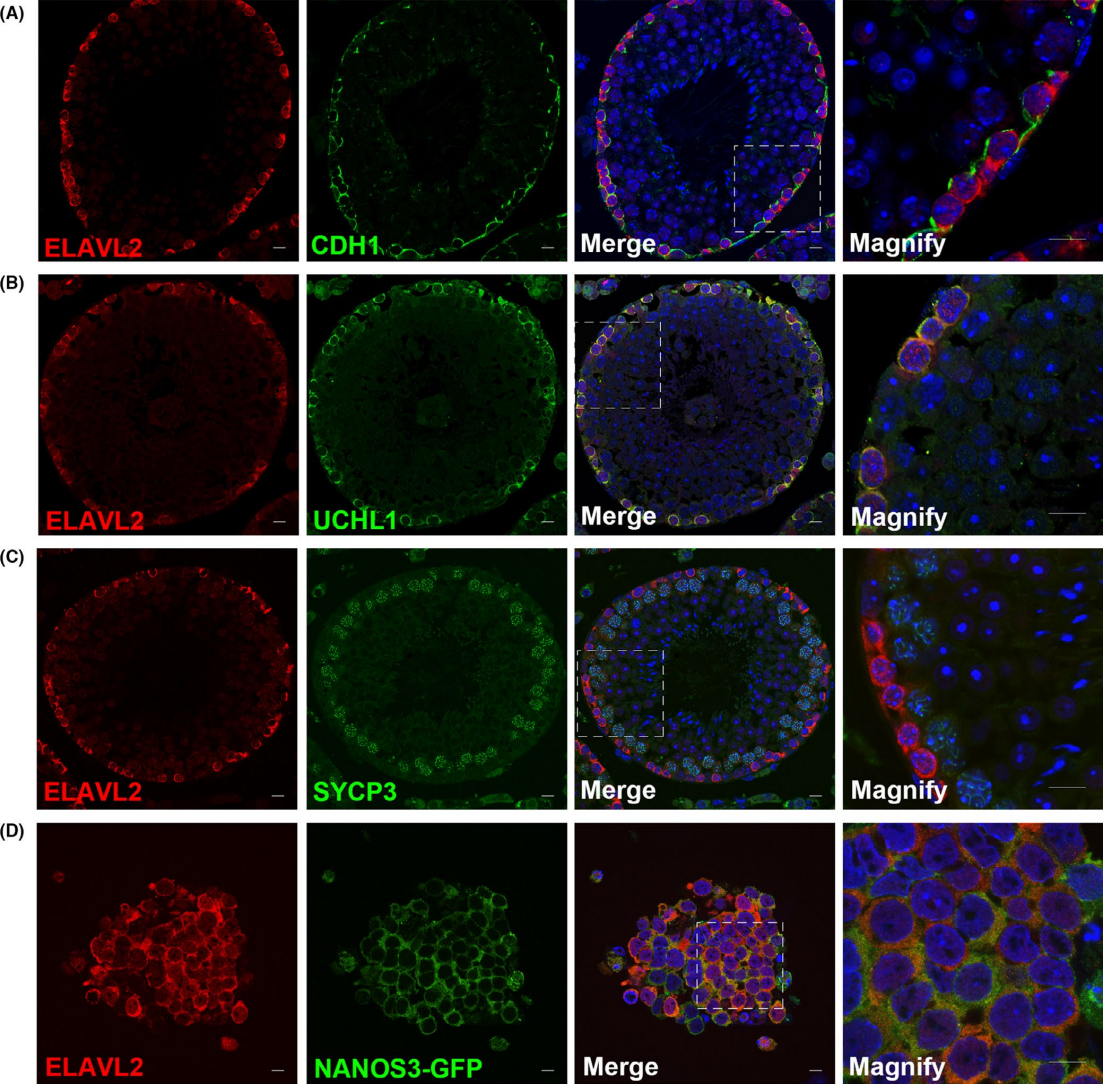
ELAVL2 is enriched in mouse spermatogonial stem cells (SSCs) in adult testis and primary germline stem cells. (A, B) Co‐staining of ELAVL2 with mouse spermatogonia markers, including CDH1 and UCHL1. Scale bar: 20 µm. (C) Co‐staining of ELAVL2 with spermatocyte marker SYCP3. Scale bar: 20 µm. (D) Staining of ELAVL2 in primary germline stem cells derived from *Nanos3‐Gfp* transgenic mouse. Scale bar: 20 µm. Cell nuclei were stained with DAPI

We also explored the expression of ELAVL2 in mouse embryonic testis (E13.5, E15.5, and E17.5) and postnatal testis (P1, P3, P5, P7, P14, and adult). ELAVL2 was expressed in the nucleus and cytoplasm of all gonocytes from E13.5 to P3, while in P5‐P14 and adult testis, ELAVL2 co‐localized with spermatogonia markers UCHL1 and CDH1, and was confined to the basement of seminiferous tubules (Figure 3A,B and Figure S4 and S5). ELAVL2 was undetectable in spermatocytes (SYCP3^+^) and somatic cells of the testis (Figure 3C). Intriguingly, the nucleus expression of ELAVL2 altered during testis development. From P1 to P7, most ELAVL2^+^ cells showed higher nucleus expression than cytoplasm, while it was contrary in P14 and adult testis (Figure 3 and Figure S5). We also detected ELAVL2 expression in primary GS cells, which was derived from P14 *Nanos3‐Gfp* transgenic mouse. It also showed higher ELAVL2 expression in GS cells cytoplasm than nucleus (Figure 3D). The biological basis of this variation is unclear but likely reflects potential roles of ELAVL2 during gonocyte‐to‐SSCs transition and the initiation of the first wave of spermatogenesis.

### 3.3 Establishment of stable cell lines with up‐ or down‐regulated ELAVL2

To explore the functions of ELAVL2 in spermatogonia, stable cell lines with up‐regulated or down‐regulated ELAVL2 were established in C18‐4 and TCam‐2 cells. Observation of EGFP fluorescence and detection of altered ELAVL2 expression indicated the stable cell lines were successfully established. The most efficient shRNA was used in subsequent analyses. The stable cell lines were named as Vector (normal control), ELAVL2 (overexpression), sh‐NC (shRNA control), and shRNA (knockdown), respectively (Figure S6).

### 3.4 ELAVL2 promotes the proliferation of C18‐4 and TCam‐2 cells

**FIGURE 4:**
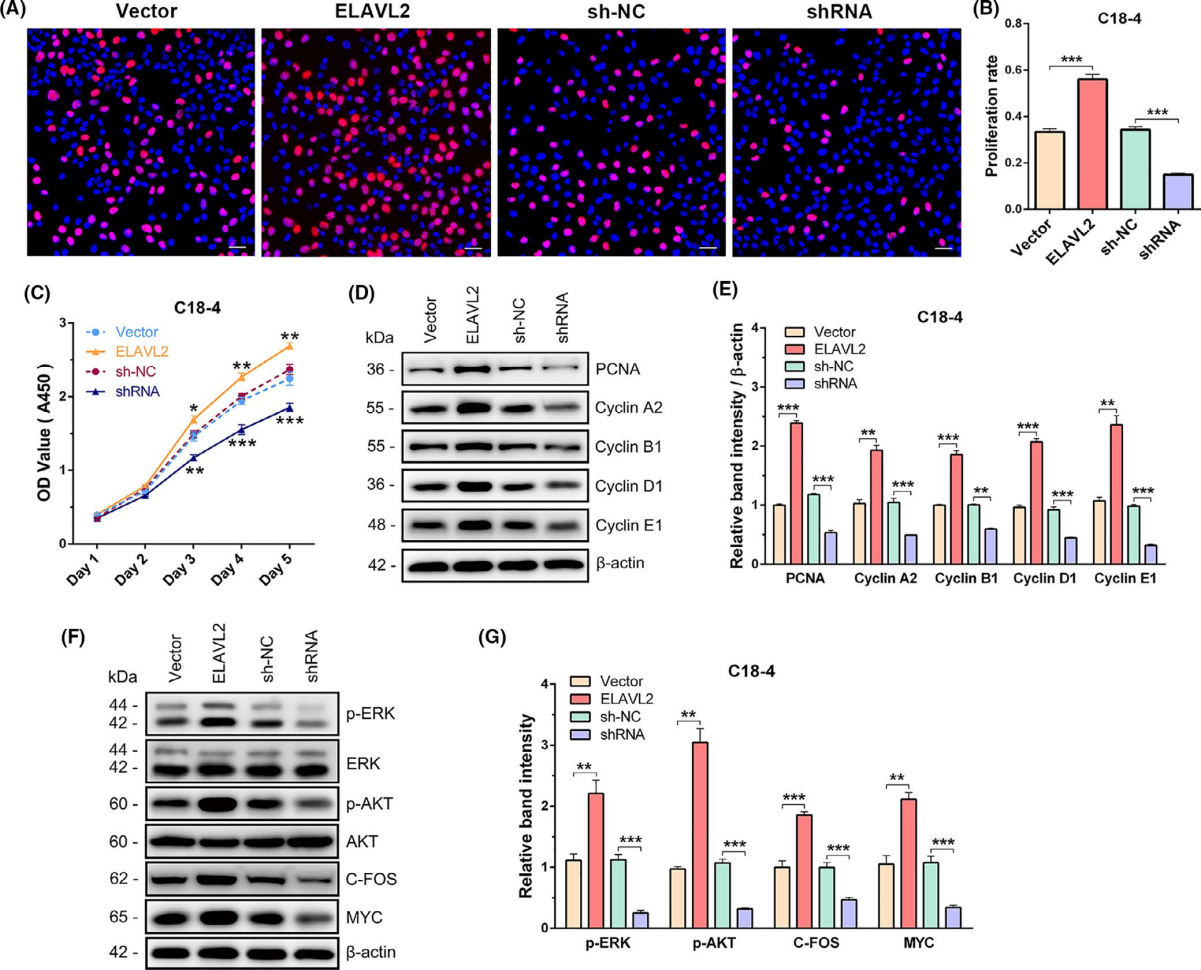
ELAVL2 promotes the proliferation of C18‐4 cells. (A) EDU staining in stable cell lines with up‐ or down‐regulated ELAVL2. Cell nuclei were stained with DAPI. Scale bar: 20 µm. (B) The EDU positive rates of the four stable cell lines. ***P < .001. (C) The growth curve of the four stable cell lines for 5 days revealed by CCK‐8 assay. *P < .05, **P < .01, ***P < .001. (D, E) The protein expressions of PCNA and cell‐cycle proteins (Cyclin A2, Cyclin B1, Cyclin D1, and Cyclin E1) in the stable cell lines (D). The band intensity was normalized to β‐actin (E). **P < .01, ***P < .001. (F, G) The protein expression of p‐ERK, ERK, p‐AKT, AKT, C‐FOS, and MYC in the stable cell lines (F). The band intensity of p‐ERK and p‐AKT were normalized to total ERK and AKT respectively, the band intensity of C‐FOS and MYC were normalized to β‐actin (G). **P < .01, ***P < .001. All error bars show SEM

The balance between proliferation and apoptosis of SSCs is crucial for normal spermatogenesis. We performed multiple methods to detect the proliferation rates of C18‐4 or TCam‐2 stable cell lines. In C18‐4 cells, compared with Vector and sh‐NC, EDU incorporation assay showed that ELAVL2 overexpression increased the EDU‐positive cells per cent, whereas ELAVL2 knockdown reduced the EDU‐positive cells per cent, reflecting that ELAVL2 promoted DNA synthesis of C18‐4 cells (Figure 4A,B). CCK‐8 assay showed that ELAVL2 overexpression significantly promoted the growth rate of C18‐4 cells in a time‐dependent manner, whereas silencing ELAVL2 expression reduced the proliferation (Figure 4C). Next, we examined whether ELAVL2 affected the expression of cell‐cycle regulators by Western blot. Compared with Vector, the expressions of PCNA, Cyclin A2, Cyclin B1, Cyclin D1, and Cyclin E1 were significantly elevated in the ELAVL2 group, whereas the expression of these proteins decreased in the shRNA group (Figure 4D,E).

The results were similar in TCam‐2 cell lines. ELAVL2 significantly promoted TCam‐2 proliferation and increased the expression of cell‐cycle regulators, whereas ELAVL2 knockdown resulted in opposite effects (Figure S8A‐E).

### 3.5 ELAVL2 activates ERK and AKT signalling pathways in C18‐4 and TCam‐2 cells

The proliferation of SSCs is under precise control of various growth factors secreted by Sertoli cells. GDNF and FGF2 are the most known growth factors that regulate SSCs proliferation and self‐renewal via activating ERK and PI3K‐AKT signalling pathways. Therefore, we investigated the effects of ELAVL2 on the activation of the two pathways in C18‐4 cells. Western blot showed that ELAVL2 could significantly increase the phosphorylation of ERK and AKT (Ser473), as well as the expression of downstream effectors C‐FOS and MYC. However, ELAVL2 knockdown inhibited the phosphorylation of ERK and AKT (Ser473), as well as the activation of C‐FOS and MYC (Figure 4F,G). Then, we applied AKT inhibitor (GSK690693) and ERK inhibitor (SCH772984) to C18‐4 cells and detected the changes in cell proliferation. The results showed that both AKT and ERK kinase inhibition decreased the proliferation rates of C18‐4 cells in both control and ELAVL2 overexpressed groups. However, in ELAVL2‐overexpressed cells, the proliferation rates after AKT or ERK inhibition were higher than control cells with AKT or ERK inhibition (Figure S7A). In SSCs, some essential intrinsic factors for SSCs self‐renewal and survival are regulated by AKT and ERK signalling pathways mediated by GDNF or FGF2, such as BCL6B, ETV5, ID4 and NANOS2, while some other factors are independent on GDNF and FGF2 signalling pathways and may act upstream, like PLZF, TAF4b and OCT4. We detected the protein expression of BCL6B, ETV5 and PLZF in the stable C18‐4 cell lines. Also, expression of these downstream factors was also detected after treatment of C18‐4 cells with AKT or ERK kinase inhibitors. The results showed that ELAVL2 could promote the protein expression of BCL6B, ETV5 and PLZF, and either AKT or ERK inhibition could reduce the protein of BCL6B and ETV5, while PLZF protein expression was not affected (Figure S7B).

In TCam‐2 cells, we also observed the activation of ERK and AKT signalling pathways upon ELAVL2 overexpression, which were efficiently inhibited by ELAVL2 knockdown (Figure S8F,G).

### 3.6 ELAVL2 inhibits the apoptosis of C18‐4 and TCam‐2 cells

**FIGURE 5:**
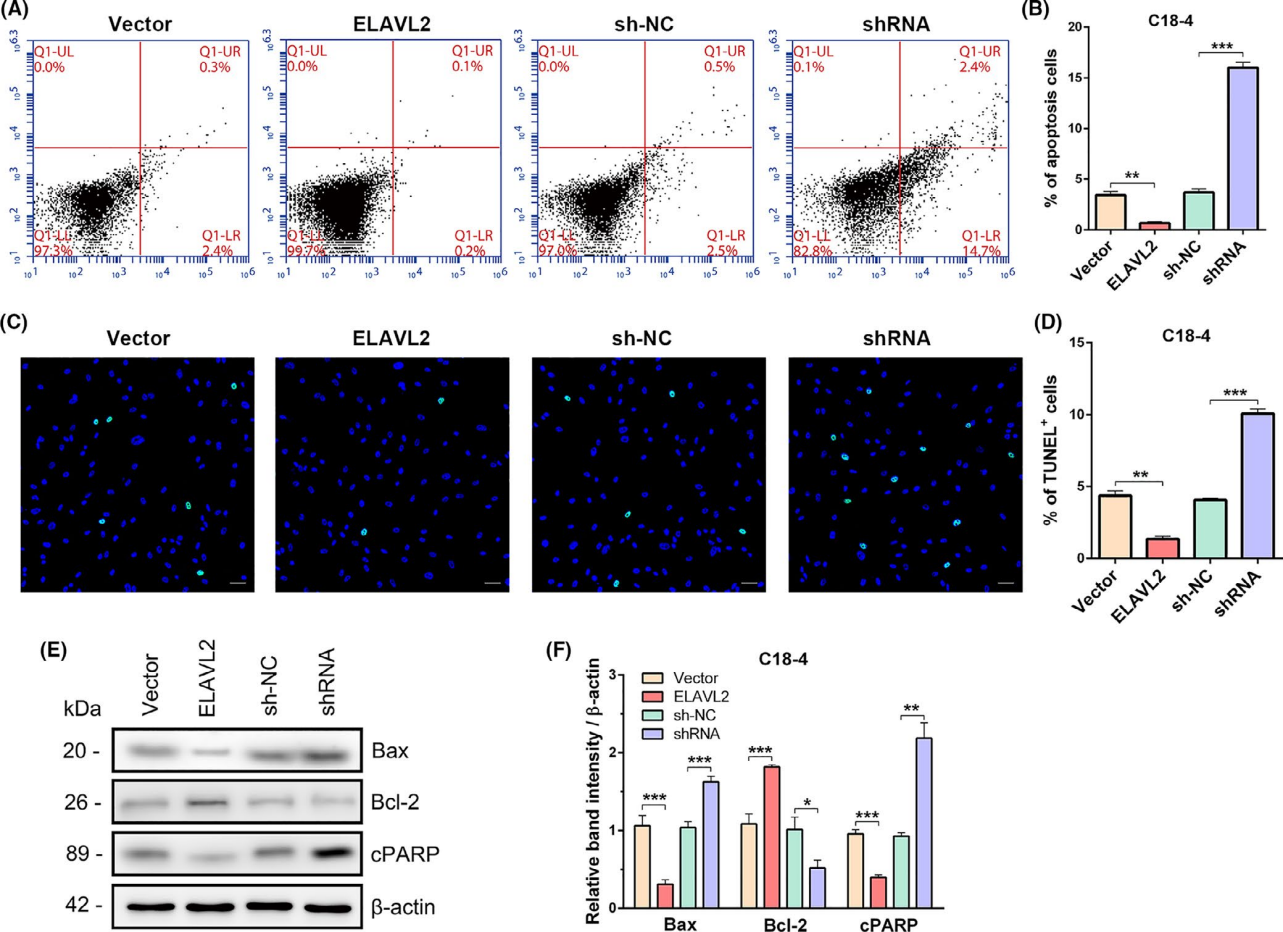
ELAVL2 inhibits the apoptosis of C18‐4 cells. (A, B) Annexin V‐APC/PI and flow cytometry analysis of C18‐4 cells apoptosis. A total of 10 000 cells were analysed. **P < .01, ***P < .001. (C, D) TUNEL assay of apoptotic cells in the four stable C18‐4 cell lines. Scale bar: 20 µm. **P < .01, ***P < .001. (E, F) The protein expression of Bax, Bcl‐2, and cPARP in the stable cell lines (E). The band intensity was normalized to β‐actin (F). *P < .05, **P < .01, ***P < .001. All error bars show SEM

Next, we examined the influence of ELAVL2 on the apoptosis of spermatogonia. In C18‐4 cells, Annexin V/propidium iodide (PI) staining and flow cytometry showed that the percentage of apoptosis decreased from 3.43% ± 0.371% to 0.67% ± 0.15% after ELAVL2 upregulation and increased from 3.70% ± 0.36% to 16.00% ± 0.56% when ELAVL2 was knocked down (Figure 5A,B). The percentage of TUNEL^+^ cells significantly reduced when ELAVL2 was overexpressed, and increased after ELAVL2 inhibition (Figure 5C,D). Furthermore, ELAVL2 overexpression resulted in a decrease in protein levels of apoptosis indicators cleaved‐PARP (cPARP) and Bax, whereas down‐regulation of ELAVL2 increased cPARP and Bax expression (Figure 5E,F). Meanwhile, the expression of Bcl2, which suppresses apoptosis, was significantly increased in the ELAVL2 group and decreased in the shRNA group (Figure 5E,F).

The similar results were also observed in TCam‐2 cells. ELAVL2 inhibited early apoptosis, reflecting by decreased apoptotic rate, positive TUNEL rate, and expression of cPARP and Bax, as well as elevated Bcl‐2 expression. However, ELAVL2 knockdown led to significantly increased cell apoptosis (Figure S9).

### 3.7 ELAVL2 associates with mRNAs that encode essential regulators of SSCs proliferation and maintenance

**FIGURE 6:**
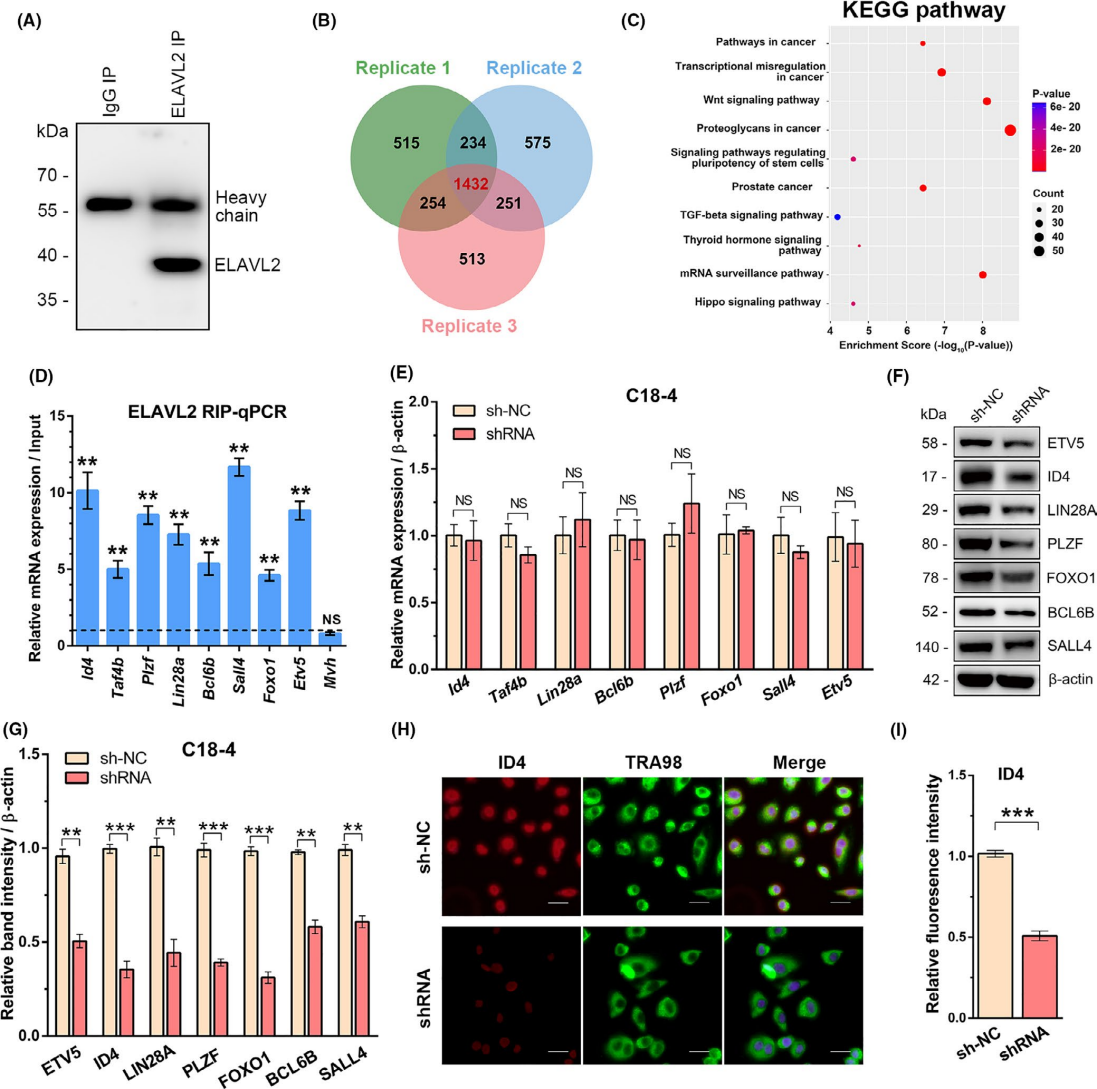
ELAVL2 associates with mRNAs that encode essential regulators of spermatogonial stem cells (SSCs) proliferation and maintenance. (A) Immunoblot analysis of ELAVL2 immunoprecipitation using mouse testes. (B) Venn diagram showing the overlap of ELAVL2‐bound genes among three replicates. (C) KEGG pathway analysis of the overlapped targets of ELAVL2 among three replicates by DAVID functional annotation tools. (D) RIP‐qPCR analysis of 8 ELAVL2 potential targets in mouse testis, with *Mvh* as negative control, which was not the target of ELAVL2. Relative enrichment in ELAVL2‐IP was compared with Input. **P < .01. NS: No significant difference. (E) qPCR assay to compare the mRNA levels of indicated ELAVL2 targets in C18‐4 cells between the control group and the shRNA group with ELAVL2 knockdown, with *β‐actin* as internal control. NS: No significant difference. (F, G) Western blot assay to compare the protein expressions of indicated ELAVL2 targets in C18‐4 cells between the control group and the shRNA group with ELAVL2 knockdown (F). The band intensity was normalized to β‐actin (G). **P < .01, ***P < .001. (H, I) Immunostaining of ELAVL2 target ID4 in C18‐4 cells was compared between the control group and the shRNA group with ELAVL2 knockdown. Fluorescence intensity was normalized to germ cells marker TRA98. Cell nuclei were stained with DAPI. ***P < .001. Scale bar: 20 µm. All error bars show SEM

To shed light on how ELAVL2 functions during spermatogenesis, we performed sequencing of RNAs isolated by RNA immunoprecipitation (RIP‐Seq) to identify ELAVL2 binding targets. ELAVL2 antibody was used to pull‐down RNAs from P14 mice testes. Western blot analysis confirmed successful immunoprecipitation of ELAVL2 (Figure 6A). As a result, 1,432 genes were identified as putative targets, which were overlapped across the three replicates (Figure 6B, Table S4). GO analysis was carried out to explore the cellular events that ELAVL2‐associating mRNAs are involved in, which were enriched in transcription regulation, transcription factor activity, DNA binding, and chromatin binding (Figure S10A‐C). These putative targets were also significantly enriched for pathways involved in transcription regulation, Wnt signalling, pluripotency regulation of stem cells and mRNA surveillance (Figure 6C, Table S5).

Strikingly, among the mRNAs bound by ELAVL2, many have been intensively studied and widely recognized as indispensable for SSCs proliferation and maintenance. Out of 13 genes shown to be important for the maintenance of SSCs, 8 genes were found to be ELAVL2 targets, including *Id4*, *Taf4b*, *Plzf*, *Lin28a*, *Bcl6b*, *Sall4*, *Foxo1* and *Etv5*.^35^, ^36^, ^37^, ^38^, ^39^, ^40^, ^41^, ^42^ RIP and qPCR validate the results of ELAVL2 immunoprecipitation (Figure 6D). Next, we examined mRNA and protein expression of ELAVL2 targets in C18‐4 cell lines. Compared with the control group, ELAVL2 knockdown did not affect their mRNA levels, but significantly reduced protein expressions (Figure 6E‐G). Immunofluorescence confirmed the results. Based on fluorescence intensity relative to the germ cell marker TRA98, the expression of ID4, SALL4, ETV5, LIN28A and PLZF showed a statistically significant decrease compared to control (Figure 6H,I and Figure S10D‐G). These findings showed that ELAVL2 regulates spermatogonia proliferation through promoting the protein expression of target genes. Then, we explored whether the defects of proliferation in ELAVL2 knockdown C18‐4 cells could be rescued by target genes. As PLZF is essential for SSCs maintenance and survival, we overexpressed PLZF in C18‐4 cells with ELAVL2 knockdown. CCK‐8 and EDU incorporation assay showed that PLZF promoted the proliferation of C18‐4 cells with ELAVL2 knockdown, but the proliferation rate was still lower than the control group (Figure S10H, I).

### 3.8 ELAVL2 interacts with DAZL in human and mouse testes

**FIGURE 7:**
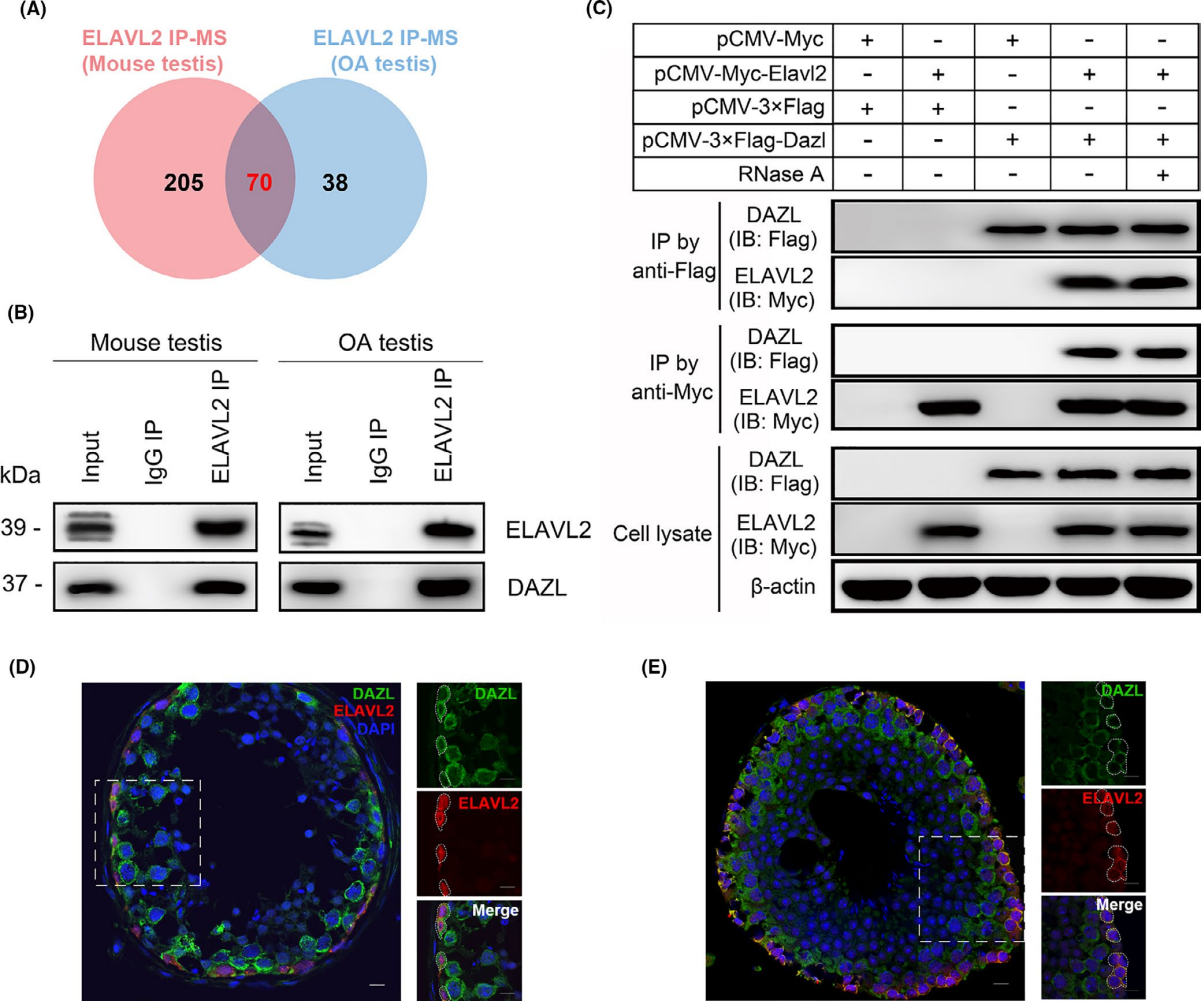
ELAVL2 interacts with DAZL in human and mouse spermatogonial stem cells (SSCs). (A) Venn diagram showing the proteins enriched in ELAVL2‐IP complex in mouse and OA (obstructive azoospermia) testis tissues by mass spectrometry analysis. (B) IP assay by anti‐ELAVL2 antibody showing the interaction between ELAVL2 and DAZL in both mouse and OA testis tissues. (C) IP assay by anti‐Myc or anti‐Flag antibody showing the interaction between transfected Myc‐ELAVL2 and Flag‐DAZL fusion proteins with or without RNase A treatment in HEK‐293T cells. (D) Co‐localization of ELAVL2 and DAZL in human testis revealed by immunofluorescence staining (white dotted line). Scale bar: 20 µm. (E) Colocalization of ELAVL2 and DAZL in mouse testis revealed by immunofluorescence staining (white dotted line). Cell nuclei were stained with DAPI. Scale bar: 20 µm

Next, we intended to search for proteins that interact with ELAVL2. Anti‐ELAVL2 antibody or normal IgG were used for immunoprecipitation in both OA testis sample and P14 mouse testis. The precipitates were applied to mass spectrometric analysis, which identified 275 proteins in mouse and 108 proteins in human that potentially interact with ELAVL2, and 70 of them were overlapped (Figure 7A, Table S6). Gene ontology (GO) analysis revealed the 70 proteins were enriched in the regulation of translation and mRNA metabolism, like mRNA stabilization and mRNA splicing. They were also significantly enriched for pathways involved in spliceosome, ribosome, RNA transport, and mRNA surveillance (Figure S11A‐D, Table S7). Among them, DAZL (Deleted in Azoospermia‐like) is one of the most known proteins that regulates spermatogenesis, including SSCs maintenance and proliferation. DAZL is a strong candidate for the azoospermic factor, and various polymorphisms of DAZL have been implicated to be associated with azoospermia. Also, DAZL is conserved between human and mouse, indicating a universal role in regulating spermatogenesis in different species (Figure S11E).^43^, ^44^, ^45^

To determine if ELAVL2 directly interacts with DAZL, we performed co‐IP of ELAVL2 and DAZL in OA testis and P14 mouse testis extract. ELAVL2 could efficiently pull down DAZL, establishing a direct interaction between these two proteins in vivo (Figure 7B). We also constructed Myc‐tagged ELAVL2 and 3 × Flag‐tagged DAZL plasmids, and transfected them into HEK‐293T cells in various combinations with empty vectors. Co‐IP was performed using anti‐Myc or anti‐Flag antibodies, which showed that ELAVL2 and DAZL reciprocally pulled down each other with or without RNase A, establishing a direct interaction between the two proteins in vitro independent of RNA (Figure 7C). Furthermore, immunofluorescent staining of ELAVL2 and DAZL revealed their co‐localization in the cytoplasm of human and mouse SSCs (Figure 7D,E).

## 4 DISCUSSION

The pathological mechanism, diagnosis, and treatment of NOA is still a clinical challenge, which largely resulted from our limited understanding of the intricate human spermatogenesis. As the stem cells supporting continuous sperms production throughout life, the well‐balanced SSCs fate determination is of great significance for normal spermatogenesis, including the balance between self‐renewal and differentiation, proliferation and apoptosis.^46^ Although transcription factors and signalling molecules regulating SSCs fate determinations have been reported, the importance of post‐transcriptional gene regulation has not been addressed, especially in human SSCs.^47^, ^48^ In this study, we identified ELAVL2 as a conserved RBP required for the proliferation and survival of human and mouse spermatogonia. In vitro studies showed that ELAVL2 could promote spermatogonia proliferation via activating ERK and AKT signalling pathways, and inhibit the apoptosis of spermatogonia. Mechanically, ELAVL2 interacted with DAZL in both human and mouse testes, an essential RBP regulating SSCs self‐renewal and meiosis initiation. ELAVL2 also promoted protein expression of target genes, including several genes that have been extensively studied and recognized indispensable for the proliferation and survival of SSCs.

The exploration of NOA pathogenesis has been difficult in a long time, due to the lack of reliable approaches for human spermatogenesis study. Previously, we identified seven susceptibility loci for NOA in Han Chinese men using genome‐wide association study (GWAS).^3^, ^49^ Loss‐of‐function (LoF) gene mutations were also found in some NOA patients via whole exome sequencing (WES), which advanced our understanding of the genetic susceptibility to NOA.^50^, ^51^, ^52^ The comparison of Sertoli cells transcriptome by next‐generation sequencing (NGS) and single‐cell RNA sequencing (scRNA‐seq) revealed the essential role of aberrant Sertoli cells functions in NOA, and instructed us to find out novel extracellular signalling molecules that regulate SSCs proliferation and survival.^25^, ^53^, ^54^ Nevertheless, the pathogenic factors for a large portion of NOA patients are still unclear, and the intrinsic factors regulating human SSCs self‐renewal and differentiation remain elusive. The fundamental role of SSCs in human spermatogenesis is widely recognized, but the researches of human SSCs are hindered by two challenges, the efficient isolation of human SSCs and the long‐term expansion of SSCs in vitro. MACS and FACS have been applied to sort human SSCs based on surface markers, which are too limited to discriminate every stage of spermatogonia, and may interrupt normal cell transcriptome.^55^, ^56^ In vitro culture of isolated human SSCs only lasted a short period and is incapable for further function and mechanism studies. The scRNA‐seq provides a nice solution to human SSCs study, which could unveil the existence of spermatogonia heterogeneity and reveal the dynamic transcriptome transition during SSCs development. It also helped to reveal the difference and equivalence of SSCs development among various species, from rodents to higher primates.^5^, ^57^, ^58^ However, the application of scRNA‐seq in NOA patients is rarely reported. In this and previous studies, we conducted scRNA‐seq of both NOA and OA testis tissues. Based on distinct transcriptome characteristics, we identified five clusters of human spermatogonia, including three stages of SSCs (SSC1, SSC2 and SSC3), differentiating spermatogonia, and differentiated spermatogonia.^25^ The scRNA‐seq also provided high resolution to find the differentially expressed genes of each remaining cell type between NOA and OA patients, which could not be revealed via classical pathological analysis or genetic screening. The tissue‐specific or cell‐specific expression of genes usually indicate their biological functions. Therefore, we focused on genes that enriched in testis and spermatogonia, which were also down‐regulated in NOA spermatogonia meanwhile. Through this unbiased analysis, we screened out 14 genes, including RBPs (ELAVL2, PIWIL2, IGF2BP1, and TDRD1), DNA‐binding or chromatin binding proteins, ubiquitin ligase, etc After extensive literature review and protein expression validation, we focused on ELAVL2 ultimately, which is a conserved RBP and enriched in human and mouse SSCs.

Strikingly, there are several noteworthy findings about the ELAVL2 expression. First, ELAVL2 is highly expressed in early male germline stem cells. In mouse, ELAVL2 expression could trace back to gonocytes in E13.5 testis, which maintained at high level during testis development but declined rapidly after SSCs differentiation. It was the same during human testis development, but we did not know whether ELAVL2 also expressed in human gonocytes due to the lack of human embryonic testis samples. Second, the subcellular localization of ELAVL2 in SSCs was different between human and mouse. From P1 to P7, most ELAVL2^+^ cells in mouse testis showed higher expression in the nucleus than in the cytoplasm, while it was contrary in P14 and adult testis. During human testis development, ELAVL2 was expressed in both the nucleus and cytoplasm of SSCs, with higher expression in the nucleus of most SSCs. The difference in seminiferous epithelium cycle and dynamics of SSCs self‐renewal and differentiation between human and mouse may contribute to this variation. Third, ELAVL2 showed high expression in human seminoma tissue and TCam‐2 cell line that generated from pure seminoma. TCam‐2 cells resemble early male germline stem cells in phenotype and express several gonocytes and SSCs markers, including SALL4, POU5F1, BLIMP1 and NANOG.^32^ As there has been no recognized human SSCs cell line, and in vivo study in human testis is unpractical, we used TCam‐2 cell line as an in vitro model to study the functions rather than mechanisms of ELAVL2.

Our in vitro functional studies showed that ELAVL2 promoted proliferation and inhibited apoptosis of C18‐4 and TCam‐2 cells via the activation of ERK and AKT signalling pathways. ERK1/2 and AKT activation represents significant signals for the regulation of cellular growth and proliferation by phosphorylating specific transcription factors in the nuclei. Previous studies revealed that many extrinsic growth factors promoted mouse SSCs proliferation or self‐renewal via these two pathways, including GDNF, FGF2 and FGF5.^9^, ^53^, ^59^ Conditional activation of AKT without the addition of GDNF could also induce the proliferation of SSCs, suggesting AKT pathway was a prerequisite for the self‐renewal of mouse SSCs.^60^ In this study, ELAVL2 significantly activated ERK and AKT (Ser473) by phosphorylation, and increased protein expression of C‐FOS and MYC, two essential downstream regulators of cell proliferation and apoptosis. AKT and ERK inhibitors were also applied to validate the results. Previous studies showed that some essential intrinsic factors for SSCs self‐renewal and survival are regulated by AKT and ERK signalling pathways mediated by GDNF or FGF2, such as BCL6B, ETV5, ID4 and NANOS2, while some other factors are independent on GDNF and FGF2 signalling pathways and may act upstream, like PLZF, TAF4b and OCT4.^61^, ^62^ Therefore, we detected the protein expression of BCL6B, ETV5 and PLZF in the stable C18‐4 cell lines with or without AKT and ERK kinase inhibition. The results showed that ELAVL2 could promote the protein expression of BCL6B, ETV5 and PLZF, and either AKT or ERK inhibition could reduce the protein of BCL6B and ETV5, while PLZF protein expression was not affected. As BCL6B, ETV5 and PLZF were all putative targets of ELAVL2, the protein expression of BCL6B and ETV5 may be regulated directly by ELAVL2 or indirectly through AKT and ERK signalling pathways, but PLZF expression was probably directly regulated by ELAVL2. However, whether there were interactions between ELAVL2‐mediated signalling and conventional GDNF or FGF2‐mediated signalling is unknown.

As proteins that recognize and bind specific transcripts, RBPs fulfil their functions through post‐transcriptional regulation of target mRNAs and synergistic action with other proteins.^15^ To gain insights into molecular mechanisms of ELAVL2 in regulating spermatogonia proliferation and apoptosis, we performed RIP‐seq with P14 mouse testis, which identified more than 1,000 targets of ELAVL2. Notably, transcripts regulating SSCs self‐renewal and survival were highly enriched among ELAVL2 targets, including *Id4*, *Taf4b*, *Plzf*, *Lin28a*, *Bcl6b*, *Sall4*, *Foxo1* and *Etv5*. Former studies revealed that ELAVL2 promotes translation of targeting mRNAs containing AU‐rich elements by accelerating the formation of translation initiation complexes, like GLUT1 in adipocytes, DDX6 in oocytes, and neurofilament M in human teratocarcinoma cells.^30^, ^63^, ^64^ Similarly, our results also showed that ELAVL2 promoted the translation of target mRNAs without apparent effect on mRNA levels. ID4 is an important transcription factor induced by GDNF and is exclusively detected in SSCs. ID4 knockout mice exhibit age‐dependent germ cell depletion, suggesting a role in SSCs maintenance.^35^, ^65^ TAF4b performs an essential germ cell autonomous function in SSCs establishment and/or maintenance. TAF4b‐deficient spermatogenic progenitor cells display a tendency for differentiation at the expense of self‐renewal and a renewing pool of SSCs fail to establish during the critical window of SSCs development.^36^, ^66^ PLZF was first described as an important maintenance factor specifically expressed in SSCs, which could repress the expression of c‑Kit and mTORC1, markers for spermatogonia differentiation. Nonsense mutations in PLZF cause infertility and progressive germ cell loss in mice.^39^, ^67^, ^68^ LIN28A is an RNA‐binding pluripotent stem cell factor, and is strongly expressed in mouse and primate SSCs. LIN28A represses let‐7 microRNAs and influences mRNA translation, thereby regulating the self‐renewal of mammalian embryonic stem cells and primordial germ cells specification.^38^, ^69^ BCL6B is another GDNF‐inducible factor essential for SSCs maintenance. Loss of BCL6B function results in impaired spermatogenesis in vivo and SSCs clump maintenance in vitro.^42^ SALL4 expression in adults is restricted to spermatogonia, and is required for long‐term maintenance of SSCs by repressing expression of Foxl1 and Dusp4, two tumour suppressor genes that inhibit SSCs growth and self‐renewal.^41^, ^70^ FOXO1 plays an indispensable role in SSCs maintenance, and may be at the upper levels of GDNF‐independent transcription regulation. FOXO1 knockout mice exhibit a progressive age‐dependent decline in spermatogenesis, similar to PLZF and TAF4b.^40^ ETV5 was found to be a critical downstream regulator of GDNF signalling that mediated the expression of several known SSC self‐renewal related genes, and Etv5 ablation in mice also causes infertility, although the first wave of spermatogenesis is not impaired, the following waves are severely impaired.^37^, ^71^ These findings showed that ELAVL2 regulates spermatogonia proliferation through promoting the protein expression of target genes. However, the RIP‐seq was performed using mouse testes; the targets of ELAVL2 in human SSCs are still unclear.

In order to find out proteins that may interact with ELAVL2, we conducted immunoprecipitation and mass spectrum analysis in both human and mouse testis tissues. Among the overlapped proteins, we focused on DAZL, another RBP recognized indispensable for mouse SSCs self‐renewal and survival. Next we validated the interaction between ELAVL2 and DAZL in both human and mouse testis, which showed the two RBPs bonded with each other in vivo and in vitro, and co‐localized in human and mouse SSCs. DAZL is a hallmark of vertebrate germ cells. Stage‐specific deletion of *Dazl* in mouse germ cells did not affect female fertility, but caused complete male sterility with gradual loss of SSCs, meiotic arrest and spermatid arrest. DAZL bound thousands of mRNAs via GUU sites upstream of polyA tails, and loss of DAZL resulted in decreased mRNA levels for a network of genes that are essential for germ cell proliferation and differentiation.^43^ In mouse testis, DAZL did not affect the initial pool of SSCs, but was involved in SSCs self‐renewal and development by promoting SSCs‐associated targets protein expression, including PLZF, LIN28A, and FOXO1, which were also targets of ELAVL2.^43^, ^44^

**FIGURE 8:**
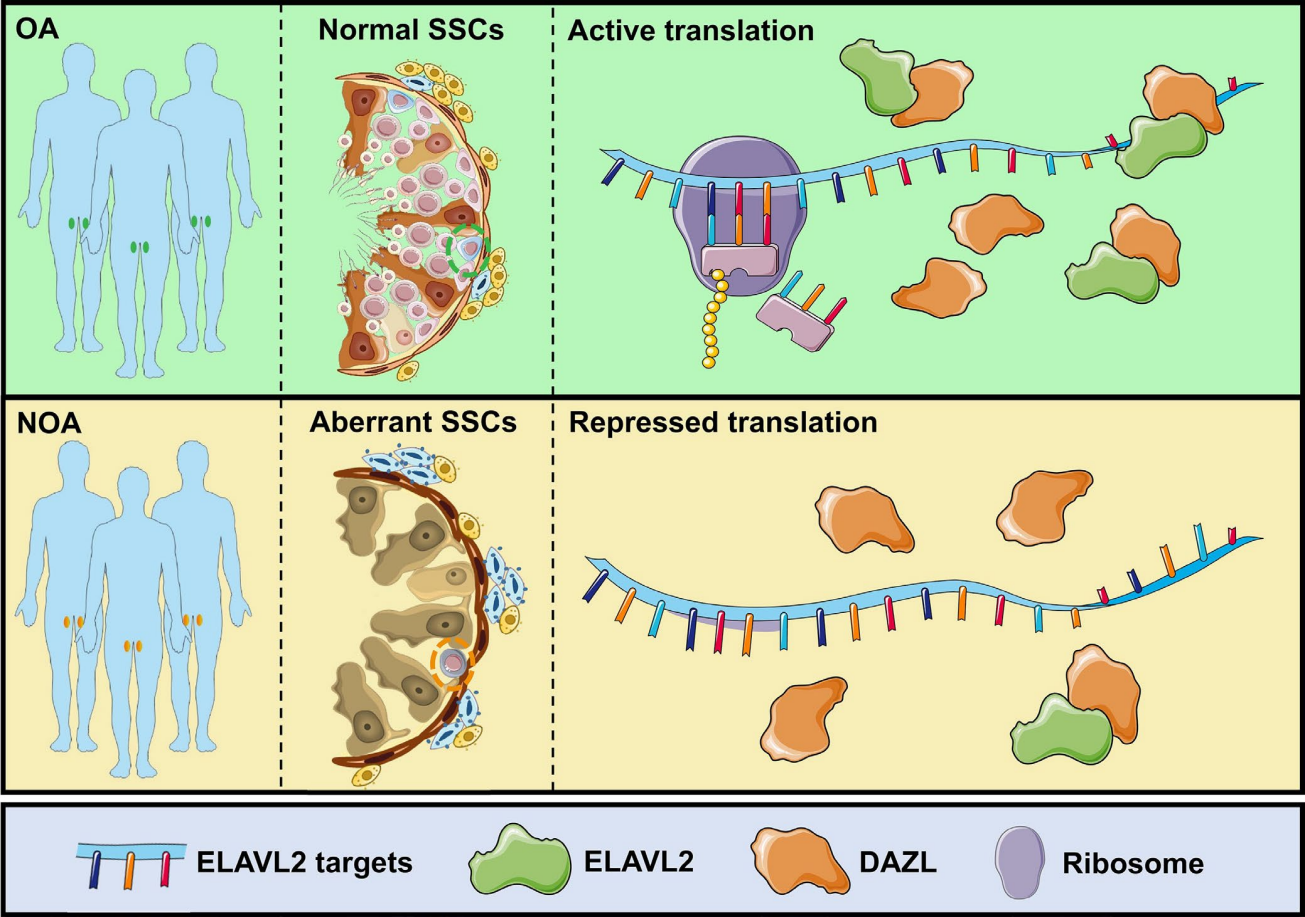
Diagram of proposed functions and mechanisms of ELAVL2. ELAVL2 is enriched in human spermatogonial stem cells (SSCs) and down‐regulated in NOA SSCs. ELAVL2 is essential for spermatogonia proliferation and survival, which promotes protein expression of target genes

Although our scRNA‐seq results and in vitro studies showed the regulatory functions of ELAVL2 in spermatogonia proliferation and survival, it is worth noting that *Elavl2* knockout did not impair mouse spermatogenesis according to a previous study. In this study, inactivation of ELAVL2 caused the defect of female germ cell development, whereas the development of male germ cell is not affected.^30^ The underlying mechanisms are still unknown, but there are several hypotheses. First, human and mouse spermatogenesis had a great difference, even highly conserved genes may have different evolutionary functions. For example, pathogenic mutations of many genes have rarely been identified and reported in infertile male patients, even though they are enriched in germ cells and their functions and mechanisms have been thoroughly studied with mouse inactivation models, like DND1, UTF1, PIWIL2, etc Also, proteins that interact with ELAVL2 in human or mouse testis are different, as revealed by our immunoprecipitation and mass spectrum results. In addition, it is possible that transcripts targeted and regulated by ELAVL2 in human and mouse testis are different, although we did not conduct ELAVL2 RIP‐seq with human testis tissues. Second, functional compensation by other molecules or pathways may exist, leading to the difference between in vivo and in vitro experiments. Nevertheless, our scRNA‐seq results that directly targeting OA and NOA patients could provide novel information for exploring the mechanisms of human spermatogenesis and azoospermia, which could make up for the deficiency of animal model studies. In conclusion, we identified ELAVL2 as a conserved RBP enriched in both human and mouse testes and SSCs, which was down‐regulated in NOA. Functional studies and mechanisms exploration demonstrated ELAVL2‐regulated spermatogonia proliferation and apoptosis by promoting protein expression of target genes, and the functions of ELAVL2 may be fulfilled by interacting with DAZL (Figure 8). Thus, our study provides a framework to understand the molecular basis of post‐transcriptional gene regulation in spermatogenesis, unveils novel pathogenesis of NOA, and provides new target for potential therapy in the future.

## CONFLICT OF INTEREST

There is no conflict of interest to declare.

## AUTHOR CONTRIBUTIONS

ZL and ZZ designed the study, and revised the manuscript. CY, CY, and ZJ performed the experiments, acquired data and drafted the article. LZ, HC, and PL analysed and interpreted the data. RT, EZ, and YH provided reagents and materials tools. YH and XH collected relevant papers in this field.

## Supporting information

Table S1

Table S2

Table S3

Table S4

Table S5

Table S6

Table S7

Table S8

Appendix S1

Appendix S2

Appendix S3

## Data Availability

The data that support the findings of this study are available from the corresponding author upon reasonable request.
