# Supplementary material for: RNA‐binding protein ELAVL2 plays post‐transcriptional roles in the regulation of spermatogonia proliferation and apoptosis
Source: Cell Prolif. 2021 Jul 23;54(9):e13098. doi: 10.1111/cpr.13098 (PMC8450129; doi:10.1111/cpr.13098)
Supplement: Supplementary file 9 — Appendix S1 [file CPR-54-e13098-s002.docx]

**Supplementary materials and methods**

**RNA-binding protein ELAVL2 plays post-transcriptional roles in the regulation of spermatogonia proliferation and apoptosis**

Chao Yang^1,4^, Chencheng Yao^1,4^, Zhiyong Ji^2,4^, Liangyu Zhao^1^, Huixing Chen^1^, Peng Li^1^, Ruhui Tian^1^, Erlei Zhi^1^, Yuhua Huang^1^, Xia Han^1^, Yan Hong^1^, Zhi Zhou^3^, Zheng Li^1,2^

^1^ Department of Andrology, the Center for Men’s Health, Urologic Medical Center, Shanghai General Hospital, Shanghai Jiao Tong University School of Medicine, Shanghai, China

^2^ State Key Lab of Reproductive Medicine, Nanjing Medical University, Nanjing, China

^3^ School of Life Science and Technology, ShanghaiTech University, Shanghai, China

^4^ These authors contributed equally to this work

**Animals**

All animal care and experiments were performed according to the guidelines of the National Institutes of Health and approved by the Animal Care Committee of Shanghai General Hospital. For expression analysis of ELAVL2, we chose male C57BL/6 mice at different developmental time points, including P1, P3, P5, P7, P14, and P56. Embryonic testes were taken from the embryo of C57BL/6 mice at E13.5, E15.5, and E17.5. RIP-seq and IP-MS of ELAVL2 each used 6 testes from P14 male C57BL/6 mice.

**Immunocytochemistry**

For immunocytochemistry staining, cells were fixed with 4% PFA for 30 min, washed three times with cold PBS (Gibco), and permeabilized with 0.4% Triton X-100 (Sigma) for 5 min. After extensive wash with PBS, the cells were blocked in 5% bovine serum albumin (BSA) (Sigma) for an hour at room temperature. The cells were then incubated with primary antibodies overnight at 4 ℃ (Table S8). Antigen detection was conducted using the appropriate combination of Alexa Fluor 488 and 594 secondary antibodies (Table S8) for 1 hour at room temperature in the dark. DAPI was used to label the nuclei. Images were captured with a OLYMPUS confocal microscope.

**Immunostaining of testicular and seminoma Tissues**

The immunofluorescence (IF) staining was performed on 5 μm formalin-fixed paraffin embedded (FFPE) sections from portions of the collected testicular and seminoma samples following deparaffinisation, rehydratation and heat-mediated antigen retrieval in 10 mM sodium citrate buffer solution (pH 6). After treatment with 5% BSA for 1 hour at room temperature, individual sections were incubated overnight at 4 °C with a mix of diluted antibodies (Table S8). Antigen detection was conducted using the appropriate combination of Alexa Fluor 488 and 594 secondary antibodies (Table S8) for 1 hour at room temperature in the dark. DAPI was used to label the nuclei. Images were captured with a OLYMPUS confocal microscope.

**RNA extraction, RT-PCR, and real-time qPCR**

Total RNA was extracted from cultured cells or tissues using TRIzol (Takara, Kusatsu, Japan), and the quality and concentrations of total RNA were measured by NanoDrop (Thermo Fisher Scientific, USA). The ratio of A260/A280 of total RNA was set as 1.9~2.0 to ensure quality.

Reverse transcription (RT) of total RNA was conducted using the First Strand cDNA Synthesis Kit (Thermo Fisher Scientific, USA), and PCR of the cDNA was carried out according to the protocol as described previously. The primer sequences of chosen genes were designed and listed in Table S8. The PCR started at 94 ℃ for 2 min and was performed in terms of the following conditions: denaturation at 94 ℃ for 30 s, annealing at 55 ℃~60 ℃ for 45 s, and elongation at 72 ℃ for 45 s, for 35 cycles. The samples were incubated for an additional 5 min at 72 ℃. PCR with PBS but without cDNA served as a negative control. PCR products were separated by electrophoresis on 2% agarose gel and visualized with ethidium bromide. Images were recorded and band intensity was analyzed using chemiluminescence (Chemi-Doc XRS; Bio-Rad).

Real-time qPCR was performed using Power SYBR Green PCR Master Mix (Applied Biosystems, UK) in a Veriti 96-Well Thermal Cycler. To quantify the PCR products, we used the comparative Ct (threshold cycle) method as described previously. The threshold of cycle values of genes was normalized against the threshold value of human housekeeping gene *β-actin* [ΔCt= Ct (target gene) − Ct (*β-actin*)], and the relative expression of target genes in treatment group to the controls was calculated by formula 2^−ΔΔCt^ [ΔΔCt=ΔCt (treatment) – ΔCt (control)]. The primers of detected genes were listed in Table S8.

**Western blot**

Tissues and cultured cells were lysed with RIPA buffer (BiotechWell, Guangzhou, Shanghai, China) for 30 min on ice. Tissues were homogenized before lysis. The lysates were centrifuged at 12,000 g for 20 min at 4 °C, and the protein concentration was measured using BCA kit (Dingguo Changsheng Biotech, Beijing, China). Twenty micrograms of lysate from each sample were used for SDS-PAGE (Bio-Rad Laboratories), and western blots were performed according to the protocol as described previously. Briefly, samples were resolved in the XCell Sure Lock Novex Mini-Cell apparatus (Invitrogen, Carlsbad, USA) and transferred to nitrocellulose membranes for 1.5 hours on ice. The membranes were washed with TBS containing 0.1% Tween (TBST) and blocked with 5% non-fat dry milk in TBST for 1 hour at room temperature. After extensive wash with TBST, the membranes were incubated with the chosen primary antibodies overnight at 4 °C (Table S8). Next day, the membranes were washed three times with TBST and then incubated with HRP-conjugated immunoglobulin G (IgG) at a 1:2000 dilution for 1 hour at room temperature (Table S8). The blots were detected by chemiluminescence (Chemi-Doc XRS, Bio-Rad, Hercules, CA, USA) after extensive washes with TBST. Optical density analysis was processed with Adobe Photoshop CC software. The relative band density was normalized to β-actin.

**Cell proliferation assay**

Cultured stable cell lines were seeded at a density of 1,000 cells/well in 96-well microtiter plates in DMEM/F-12 supplemented with 10% FBS overnight. The medium was changed every day. The proliferation potential of C18-4 and TCam-2 cells were detected by CCK-8 assay (Dojin Laboratories, Kumamoto, Japan) for 5 days according to the manufacturer’s instruction.

For EDU incorporation assay, cultured stable cell lines were seeded in 96-well plates with 1,000 cells/well in DMEM/F-12 supplemented with 10% FBS overnight to allow the cells to attach. Then, 20 μΜ of 5-Ethynyl-2′-Deoxyuridine (EDU) (RiboBio, Guangzhou, China) were added to the medium and incubated for 2 hours. Afterwards, the cells were washed twice with PBS and fixed with 4% PFA at room temperature, and 50 μl of 2 mg/ml glycine was added to each well to neutralize the PFA. The cells were washed with 0.5% Tritonx-100 in PBS and exposed to 100 μl Apollo-Fluor for 30 min in the dark at room temperature. Cell nuclei were stained with DAPI for 30 min. The percentage of EDU-positive cells was counted from 500 cells and three independent experiments were performed. Images were captured with a OLYMPUS confocal microscope.

**Plasmids construction**

For Elavl2, Plzf and Dazl expression, plasmid pGMLV-Elavl2, pGMLV-Plzf, pCMV-Myc-Elavl2, pCMV-3×Flag-Dazl were generated by PCR amplification of the coding sequences (CDS) followed by recombination into pGMLV-CMV-MCS-EF1-ZsGreen-T2A-Puro, pCMV-Myc, or pCMV-3×Flag vectors using the ClonExpress One Step Cloning Kit (Vazyme, C112). For knockdown experiment, shRNA-coding DNA fragments were synthesized and cloned into the pGMLV-SC5 vector to create pGMLV-SC5-Elavl2 shRNA plasmid.

**Lentivirus production and transduction**

The pGMLV, pGMLV-Elavl2, pGMLV-SC5, and pGMLV-SC5-Elavl2 shRNA plasmids were cotransfected into HEK-293T cells along with the packaging plasmids pMD2.G and pSPAX2 using lipofectamine 3000 (Invitrogen, USA). Forty-eight hours after cotransfection, virus particles were harvested and filtered by a 0.2 μm cell strainer. The particles were individually used to infect C18-4 and TCam-2 cells with 6 mg/mL polybrene (Sigma, USA). Culture medium was changed to DMEM/F12 with 10% FBS 24 hours later, and green fluorescence was observed under the fluorescent microscope after 72 hours to ensure successful infection. Once green fluorescence was seen, 2 μg/ml puromycin was added into culture medium for screening. About five days later, only C18-4 and TCam-2 cells that were infected successfully survived, which were cultured for further experiments.

**Annexin-V/propidiumiodide (PI) staining and** **flow cytometry**

Cultured stable cell lines were seeded at a density of 1×10^5^ cells/well in 6-well plates in DMEM/F12 supplemented with 10% FBS overnight. 48 hours later, cells were harvested and washed with cold PBS twice, and apoptosis percentages of C18-4 and TCam-2 cells were detected using the Annexin V-FITC/PI kit by flow cytometry according to the manufacturer’s instruction (Biolegend, London, UK). Staining cells simultaneously with Annexin V-FITC (green fluorescence) and the non-vital dye PI (red fluorescence) allowed the discrimination of intact cells (FITC^−^PI^−^), early apoptotic (FITC^+^PI^−^) and late apoptotic or necrotic cells (FITC^+^PI^+^).

**TUNEL Assay**

Further analysis of apoptotic cells was conducted using TUNEL Apoptosis Detection Kit (Yeasen, Shanghai, China). The C18-4 and TCam-2 cells were fixed with 4% PFA for 25 min at 4 ℃. After several washes, the cells were incubated with proteinase K (20 mg/mL) and 1×DNase I buffer for 5 min at room temperature. Cells were then treated with 10 U/mL DNase I for 10 min at room temperature and followed by washes in deionized water. These cells were incubated with 1×equilibration buffer for 30 min at room temperature and labeled by Alexa Fluor in buffer premixed with terminal-deoxynucleoitidyl transferase (TdT) enzyme for 60 min at 37 ℃. After being washed with PBS, the cells were finally stained with DAPI and analyzed under a OLYMPUS confocal microscope.

**IP and co-IP**

Human and mouse testis tissues were lysed in Pierce IP Lysis Buffer (Thermo Scientific, USA) containing complete EDTA-free protease inhibitor cocktail, then immunoprecipitated with anti-ELAVL2 antibody by Pierce Crosslink IP Kit (Thermo Scientific, USA). The co-immunoprecipitated proteins complexes were detected using Western blot. HEK-293T cells transfected with pCMV-Myc, pCMV-Myc-Elavl2, pCMV-3×Flag, or pCMV-3×Flag-Dazl were also lysed with Pierce IP Lysis Buffer, and precipitated with anti-Myc or anti-Flag antibodies in the presence or absence of 5U/μL RNase A (Thermo Scientific, USA), which were detected by Western blot.

**Mass spectrometry**

ELAVL2 protein complex pulled down by anti-ELAVL2 antibody from human and mouse testis tissues were analyzed by mass spectrometry center of ShanghaiTech University using the entire elutes.

**RNA immunoprecipitation (RIP) and sequencing**

RNA immunoprecipitation (RIP) was performed using the EZ-Magna RIP kit (Millipore, Catalog No. 17-701) according to the manufacturer’s instructions. In brief, testes were collected from C57BL/6 mice of 2 weeks old. Testes tissues were lysed with RIP lysis buffer with one freeze-thaw cycle. The lysates were centrifuged at 20,000 g for 15 min, the supernatant was collected and divided into three aliquots, which were then immunoprecipitated with anti-ELAVL2 dynabeads or IgG dynabeads. Input and immunoprecipitated mRNAs were isolated using TRIzol reagent, and were subjected to sequencing (Cloud-Seq Biotech Ltd. Co. Shanghai, China) or real-time qPCR analysis.

**Statistical Analysis**

All data were presented as mean ± SEM. The data obtained in experiments with multiple treatments were subjected to one-way ANOVA followed by Newman-Keuls test of significance using GraphPad software. Student’s *t*-test was employed to study statistical significance in experiments with only two treatments, and *P* < 0.05 was considered statistically significant.
