# Supplementary material for: RNA‐binding protein ELAVL2 plays post‐transcriptional roles in the regulation of spermatogonia proliferation and apoptosis
Source: Cell Prolif. 2021 Jul 23;54(9):e13098. doi: 10.1111/cpr.13098 (PMC8450129; doi:10.1111/cpr.13098)
Supplement: Supplementary file 10 — Appendix S2 [file CPR-54-e13098-s003.docx]

**Supplementary figures and legend**

**RNA-binding protein ELAVL2 plays post-transcriptional roles in the regulation of spermatogonia proliferation and apoptosis**

Chao Yang^1,4^, Chencheng Yao^1,4^, Zhiyong Ji^2,4^, Liangyu Zhao^1^, Huixing Chen^1^, Peng Li^1^, Ruhui Tian^1^, Erlei Zhi^1^, Yuhua Huang^1^, Xia Han^1^, Yan Hong^1^, Zhi Zhou^3^, Zheng Li^1,2^

^1^ Department of Andrology, the Center for Men’s Health, Urologic Medical Center, Shanghai General Hospital, Shanghai Jiao Tong University School of Medicine, Shanghai, China

^2^ State Key Lab of Reproductive Medicine, Nanjing Medical University, Nanjing, China

^3^ School of Life Science and Technology, ShanghaiTech University, Shanghai, China

^4^ These authors contributed equally to this work

**
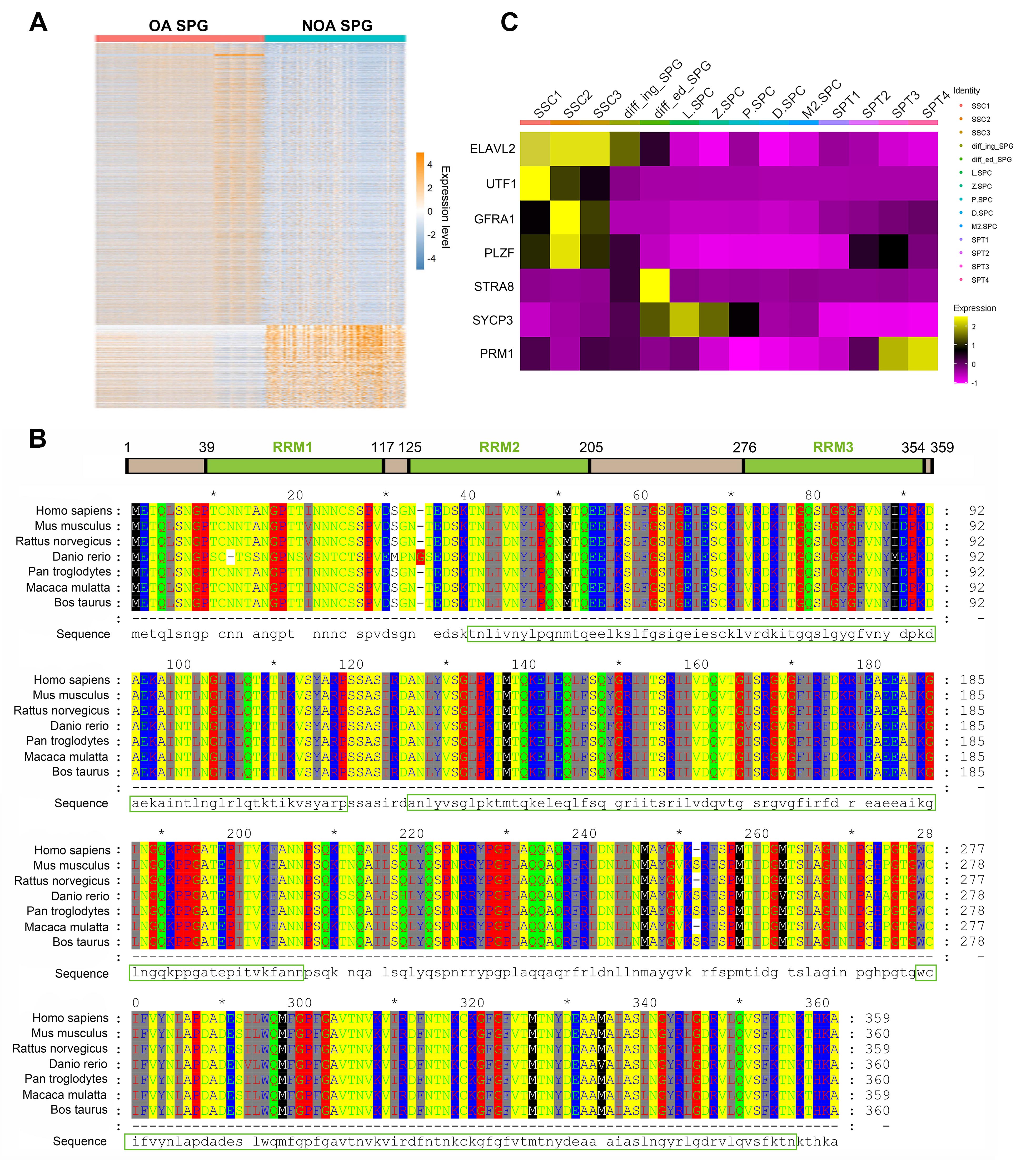
**

**FIGURE S1. Conservation of ELAVL2 protein and single-cell expression of *Elavl2* transcript. A,** Differentially expressed genes between OA and NOA spermatogonia revealed by single-cell sequencing. **B,** Conservation analysis of ELAVL2 protein sequence (upper) across various species. Green boxes highlighted three RNA recognition motifs (RRM1, RRM2, RRM3) of ELAVL2. **C,** Single-cell analysis of *Elavl2* and germ cells markers transcript levels across human spermatogenesis, which was divided into 12 consecutive stages, including 3 stages of SSC (SSC1, SSC2, SSC3). RRM: RNA recognition motif, SSC: spermatogonia stem cell, diff_ing SPG: differentiating spermatogonia, diff_ed SPG: differentiated spermatogonia, L: leptotene, Z: zygotene, P: pachytene, D: diplotene, M: meiosis II spermatocytes, SPT: spermatid.


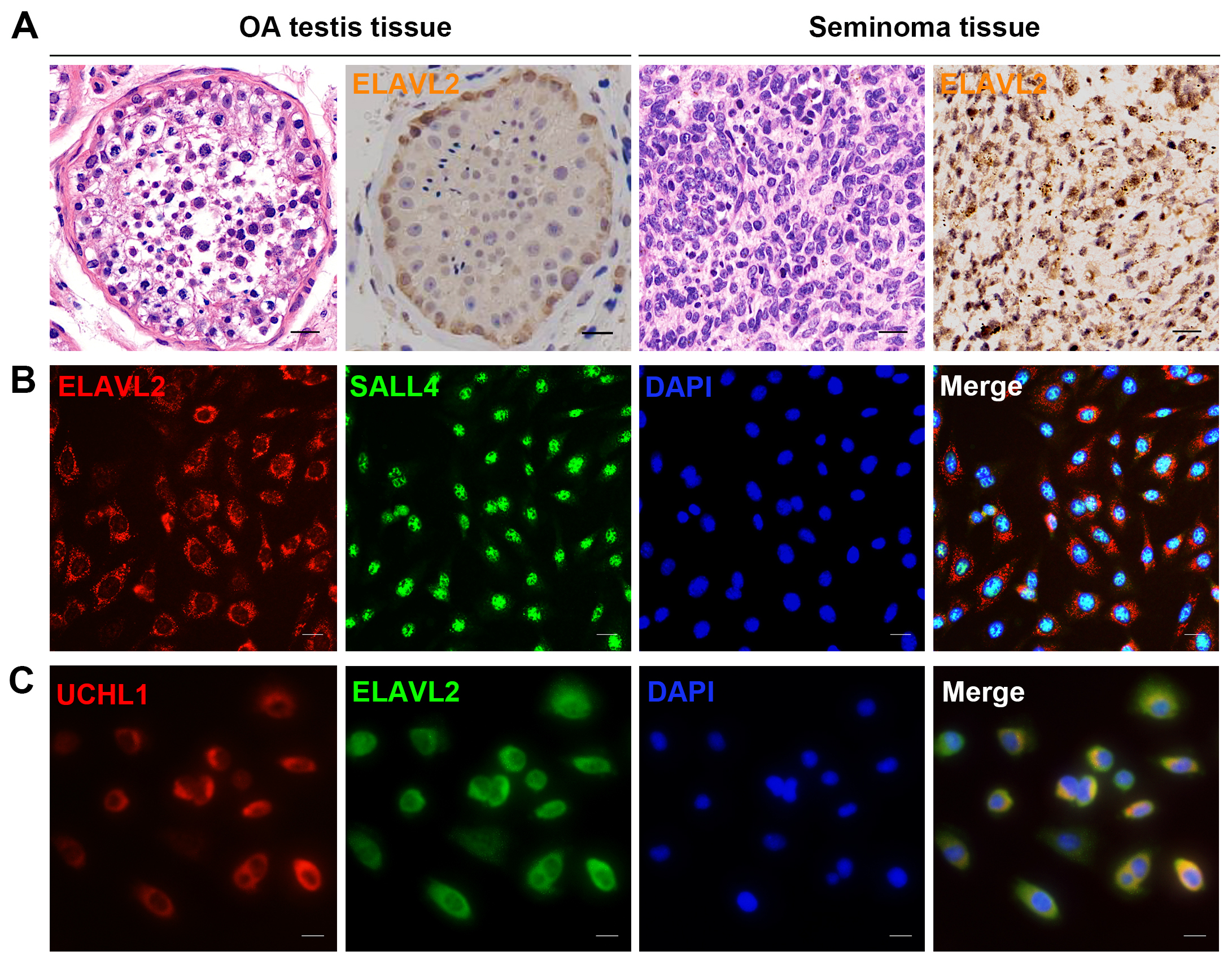


**FIGURE S2. Expression of ELAVL2 in OA and seminoma tissues and cell lines. A,** HE staining and immunohistochemical staining of ELAVL2 in OA (left panel) and seminoma (right panel) tissues. Scale bar: 20 µm. **B,** Co-staining of ELAVL2 with SSCs marker SALL4 in TCam-2 cell line derived from pure seminoma. Scale bar: 20 µm. **C,** Co-staining of ELAVL2 with spermatogonia marker UCHL1 in mouse SSCs cell line C18-4. Cell nuclei were stained with DAPI. Scale bar: 20 µm.


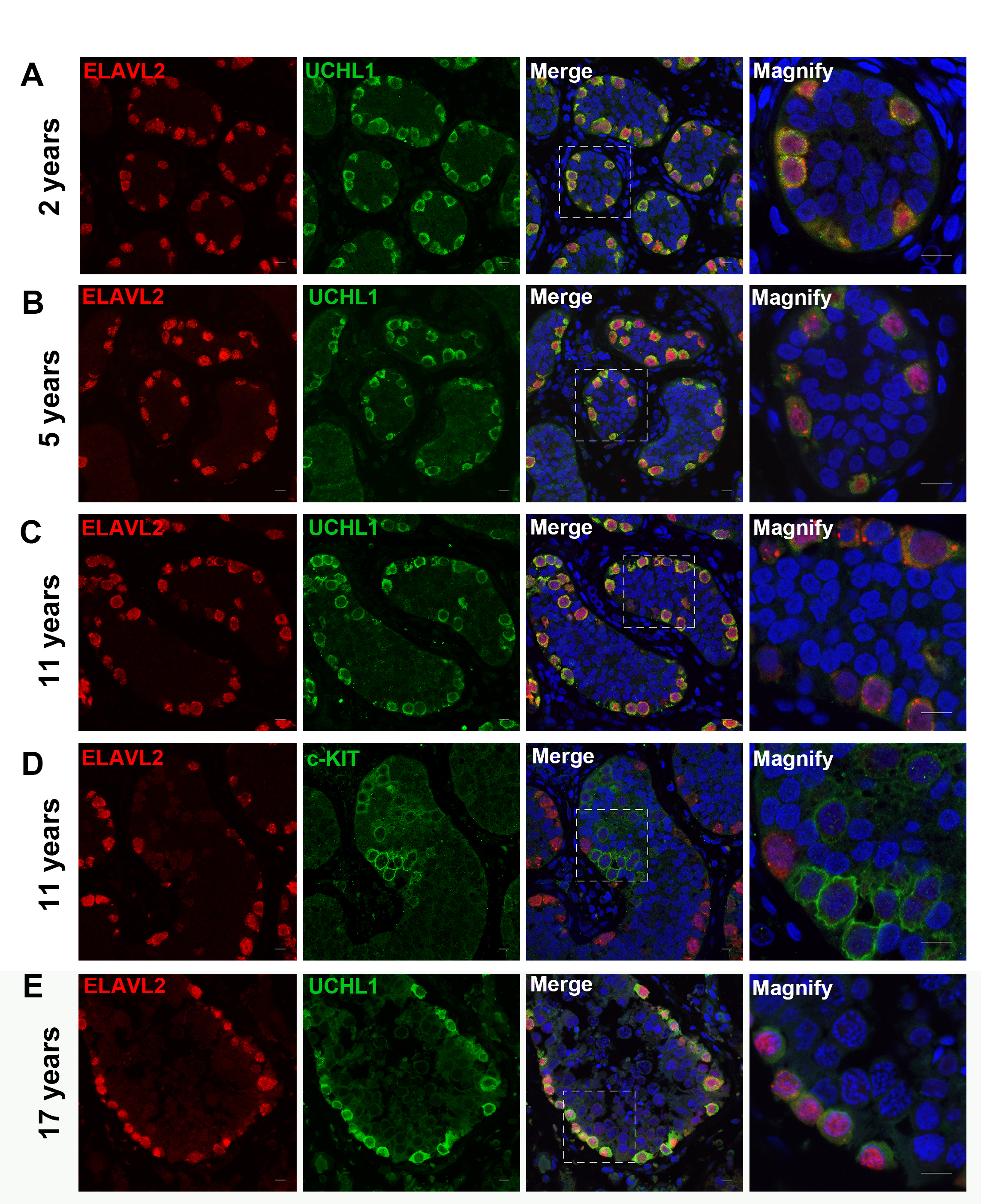


**FIGURE S3. ELAVL2 is enriched in human SSCs in testes of different ages. A, B, C, E,** Co-staining of ELAVL2 with spermatogonia marker UCHL1 in human testis tissues of different ages, including 2-year-old (A), 5-year-old (B), 11-year-old (C), and 17-year-old (E). **D,** Co-staining of ELAVL2 with differentiating spermatogonia marker c-KIT in 11-year-old human testis tissue. Scale bar: 20 µm.


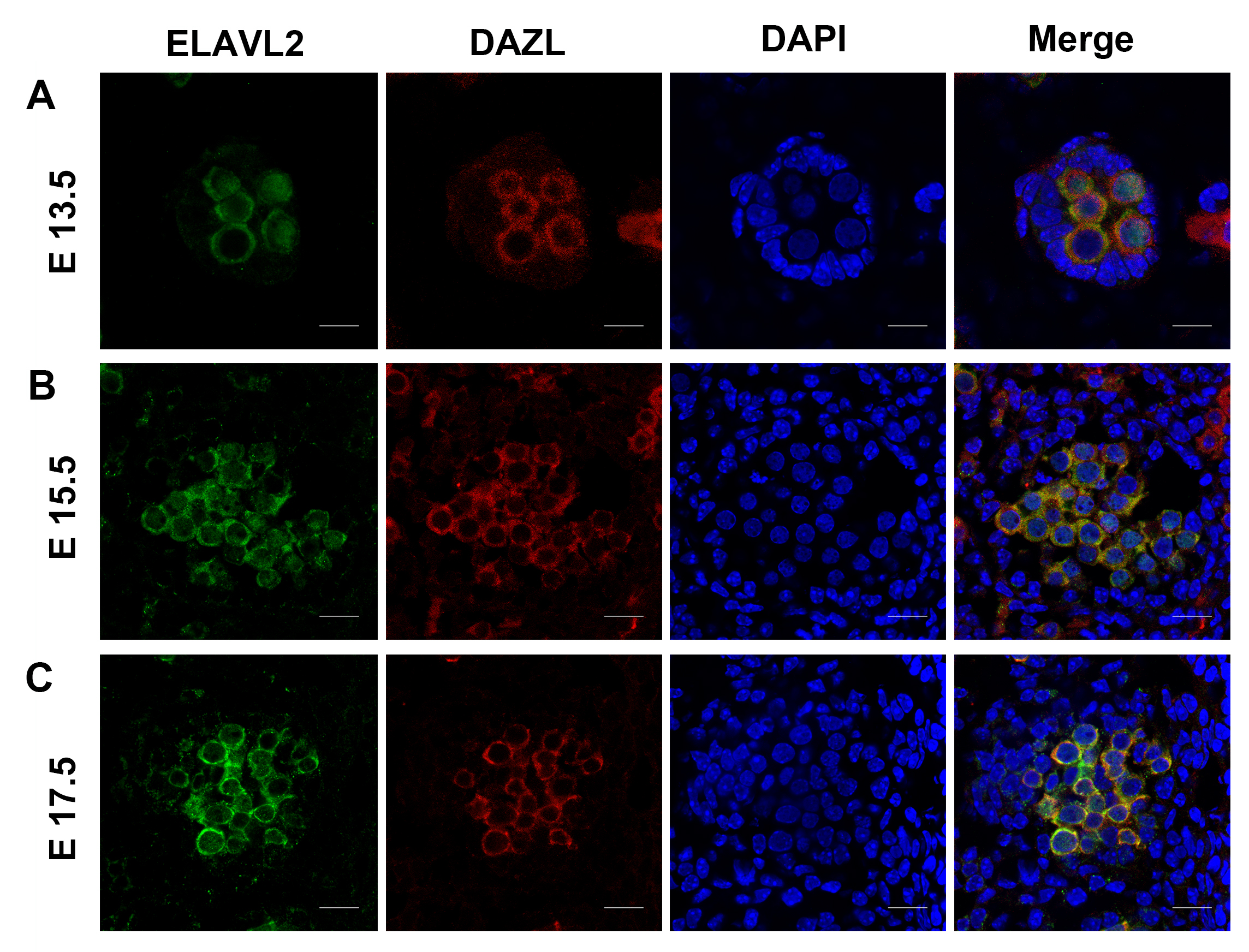


**FIGURE S4.** **ELAVL2 is enriched in mouse gonocytes in embryonic testes at different time points. A, B, C,** Co-expression of ELAVL2 and DAZL in mouse embryonic testes at E13.5 (A), E15.5 (B), and E17.5 (C). Scale bar in A: 10 µm, Scale bar in B and C: 20 µm.


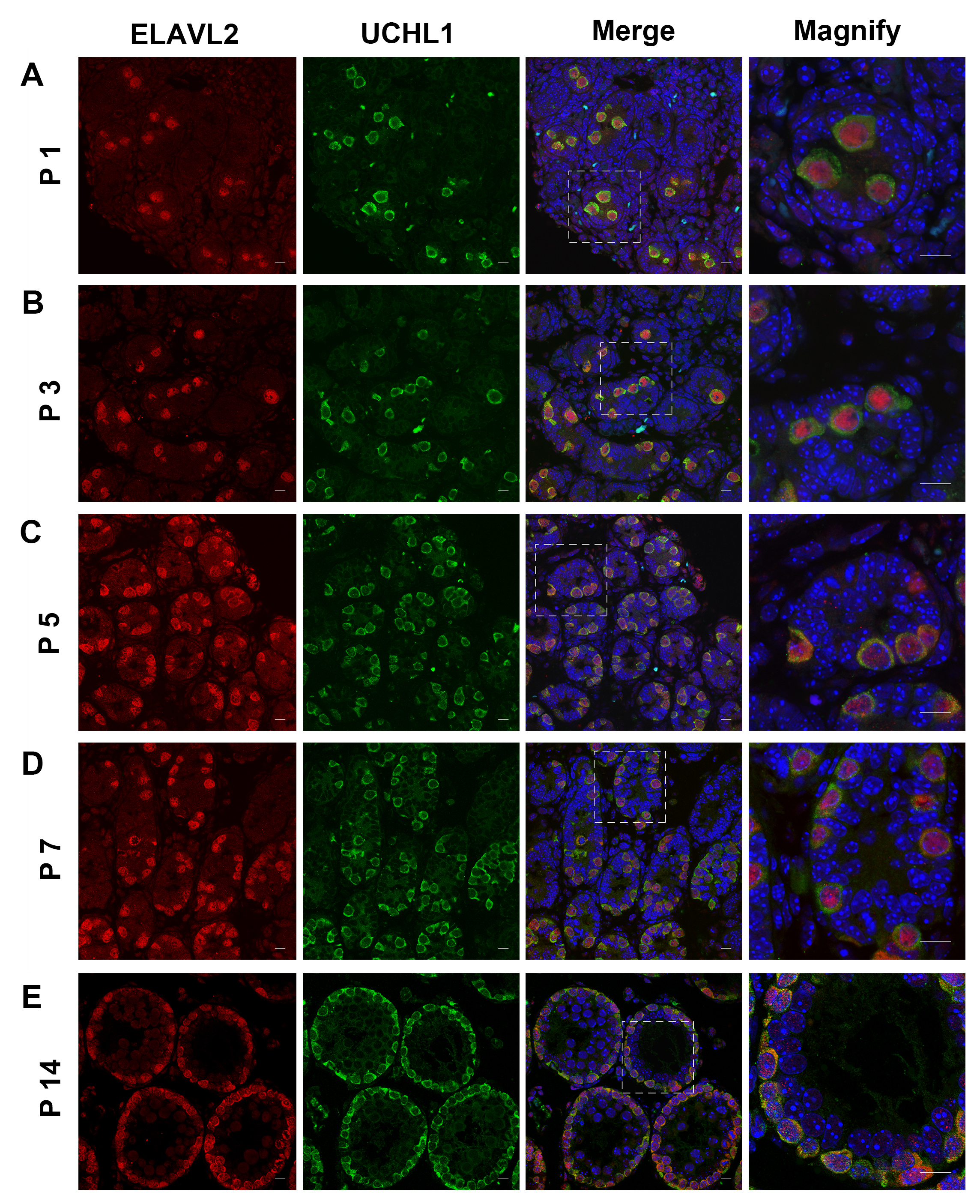


**FIGURE S5. ELAVL2 is enriched in mouse gonocytes and SSCs in postnatal testes at different time points. A-E,** Co-staining of ELAVL2 with spermatogonia marker UCHL1 in postnatal testes at different time points, including P1 (A), P3 (B), P5 (C), P7 (D), and P14 (E). Scale bar: 20 µm.


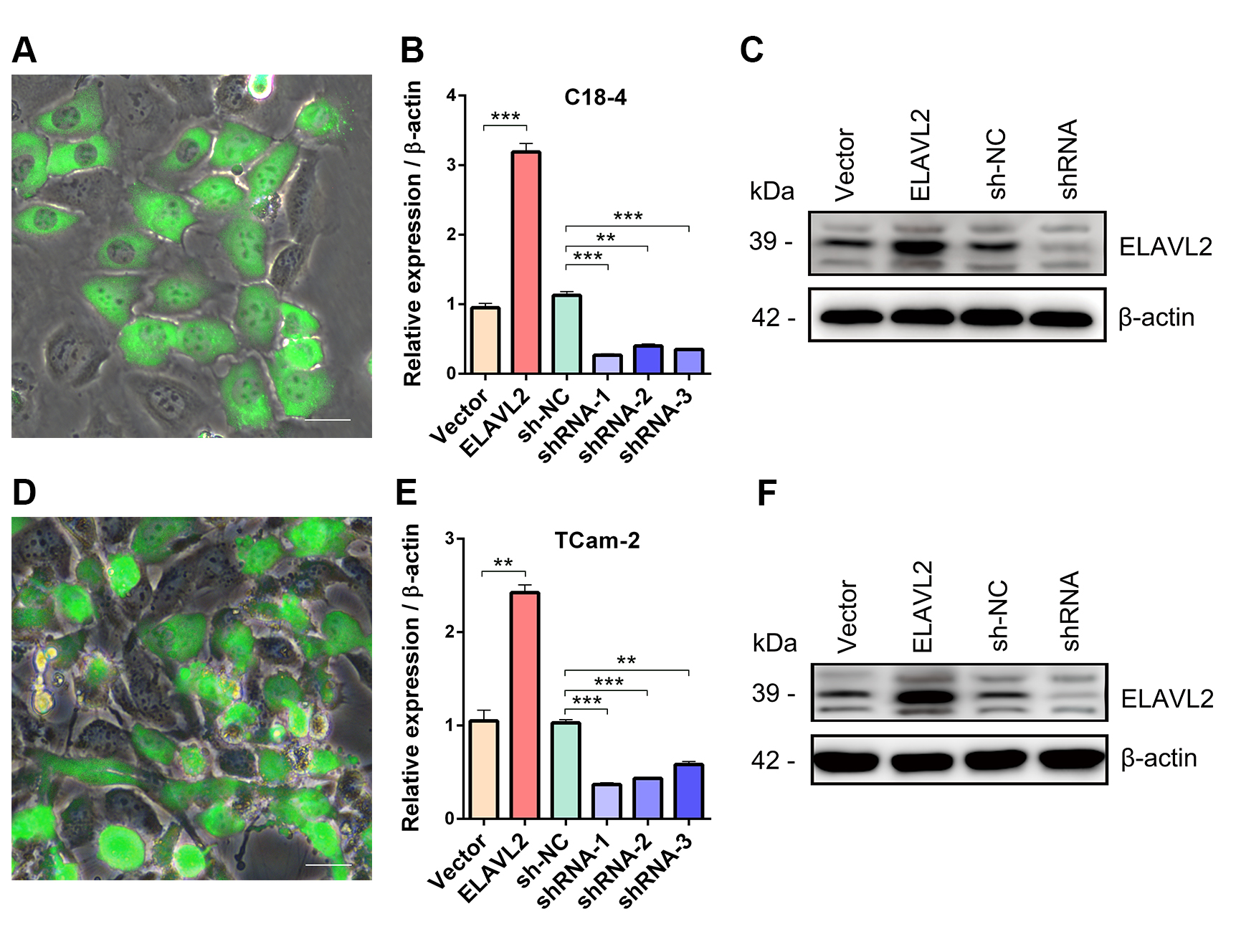


**FIGURE S6. Establishment of stable C18-4 and TCam-2 cell lines with up- or down-regulated ELAVL2 via lentivirus infection. A, D,** EGFP fluorescence after infection and puromycin screening in C18-4 (A) and TCam-2 cell lines (D). Scale bars: 20 μm. **B, E,** Different *Elavl2* mRNA expression revealed by qPCR in C18-4 (B) and TCam-2 cell lines (E) after infection. β-actin served as internal control. shRNA-1 was most efficient and used for further study. ***P* < 0.01, ****P* < 0.001. **C, F,** Different ELAVL2 protein expression revealed by Western blot in C18-4 (C) and TCam-2 cell lines (F) after infection. β-actin served as internal control. All error bars show SEM.





**FIGURE S7. Effects of AKT and ERK inhibitors on the proliferation and downstream gene expressions of C18-4 cells. A,** EDU staining in C18-4 cell lines with or without ELAVL2 overexpression and AKT inhibitor (GSK690693) or ERK inhibitor (SCH772984) treatment. Cell nuclei were stained with DAPI. Scale bar: 20 µm. ***P* < 0.01, ****P* < 0.001. **B,** Protein expressions of AKT and ERK downstream genes in C18-4 cell lines with or without ELAVL2 overexpression and AKT inhibitor (GSK690693) or ERK inhibitor (SCH772984) treatment. ns: no significant difference, **P* < 0.05, ***P* < 0.01, ****P* < 0.001. All error bars show SEM.


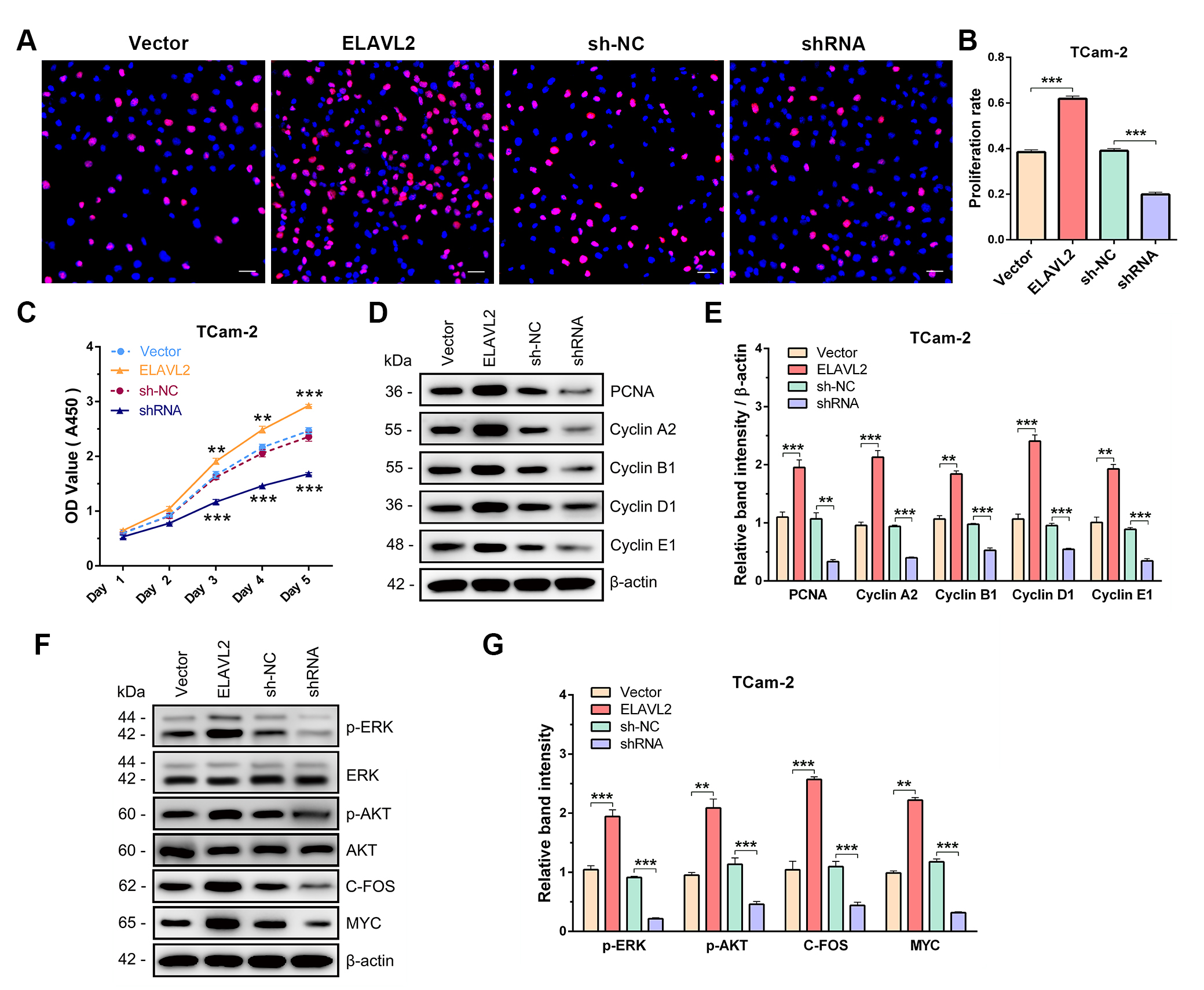


**FIGURE S8. ELAVL2 promotes the proliferation of TCam-2 cells. A,** EDU staining in stable cell lines with up- or down-regulated ELAVL2. Cell nuclei were stained with DAPI. Scale bar: 20 µm. **B,** The EDU positive rates of the four stable cell lines. ****P* < 0.001. **C,** The growth curve of the four stable cell lines for 5 days revealed by CCK-8 assay. ***P* < 0.01, ****P* < 0.001. **D, E,** The protein expressions of PCNA and cell-cycle proteins (Cyclin A2, Cyclin B1, Cyclin D1, and Cyclin E1) in the stable cell lines (D). The band intensity was normalized to β-actin (E). ***P* < 0.01, ****P* < 0.001. **F, G,** The protein expressions of p-ERK, ERK, p-AKT, AKT, C-FOS, and MYC in the stable cell lines (F). The band intensity of p-ERK and p-AKT were normalized to total ERK and AKT respectively, the band intensity of C-FOS and MYC were normalized to β-actin (G). ***P* < 0.01, ****P* < 0.001. All error bars show SEM.


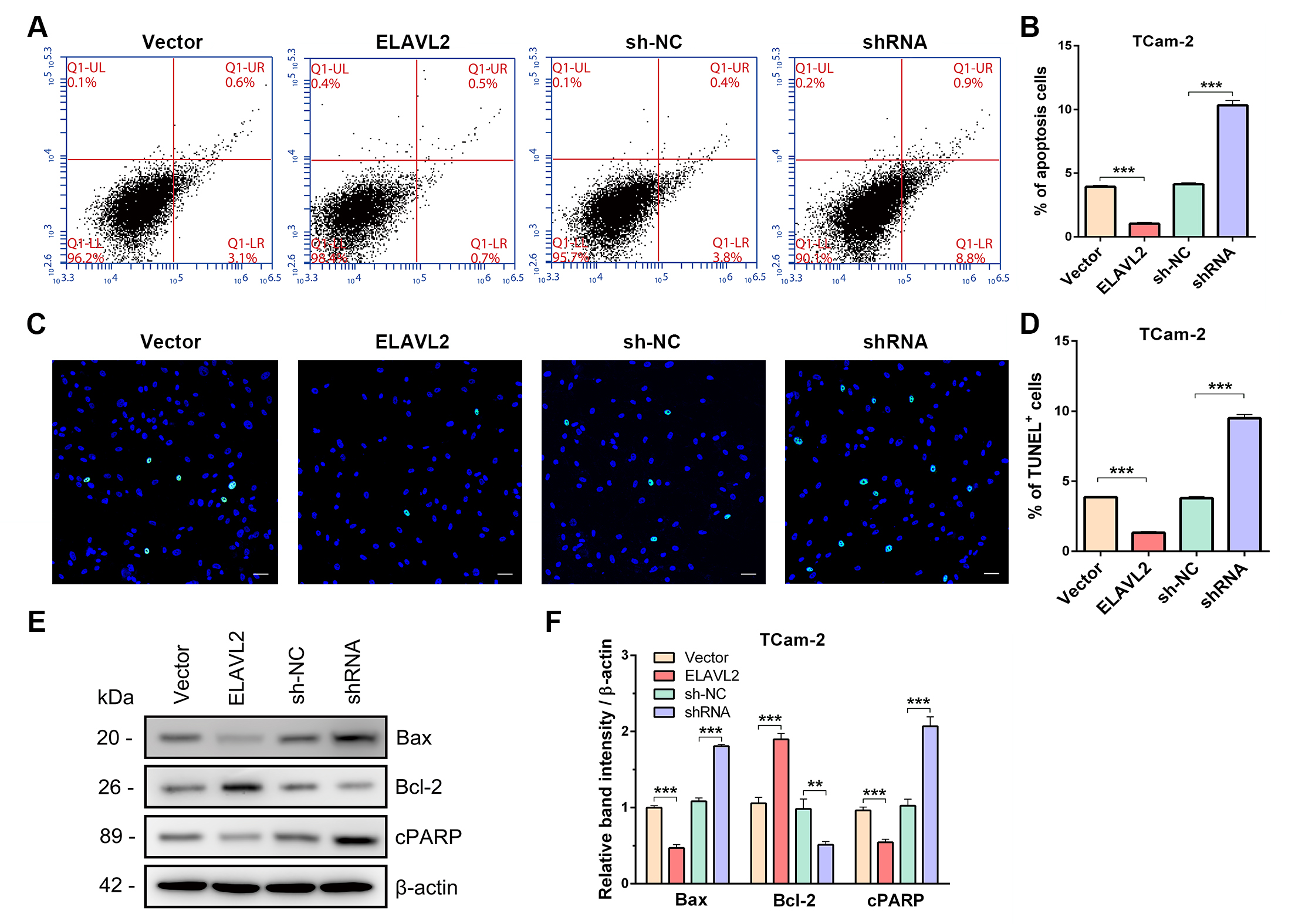


**FIGURE S9. ELAVL2 inhibits the apoptosis of** **TCam-2 cells. A, B,** Annexin V-APC/PI and flow cytometry analysis of TCam-2 cells apoptosis. A total of 10,000 cells were analyzed. ****P* < 0.001. **C, D,** TUNEL assay of apoptotic cells in the four stable TCam-2 cell lines. Scale bar: 20 µm. ****P* < 0.001. **E, F,** The protein expression of Bax, Bcl-2, and cPARP in the stable cell lines (E). The band intensity was normalized to β-actin (F). ***P* < 0.01, ****P* < 0.001. All error bars show SEM.


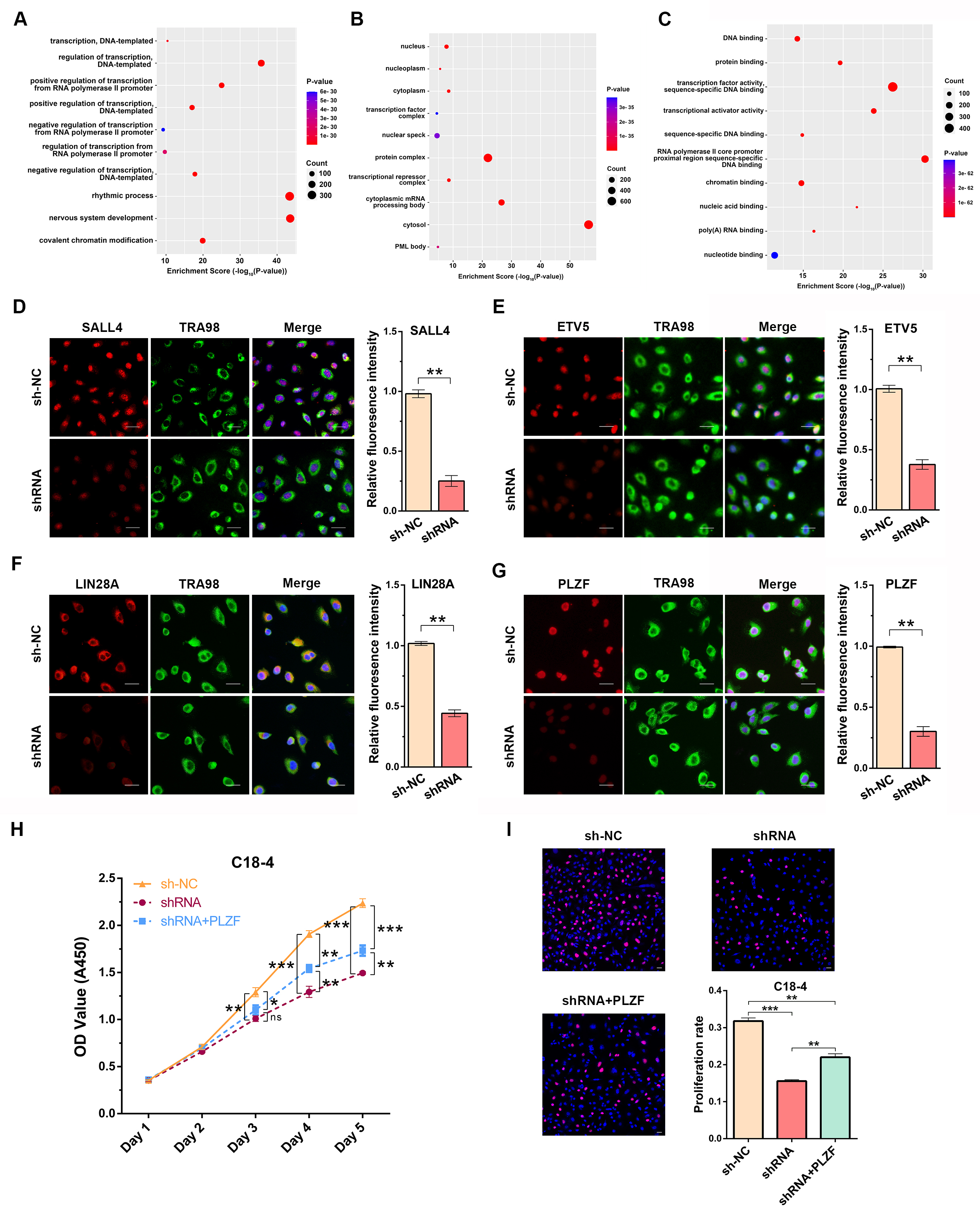


**FIGURE S10. GO analysis and protein expression of ELAVL2 targets. A-C,** Gene Ontology (GO) analysis of target mRNAs precipitated by anti-ELAVL2 antibody in mouse testis, including biological process (A), cellular component (B), and molecular function (C). **D-G,** Immunostaining of ELAVL2 targets in C18-4 cells was compared between control group and shRNA group with ELAVL2 knockdown, including SALL4 (D), ETV5 (E), LIN28A (F), and PLZF (G). Fluorescence intensity was normalized to germ cells marker TRA98. **H,** The growth curve of the C18-4 cell lines with or without PLZF overexpression for 5 days revealed by CCK-8 assay. ns: no significant difference, **P* < 0.05, ***P* < 0.01, ****P* < 0.001. **I,** EDU staining in C18-4 cell lines with or without PLZF overexpression. Cell nuclei were stained with DAPI. ** *P* < 0.01, ****P* < 0.001. Scale bar: 20 µm. All error bars show SEM.


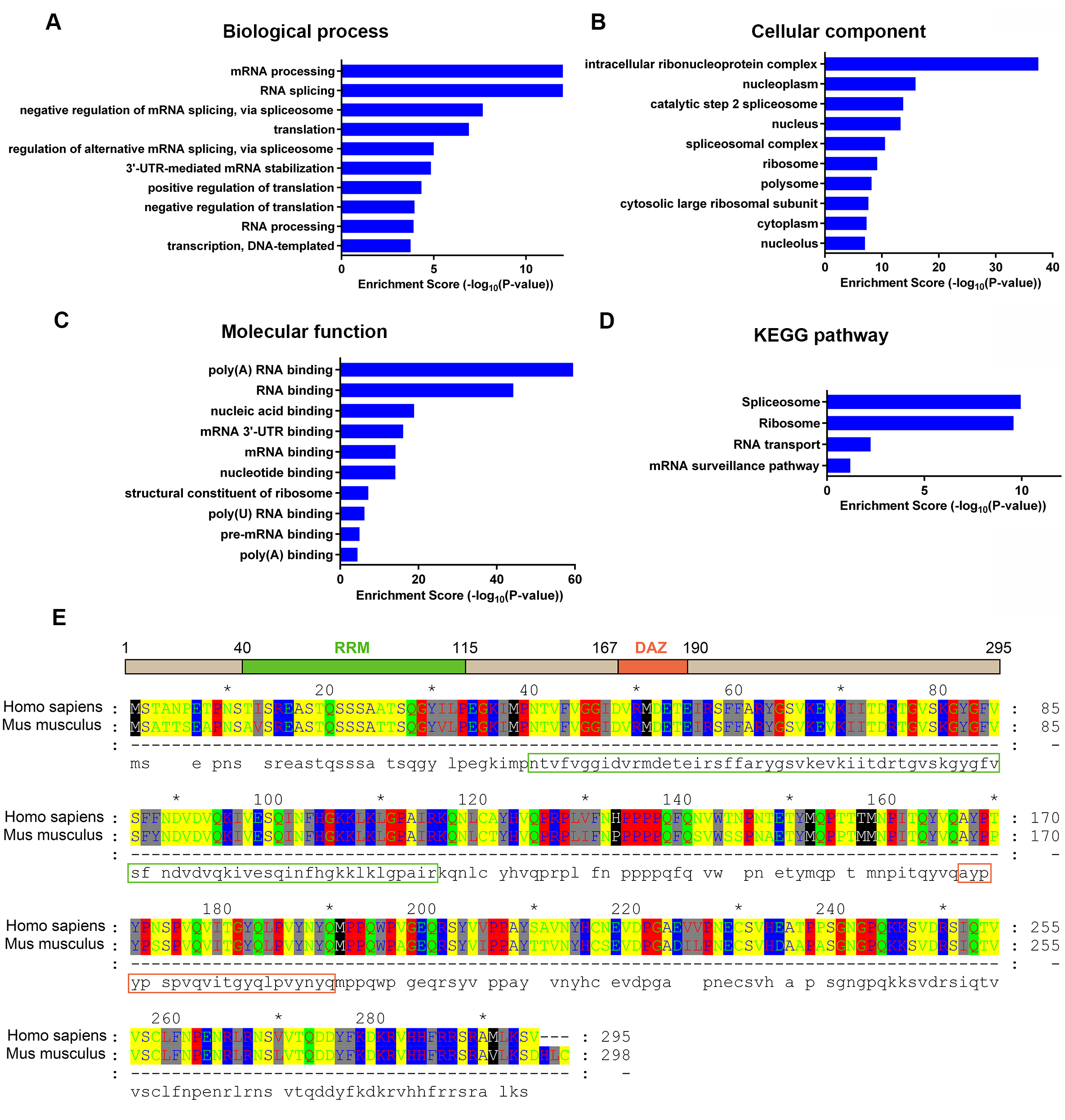


**FIGURE S11. GO and KEGG pathway enrichment of proteins associate with ELAVL2 and conservation analysis of DAZL. A, B, C,** Gene Ontology (GO) analysis of proteins associate with ELAVL2 in both human and mouse testes, including biological process (A), cellular component (B), and molecular function (C). **D,** KEGG pathway analysis of proteins associate with ELAVL2 in both human and mouse testes. **E,** Conservation analysis of DAZL protein sequence (upper) between human and mouse. Green box highlighted the RNA recognition motif (RRM), and orange box highlighted the deleted in azoospermia motif (DAZ) of DAZL.
