# Supplementary material for: RNA‐binding protein ELAVL2 plays post‐transcriptional roles in the regulation of spermatogonia proliferation and apoptosis
Source: Cell Prolif. 2021 Jul 23;54(9):e13098. doi: 10.1111/cpr.13098 (PMC8450129; doi:10.1111/cpr.13098)
Supplement: Supplementary file 11 — Appendix S3 [file CPR-54-e13098-s011.docx]

**Informed consents and IRB paperwork**

**RNA-binding protein ELAVL2 plays post-transcriptional roles in the regulation of spermatogonia proliferation and apoptosis**

**Running title: Regulatory functions of ELAVL2 in spermatogonia**

Chao Yang^1,4^, Chencheng Yao^1,4^, Zhiyong Ji^2,4^, Liangyu Zhao^1^, Huixing Chen^1^, Peng Li^1^, Ruhui Tian^1^, Erlei Zhi^1^, Yuhua Huang^1^, Xia Han^1^, Yan Hong^1^, Zhi Zhou^3^, Zheng Li^1,2^

**
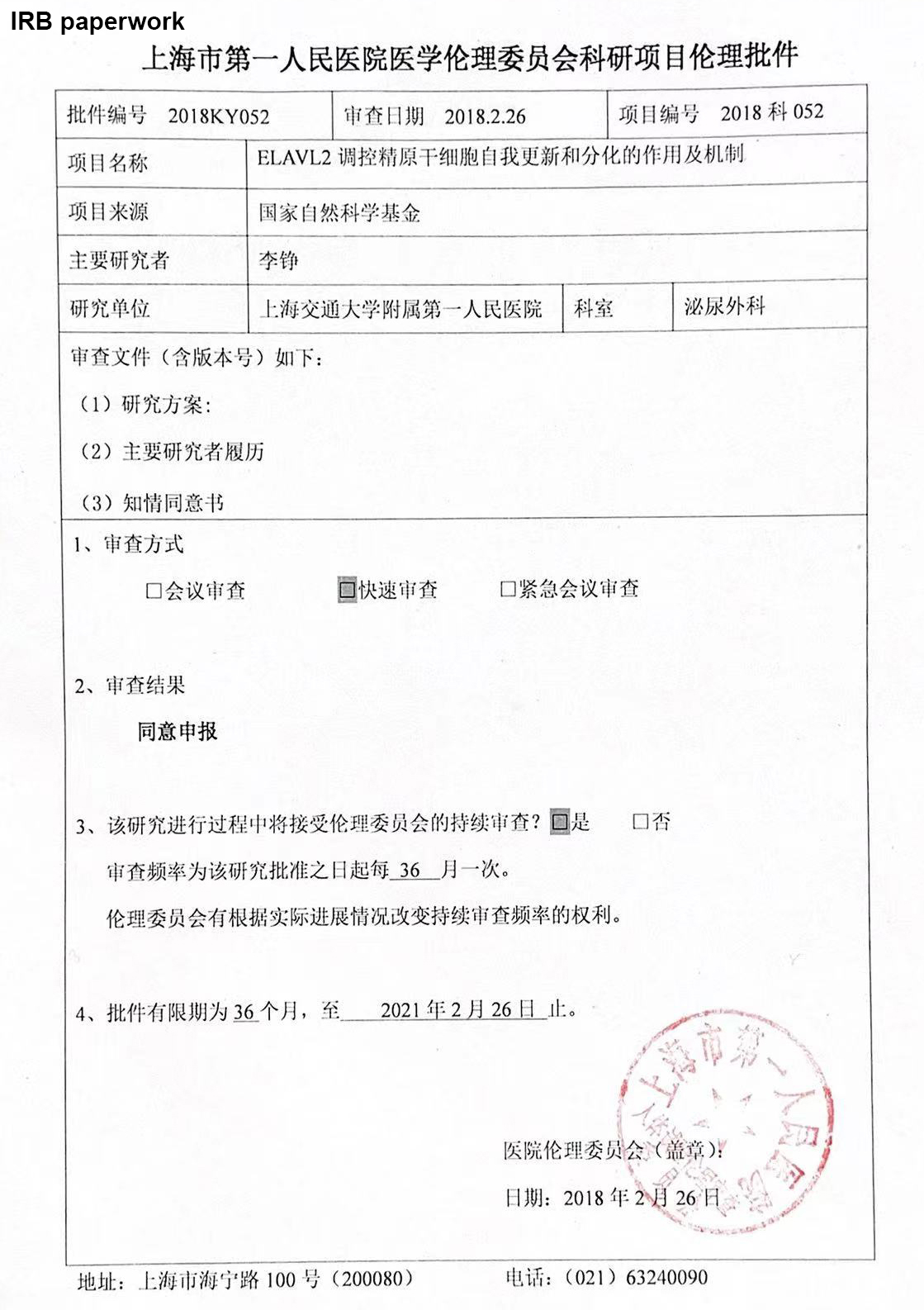
**

**IRB paperwork in English**

**Approved ethical license for research project of Shanghai General Hospital**

| **License No.** 2018KY052 | **Date**  2018.2.26 | **Research No.**  2018科052 | |
| --- | --- | --- | --- |
| **Research Title** | The Function and Mechanism of ELAVL2 in Regulation of Spermatogonial Stem Cell Self-renewal and Differentiation | | |
| **Funding** | National Natural Science Foundation of China | | |
| **Principal Investigator** | Li Zheng | | |
| **Institution** | Shanghai General Hospital | **Department** | Urologic Medical Center |
| **Documents for ethical review (with version number):**  (1) research approach  (2) curriculum vitae of the principal investigator  (3) informed consents | | | |
| 1. Ethical review method  □ Review meeting □ Rapid review □ Urgent review meeting  2. Ethical review result  Approved  3. Will the research be supervised continuously by the ethics committee? □ Yes □ No  The frequency of ethical review will be once every 36 months.  The ethics committee has the right to adjust the frequency of ethical review according to the progress of the research.  4. The expiration date of this approved license is 36 months, till 2021.2.26 .  Ethics committee of hospital (stamp)  Date: 2018.2.26 | | | |

Address: 100 Haining Road, Shanghai Tel: (021) 63240090

**Informed consent for surgery in Chinese (2-years-old child)**

**
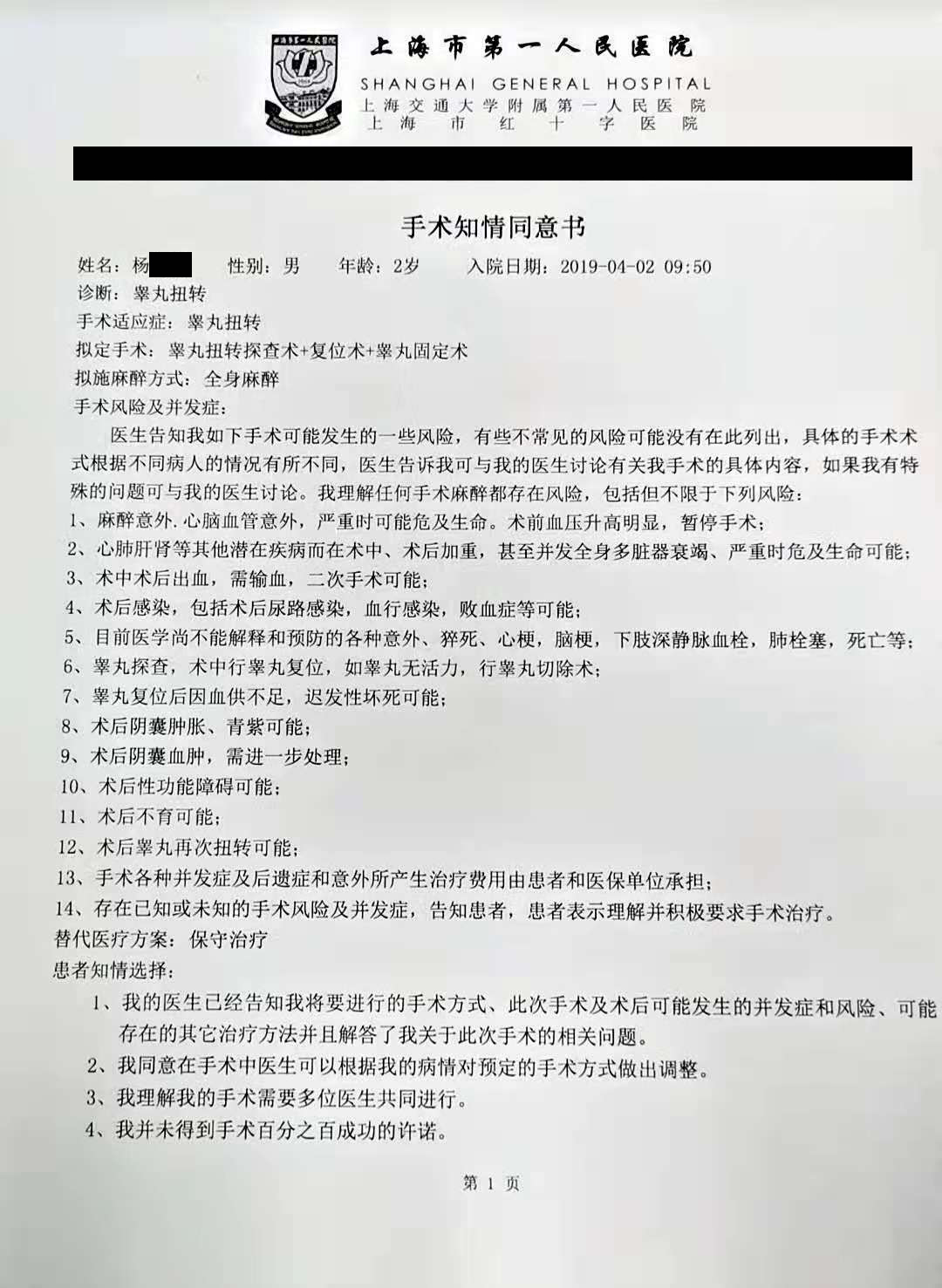

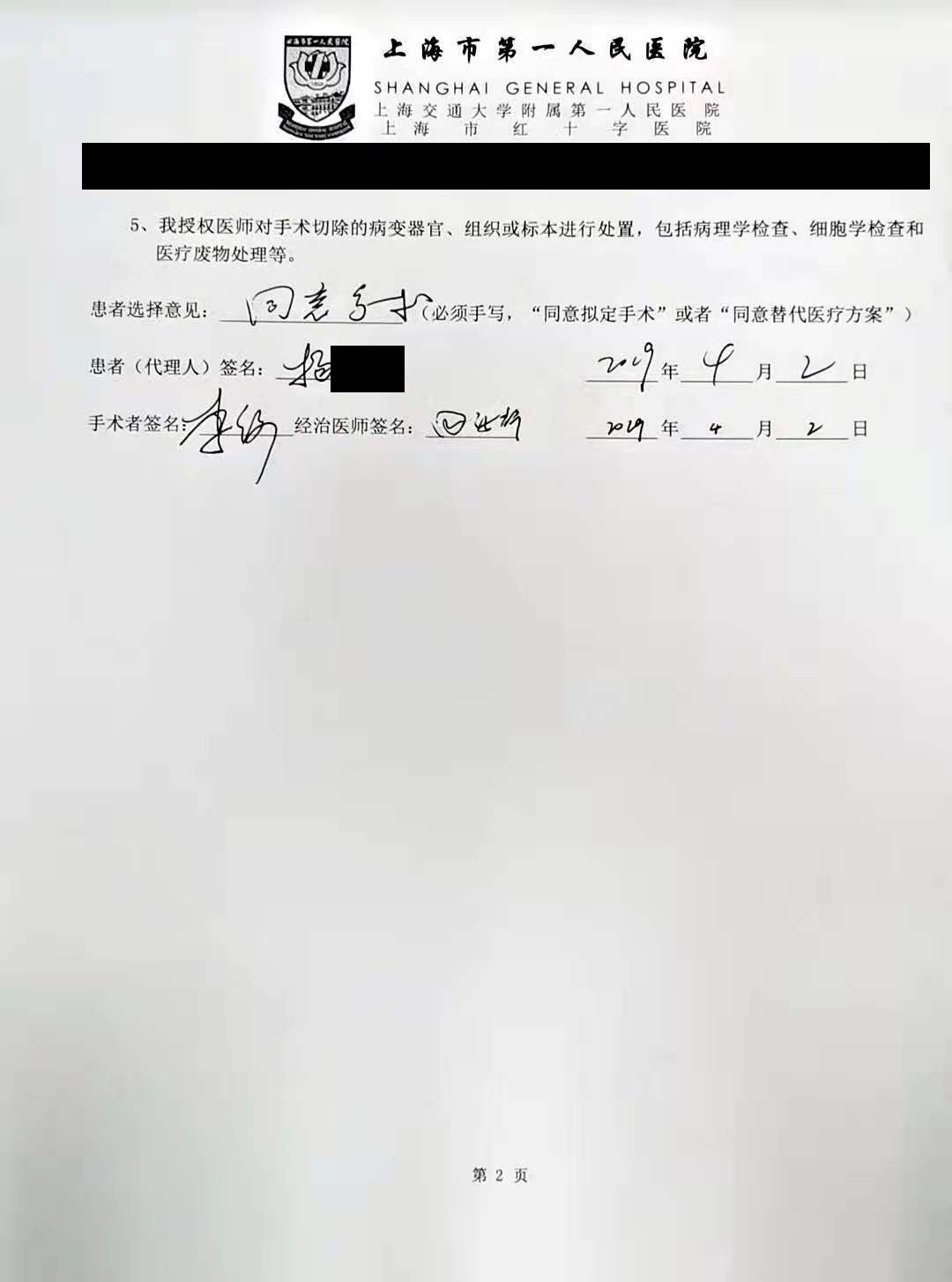
**

**Informed consent for surgery in English (2-years-old child)**

**Name:** **Gender:** male **Age:** 2 years old

**Date of Admission:** 2019-04-02 09:50

**Diagnosis:** Testicular torsion

**Surgical indication:** Testicular torsion

**Proposed surgery:** Exploration of testicular torsion, testicular detorsion, orchidopexy

**Method of anesthesia:** General anesthesia operation

**Surgical risk and complications:**

The doctor informed me of the possible risks of the following surgery. Some uncommon risks may not be listed here. The specific surgical procedures vary according to the conditions of different patients. The doctor told me that I can discuss with my doctor about my surgery if I have specific problems. I understand that any surgical anesthesia has risks, including but not limited to the following risks:

1. Anesthesia accident; cardiovascular and cerebrovascular accident, which may be life-threatening in serious cases; the blood pressure increased significantly before operation and the operation may be suspended;

2. Other potential diseases of lung, liver and kidney, may aggravate during and after operation, or even complicated with systemic multiple organ failure, which may be life-threatening in serious cases;

3. Intraoperative and postoperative bleeding; the need of blood transfusion; and secondary operation may cause complications;

4. Postoperative infection, including postoperative urinary tract infection, hematogenous infection, sepsis, etc;

5. All kinds of accidents that currently cannot be explained and prevented by medicine, like myocardial infarction, cerebral infarction, lower extremity deep vein thrombosis, pulmonary embolism, death, etc;

6. The whole or a part of testis could be excised if it presents necrosis;

7. Delayed necrosis due to insufficient blood supply after recovery of testicular torsion;

8. Postoperative scrotal swelling and cyanosis;

9. Postoperative scrotal hematoma and the need for further treatment;

10. Sexual dysfunction may occur after operation;

11. Infertility may occur after operation;

12. The possibility of testicular torsion after operation;

13. The treatment cost of complications, sequelae and accidents is borne by patients and medical insurance institutions;

14. The patient has been informed of the known and unknown surgical risks and complications, and the patient understands and actively requests for surgical treatment.

**Alternative medical treatment:** conservative treatment

**Patient’s choice:**

1. My doctor has informed me of the operation to be performed, the possible complications and risks of this operation, other possible treatment methods, and has answered my questions about the operation.

2. I agree that the doctor can adjust the scheduled operation method according to my condition during the operation.

3. I understand that my surgery needs to be performed by multiple doctors.

4. I am not promised of certain success of the operation.

5. I authorize doctors to dispose the impaired organs, tissues or specimens removed by surgery, including pathological examinations, cytological examinations, and medical waste disposal.

**Patient’s choice:** (Must be handwritten, "Agree to the proposed surgery" or "Agree to an alternative medical plan)

**Patient (****agent) signature: Date:**

**Signature of surgeon: Signature of attending doctor: Date:**

**Informed consent for research in Chinese (2-years-old child)**


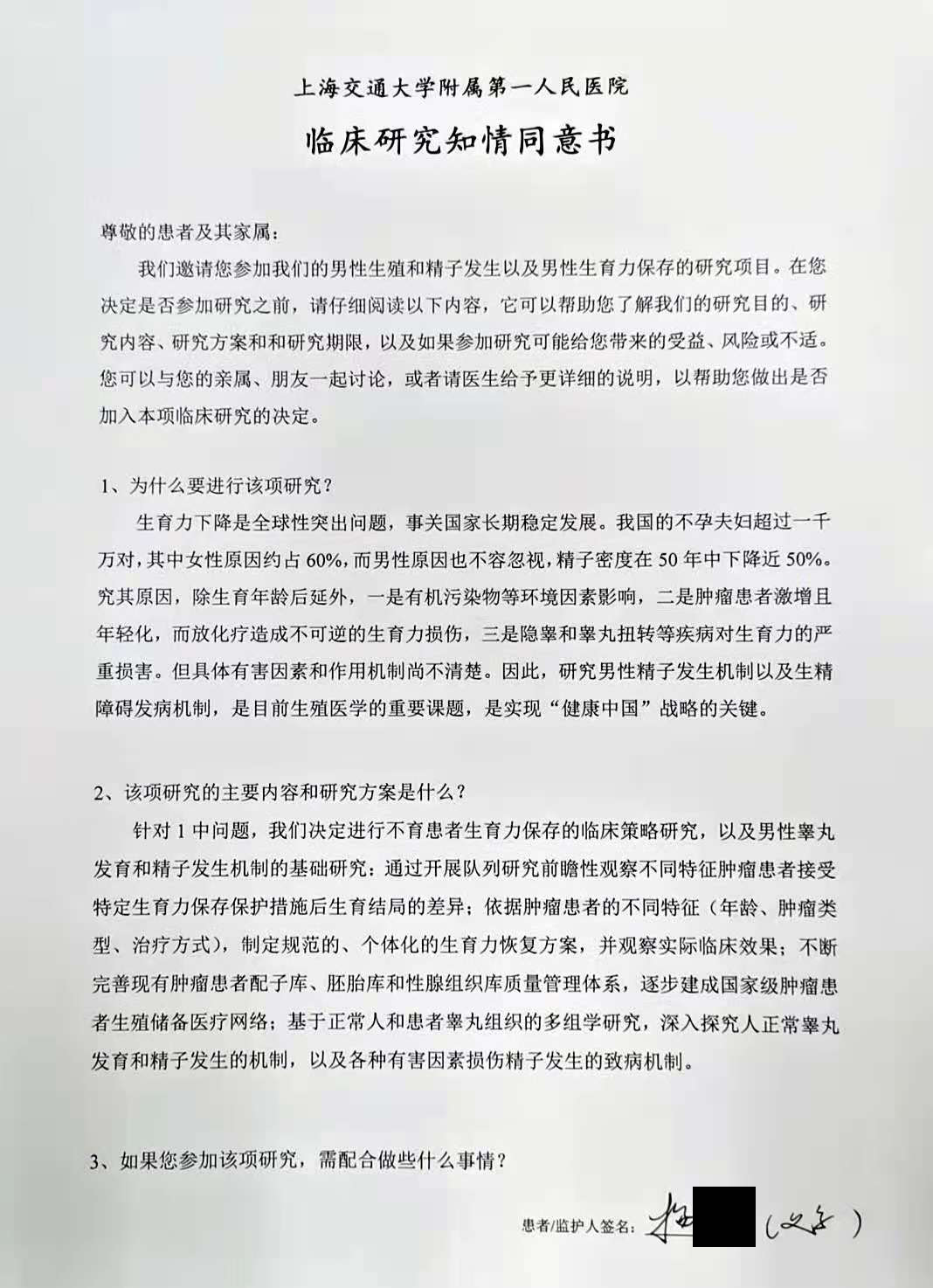

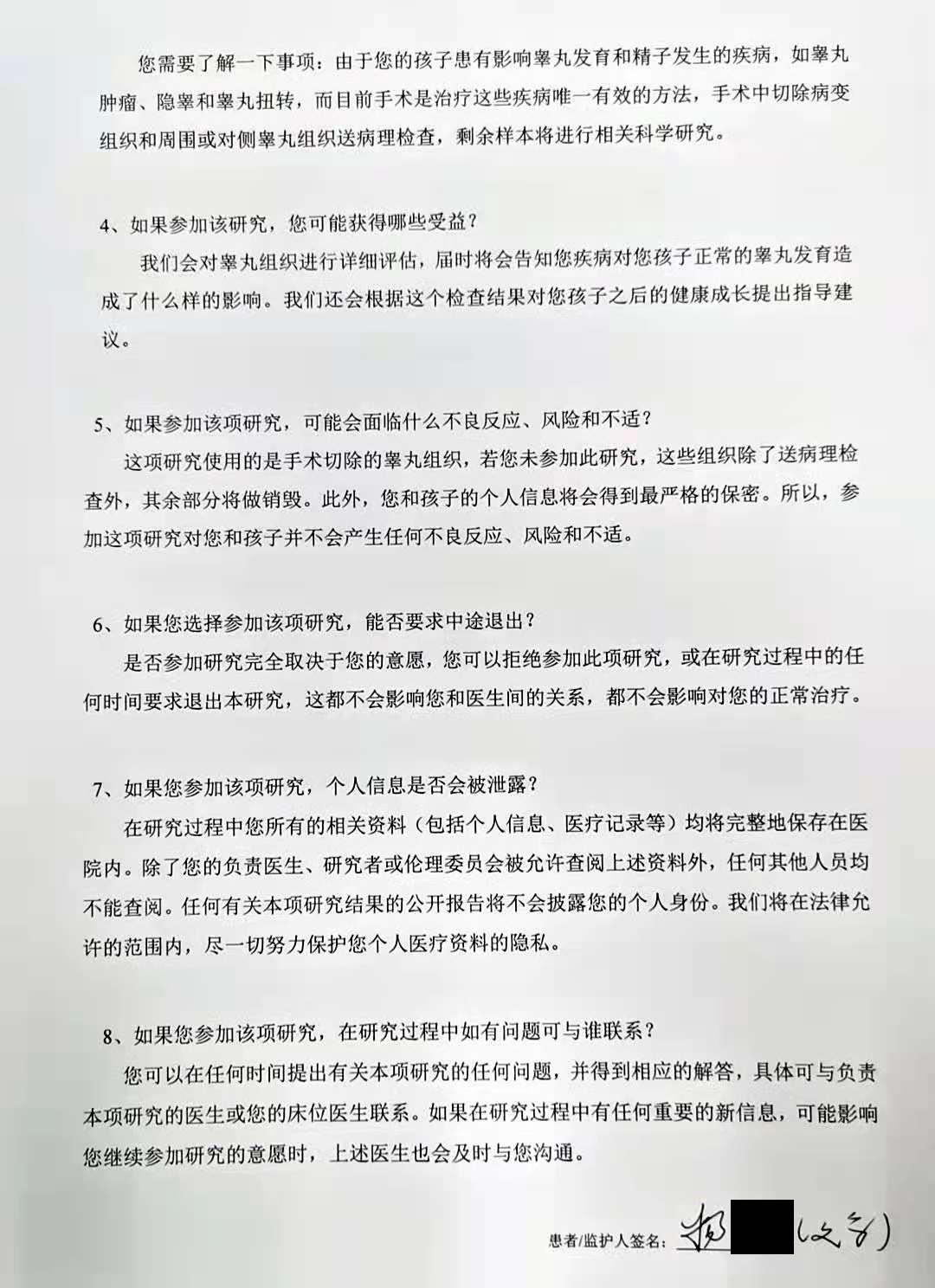

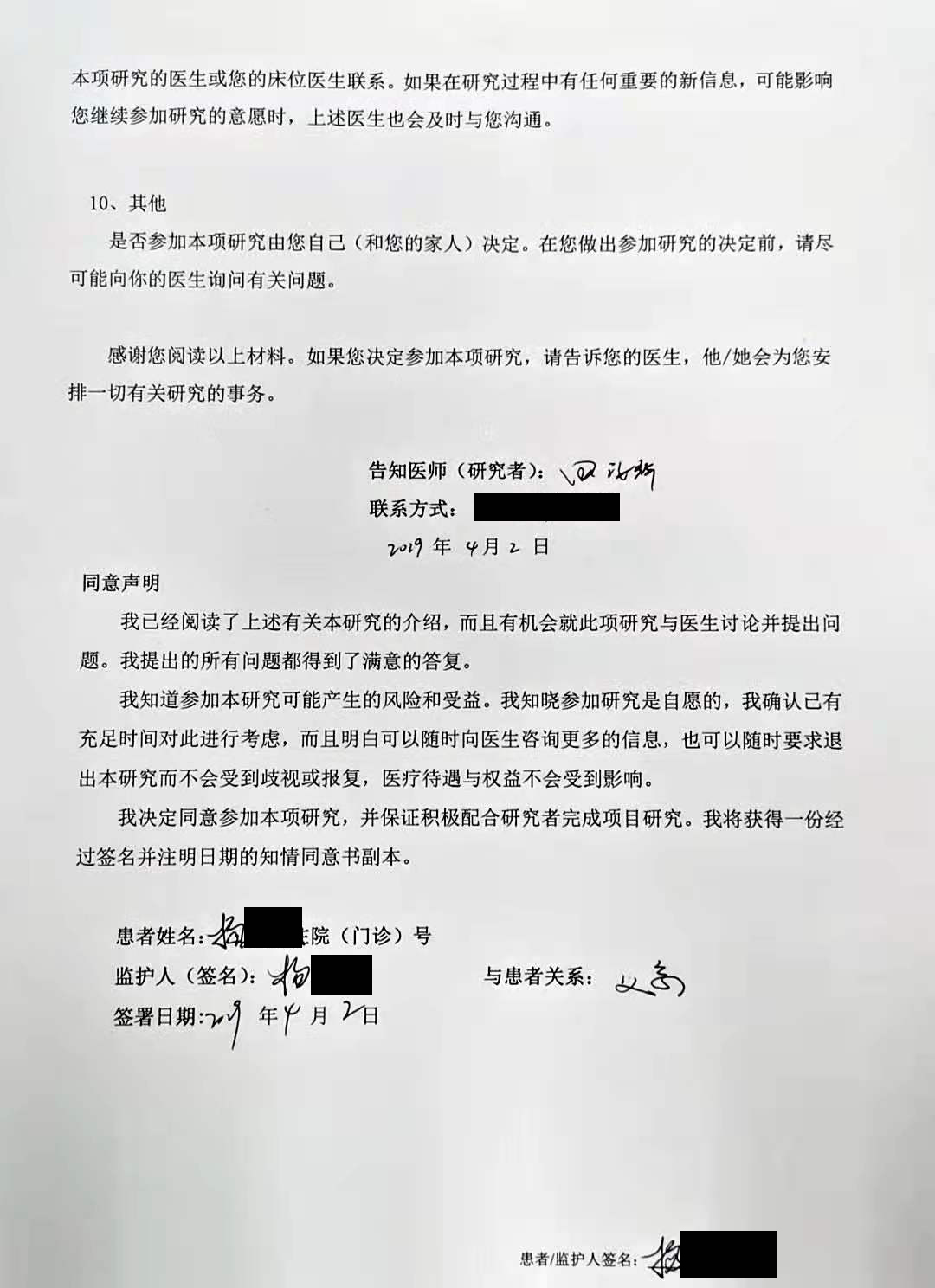


**Informed consent for research in English (for all patients)**

Dear patients and relatives:

We sincerely invite you to participate in our research project on male reproduction, spermatogenesis and male fertility preservation. Before you make decisions, please carefully read the following information, which describes all aspects of the research, including research purpose, research approach, benefits for participants and potential risks. You could discuss with your family members and friends, or ask us for more detailed information, before you make your minds.

**1. Why we conduct this research?**

Declined fertility is a severe issue worldwide and is closely associated with the long-run and steady development of our country. In China, over 10 million couples are infertile, and female factors account for nearly 60% of all cases. Male infertility is also severe and should not be ignored. Actually, sperm concentration has declined about 50% in recent 50 years. Many factors attribute to this, including delay of childbearing age, environmental factors like organic pollutant, impairment of fertility by chemotherapy and radiotherapy for cancer patients, and diseases of male reproductive system like cryptorchidism and testicular torsion. However, the mechanisms of these factors are mostly elusive and unclear. Therefore, it is imperative to study the mechanisms of normal spermatogenesis and spermatogenic disorders, as well as fertility preservation.

**2. What are the purpose and contents of the research?**

We intent to conduct the following researches: to explore the feasibility and efficiency of different fertility preservation methods for cancer patients and compare their fertility outcomes; to make standardized and individualized programs of fertility recovery for infertile patients based on their characteristics (ages, causes, medical history, etc); to explore novel mechanisms of human normal testicle development, germ cell development, spermatogenesis, and spermatogenic disorders, based on multi-omics study of testicular tissues from patients with normal or impaired spermatogenesis.

**3. What you need to do if choose to participate in the research?**

You need to provide all relevant medical history of yourself or your children in detail to help us make the best therapy choice. You should be aware that many diseases that impair testicle development and spermatogenesis could only be treated or cured by surgery, like most testicular tumours, cryptorchidism, testicular torsion, etc. During the surgery, there is possibility that the whole or a part of impaired testicle will be excised for further pathological examination according to the clinical practice guidelines of China Urology Association, and only the remaining testicular tissues are collected for the research.

**4. What will you benefit from this research?**

In addition to pathological examination, we will re-evaluate the testicular tissue in detail, like the extent of spermatogenesis impairment and testicle degeneration, and provide free long-term instructions and consultation to you or your children about fertility recovery and testicular development.

**5. Will there be risks or discomforts for you?**

In this research, we will not arrange any additional examination or therapy for you or your children, and the testicular tissues for research will only be collected after all procedures including pathological examination, which otherwise will be disposed by hospital if you refuse to authorize. Also, the personal information of yourself or your children will be protected tightly. Therefore, there is no risks or discomforts for you or your children.

**6. Whether you can drop out during the research?**

It is your right to choose when to participate in or drop out the research. All your choice will not affect the relations between you and the doctors, as well as the normal medical procedure.

**7. Will your personal information be revealed in this research?**

In this research, all your personal information (like name, medical history, treatment, etc) will be completely and safely saved in the hospital. Nobody except for your doctors, investigators, and ethical committee members will be accessible to your information. Any results of the research, including all types of publications, will not reveal your information.

**8. Who you can contact when encounter any problems?**

You can contact any of your doctor or investigator to ask any question about this research at any time, and timely and detailed reply is guaranteed. Please feel free to tell us your questions and confusion.

**9. Others**

It is your right to choose whether to participate in the research or not. Before making decisions, please consult your doctors in detail.

Thank you for reading the above statement. If you choose to participate in the research, please tell your doctor, and he/she will arrange anything for you.

**Doctor (Investigator):**

**Telephone:**

**Date:**

**Agreement announcement:**

I have carefully read the above statement about the research, and discussed my questions in detail with my doctors and investigators. All my questions have been well addressed.

I have been informed of the benefits and risks of participating in the research. I have enough time to think over and make my decision to participate in the research. I am aware that I have the right contact any of my doctor or investigator to ask any question about this research at any time, and I am free to drop out the research, which will not affect the relations between me and the doctors, as well as the normal medical procedure.

I decide to participate in the research and cooperate with my doctors and investigators. I will get a copy of the informed consent with doctor’s signature and date.

**Patient’s signature: Admission number:**

**Legal guardian of the patient (signature): Relationship to the patient:**

**Date:**

**Informed consent for surgery in Chinese (5-years-old child)**

**
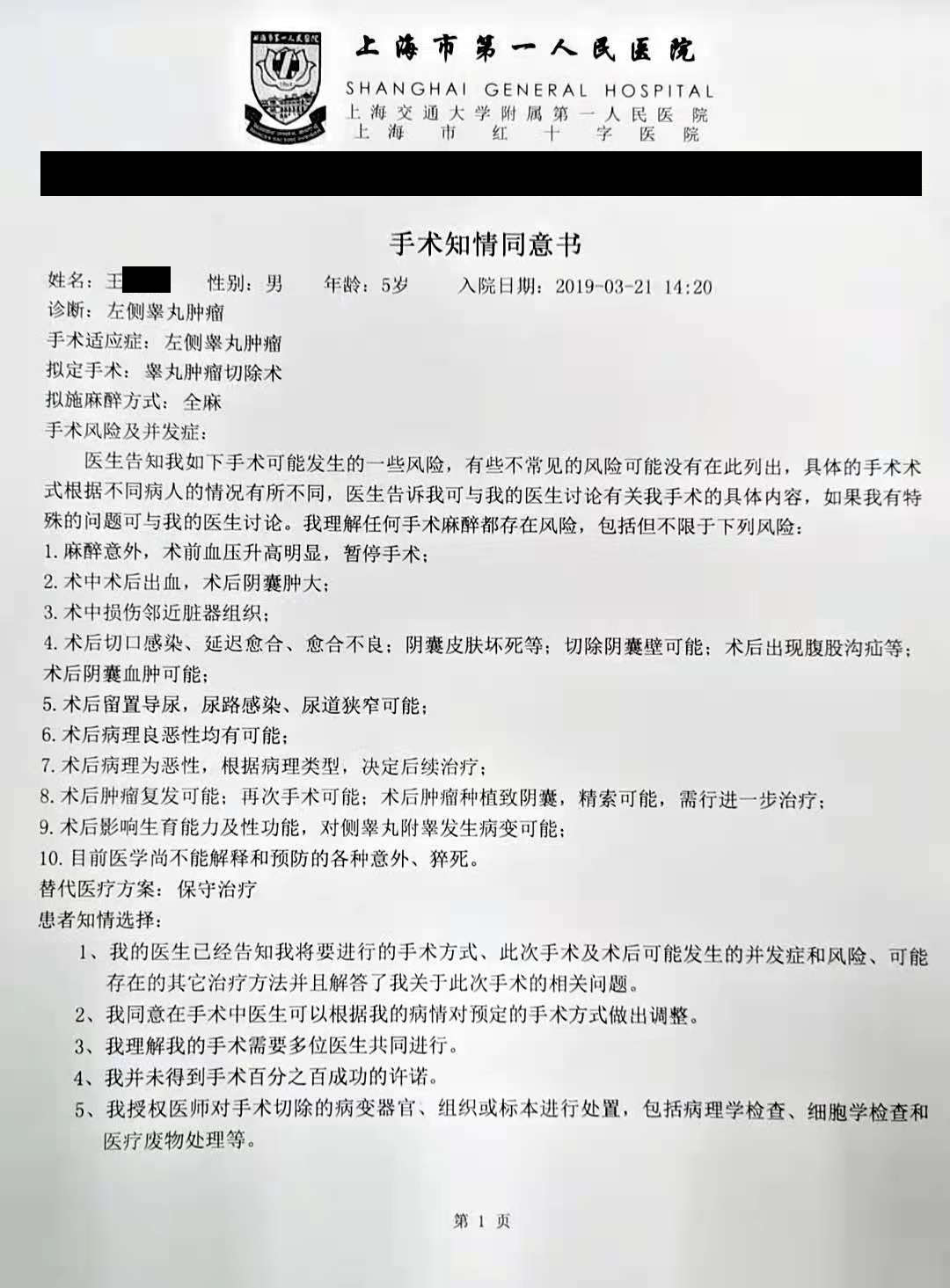

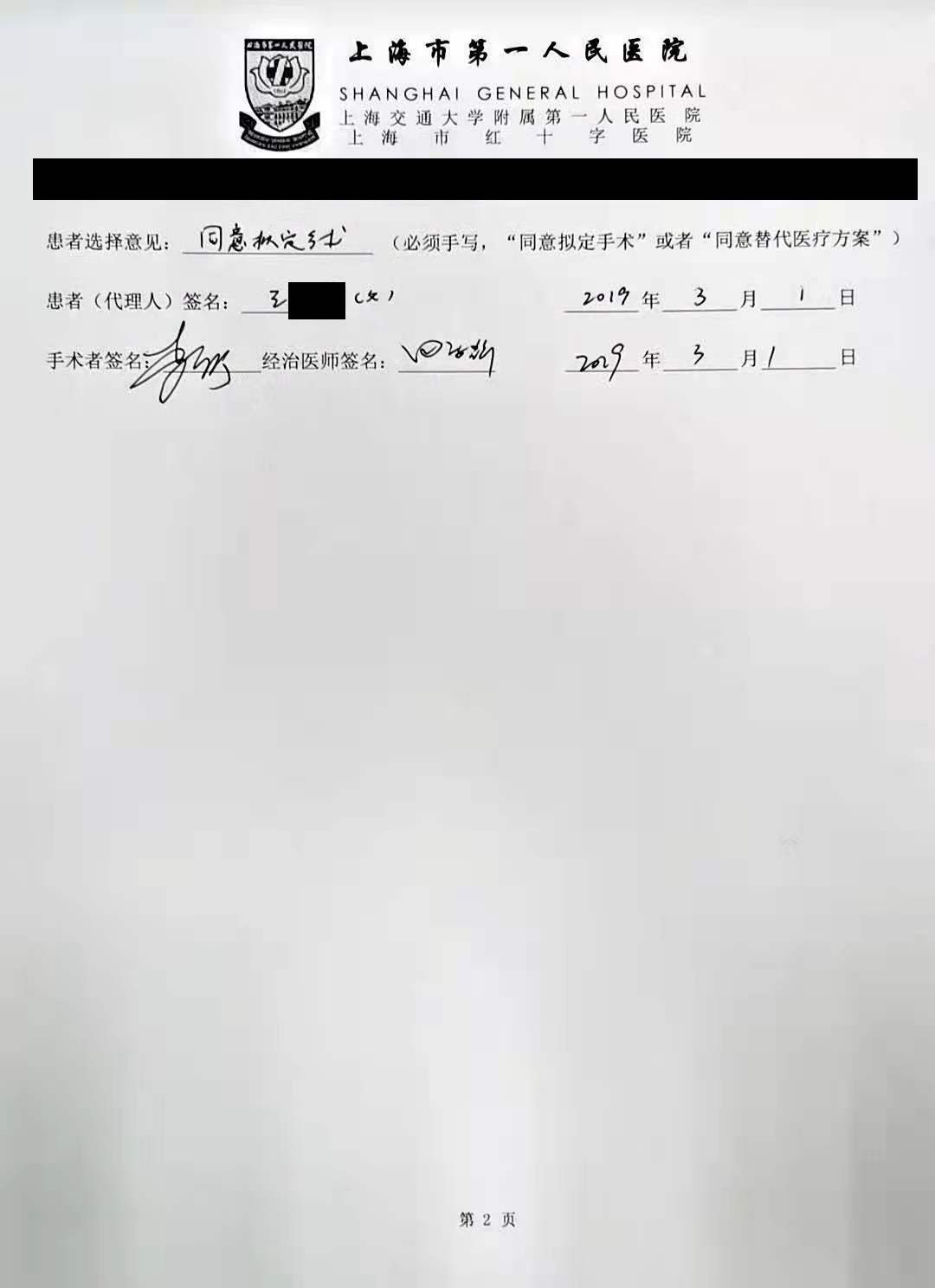
**

**Informed consent for surgery in English (5-years-old child)**

**Name:** **Gender:** male **Age:** 5 years old

**Date of Admission:** 2019-03-21 14:20

**Diagnosis:** Left testicular tumor

**Surgical indication****:** Left testicular tumor

**Proposed surgery:** Testicular tumor resection

**Method of anesthesia:** General anesthesia

**Surgical risks and complications:**

The doctor informed me of the possible risks of the following surgery. Some uncommon risks may not be listed here. The specific surgical procedures vary according to the conditions of different patients. The doctor told me that I can discuss with my doctor about my surgery if I have specific problems. I understand that any surgical anesthesia has risks, including but not limited to the following risks:

1. Anesthesia accident; cardiovascular and cerebrovascular accident, which may be life-threatening in serious cases; the blood pressure increased significantly before operation and the operation may be suspended;

2. Intraoperative and postoperative bleeding; postoperative scrotal enlargement;

3. Injury to adjacent organs and tissues during operation;

4. Postoperative incision infection; delayed healing; poor healing; scrotal skin necrosis, etc.; removal of the scrotal wall may be possible; postoperative inguinal hernia, etc.; postoperative scrotal hematoma;

5. Postoperative indwelling catheterization; urinary tract infection; urethral stricture;

6. The tumour may be benign or malignant according to pathological examination;

7. If the tumour is malignant, follow-up treatment is determined according to the pathological type;

8. Postoperative tumor recurrence; reoperation; postoperative tumor implantation to the scrotum, spermatic cord may occur and further treatment is required;

9. Postoperative fertility and sexual function may be affected; the contralateral testis and epididymis may have lesions;

10. All kinds of accidents and sudden deaths that currently cannot be explained and prevented by medicine;

**Alternative medical treatment:** conservative treatment;

**Patient’s choice:**

1. My doctor has informed me of the operation to be performed, the possible complications and risks of this operation, other possible treatment methods, and has answered my questions about the operation.

2. I agree that the doctor can adjust the scheduled operation method according to my condition during the operation.

3. I understand that my surgery needs to be performed by multiple doctors.

4. I am not promised of certain success of the operation.

5. I authorize doctors to dispose the impaired organs, tissues or specimens removed by surgery, including pathological examinations, cytological examinations, and medical waste disposal.

**Patient’s choice:**  (Must be handwritten, "Agree to the proposed surgery" or "Agree to an alternative medical plan)

**Patient (agent) signature: Date:**

**Signature of surgeon: Signature of attending doctor: Date:**

**Informed consent for research in Chinese (5-years-old child)**

**
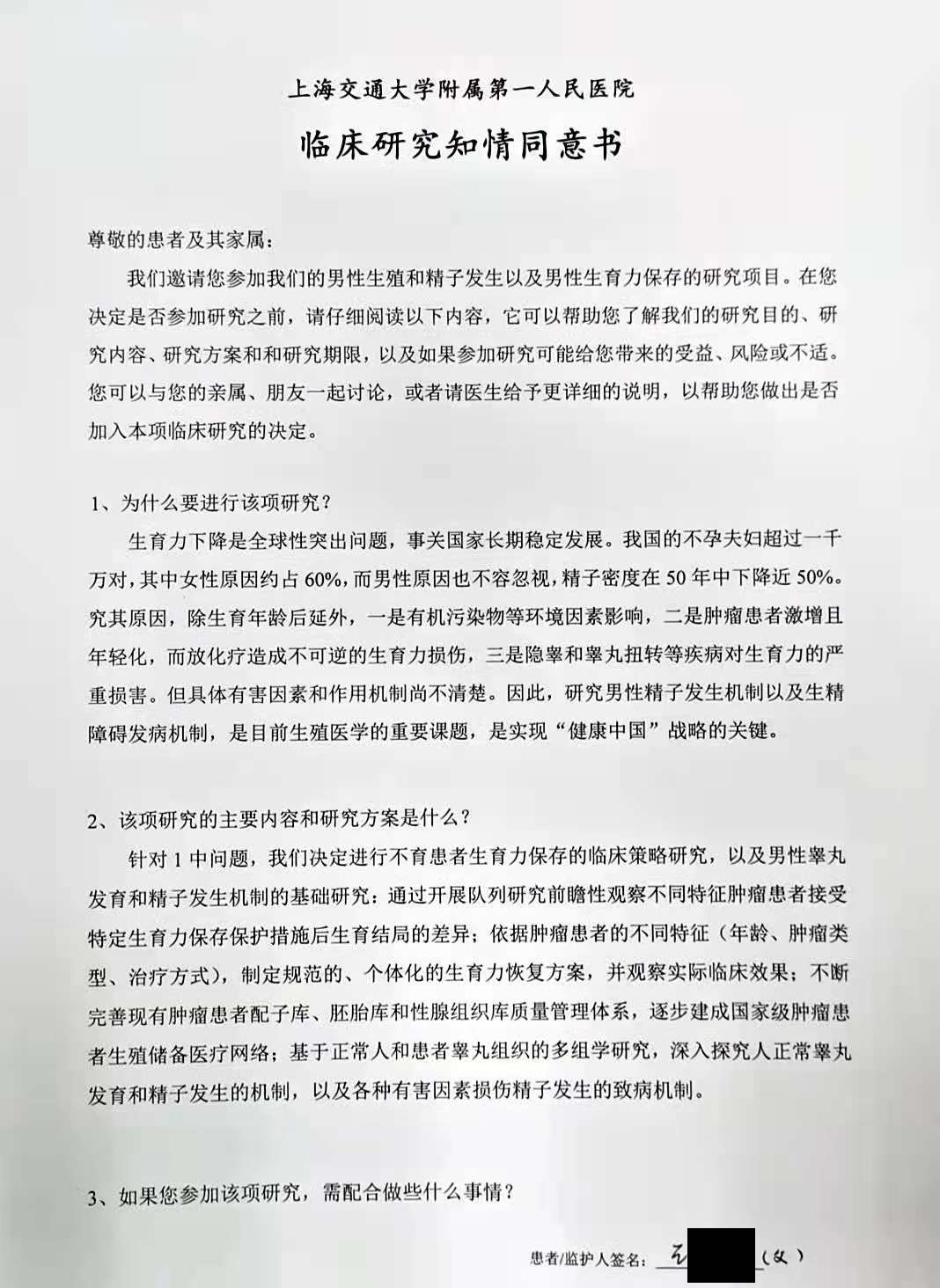

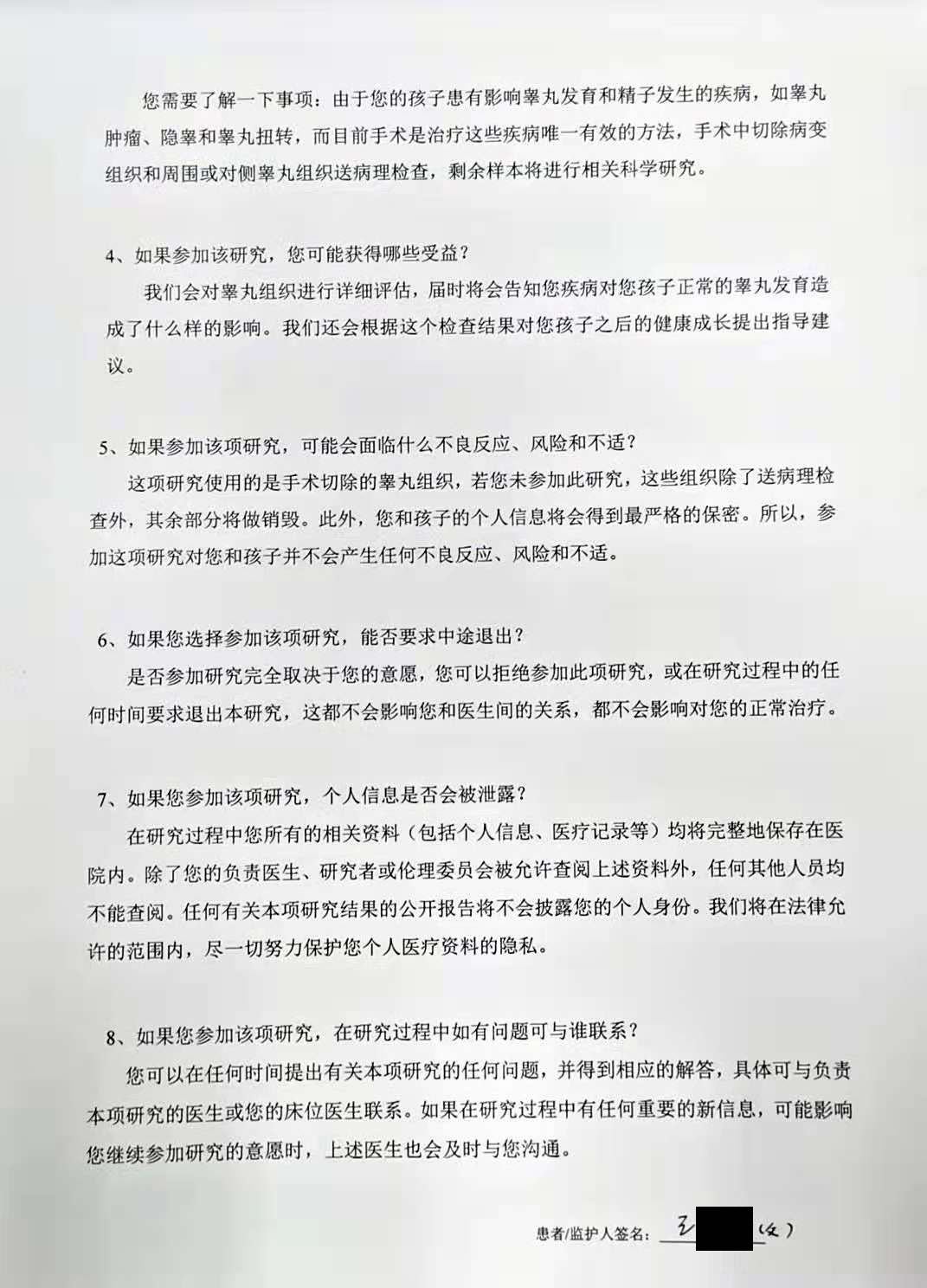

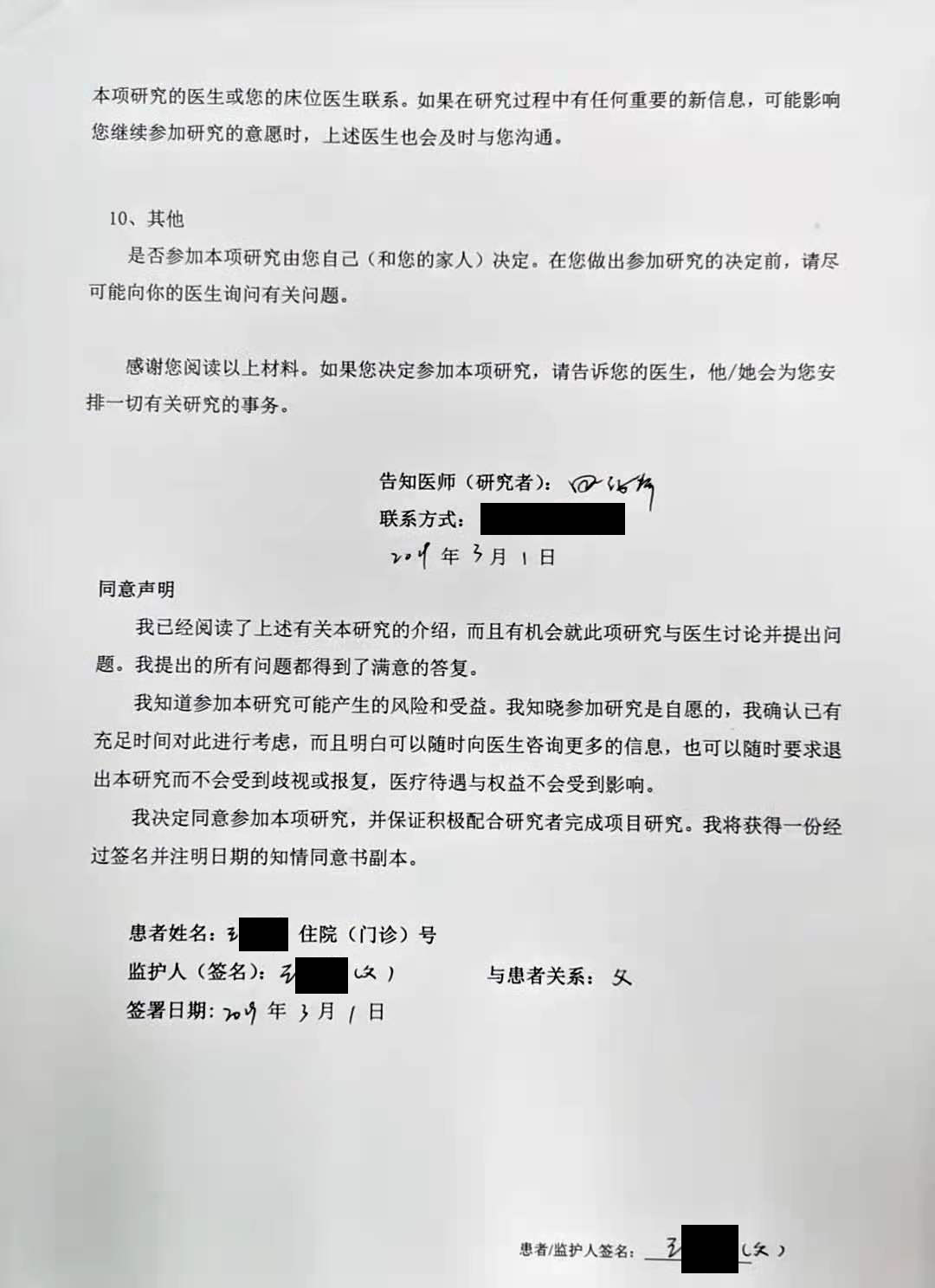
**

**Informed consent for research in English (5-years-old child)**

The same with the informed consent for 2-years-old child written above.

**Informed consent for surgery in Chinese (11-years-old child)**

**
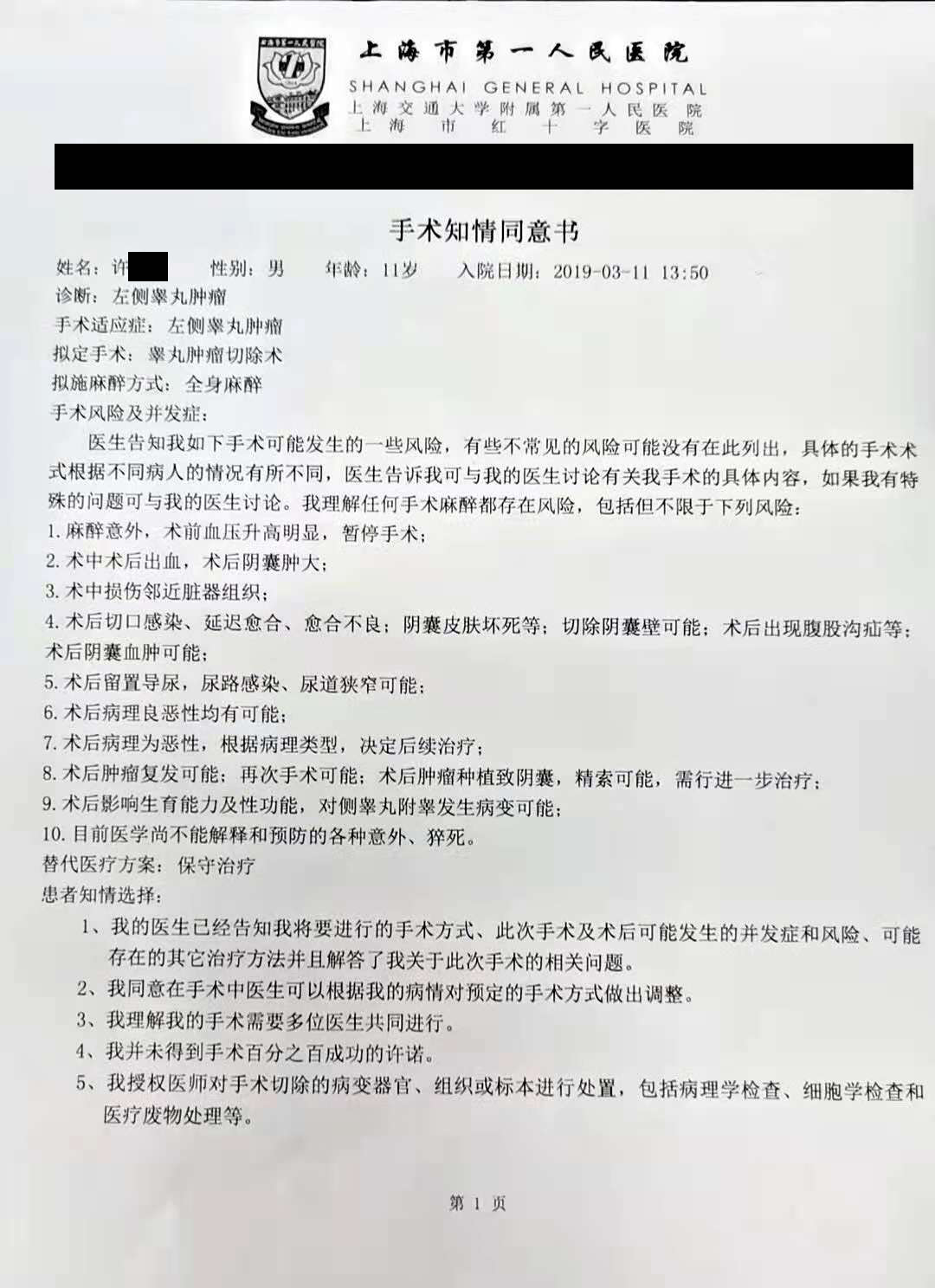

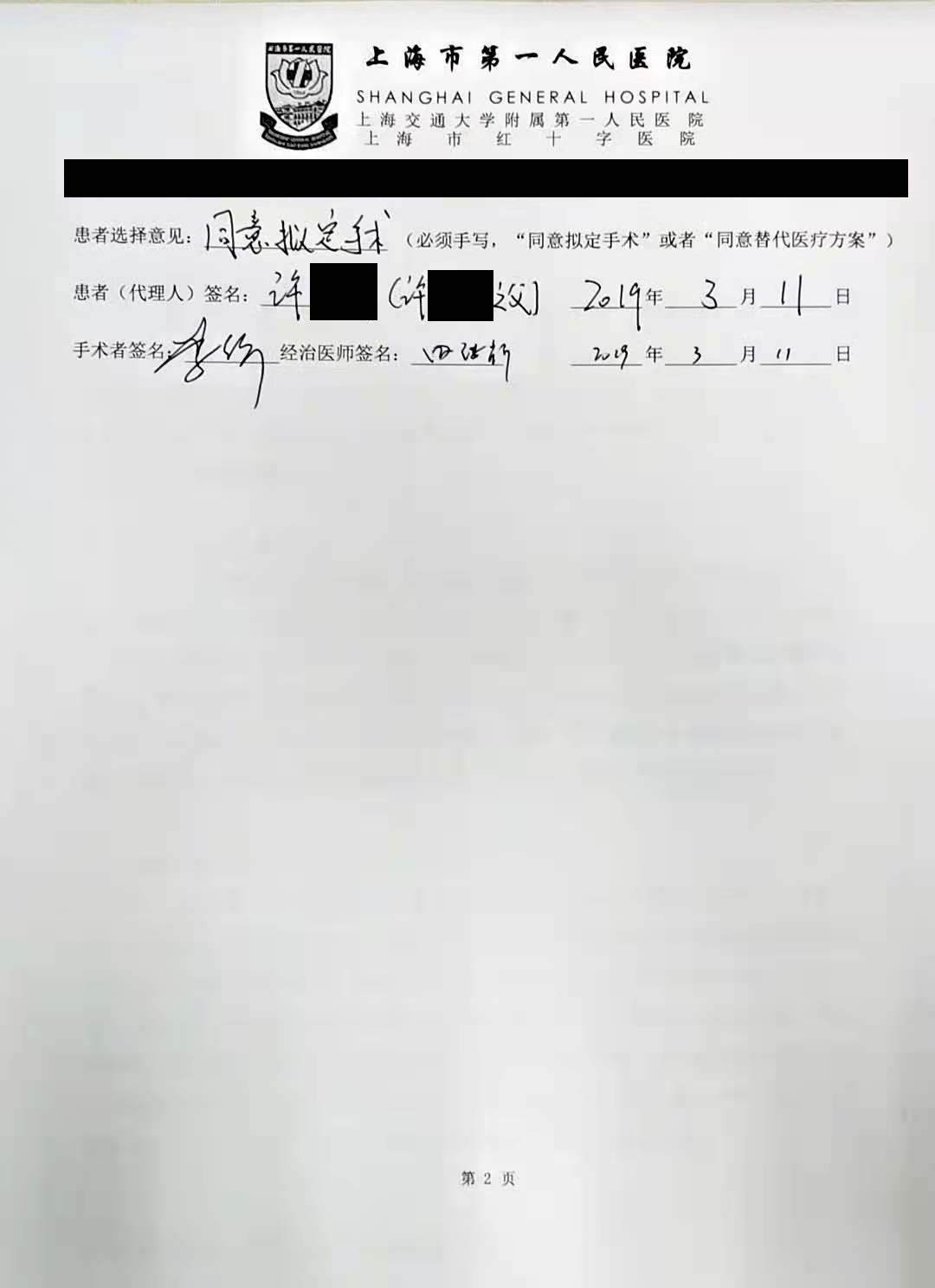
**

**Informed consent for surgery in English (11-years-old child)**

**Name:** **Gender:** male **Age:** 11 years old

**Date of Admission:** 2019-03-11 13:50

**Diagnosis:** Left testicular tumor

**Surgical indication:** Left testicular tumor

**Proposed surgery:** Testicular tumor resection

**Method of anesthesia:** General anesthesia

**Surgical risks and complications:**

The doctor informed me of the possible risks of the following surgery. Some uncommon risks may not be listed here. The specific surgical procedures vary according to the conditions of different patients. The doctor told me that I can discuss with my doctor about my surgery if I have specific problems. I understand that any surgical anesthesia has risks, including but not limited to the following risks:

1. Anesthesia accident; cardiovascular and cerebrovascular accident, which may be life-threatening in serious cases; the blood pressure increased significantly before operation and the operation may be suspended;

2. Intraoperative and postoperative bleeding; postoperative scrotal enlargement;

3. Injury to adjacent organs and tissues during operation;

4. Postoperative incision infection; delayed healing; poor healing; scrotal skin necrosis, etc.; removal of the scrotal wall may be possible; postoperative inguinal hernia, etc.; postoperative scrotal hematoma;

5. Postoperative indwelling catheterization; urinary tract infection; urethral stricture;

6. The tumour may be benign or malignant according to pathological examination;

7. If the tumour is malignant, follow-up treatment is determined according to the pathological type;

8. Postoperative tumor recurrence; reoperation; postoperative tumor implantation to the scrotum, spermatic cord may occur and further treatment is required;

9. Postoperative fertility and sexual function may be affected; the contralateral testis and epididymis may have lesions;

10. All kinds of accidents and sudden deaths that currently cannot be explained and prevented by medicine;

**Alternative medical treatment:** conservative treatment;

**Patient’s choice:**

1. My doctor has informed me of the operation to be performed, the possible complications and risks of this operation, other possible treatment methods, and has answered my questions about the operation.

2. I agree that the doctor can adjust the scheduled operation method according to my condition during the operation.

3. I understand that my surgery needs to be performed by multiple doctors.

4. I am not promised of certain success of the operation.

5. I authorize doctors to dispose the impaired organs, tissues or specimens removed by surgery, including pathological examinations, cytological examinations, and medical waste disposal.

**Patient’s choice:**  (Must be handwritten, "Agree to the proposed surgery" or "Agree to an alternative medical plan)

**Patient (agent) signature: Date:**

**Signature of surgeon: Signature of attending doctor: Date:**

**Informed consent for research in Chinese (11-years-old child)**


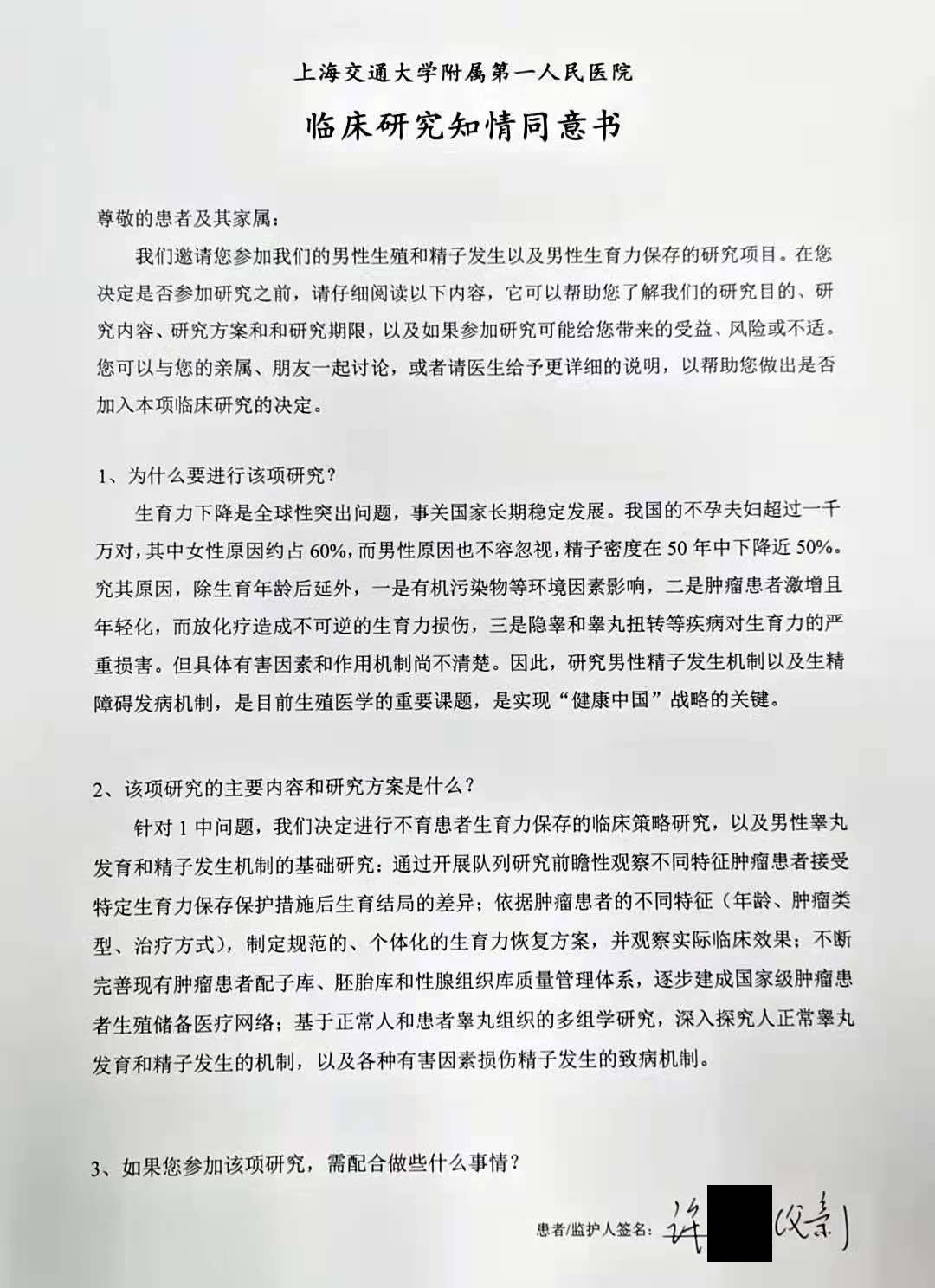

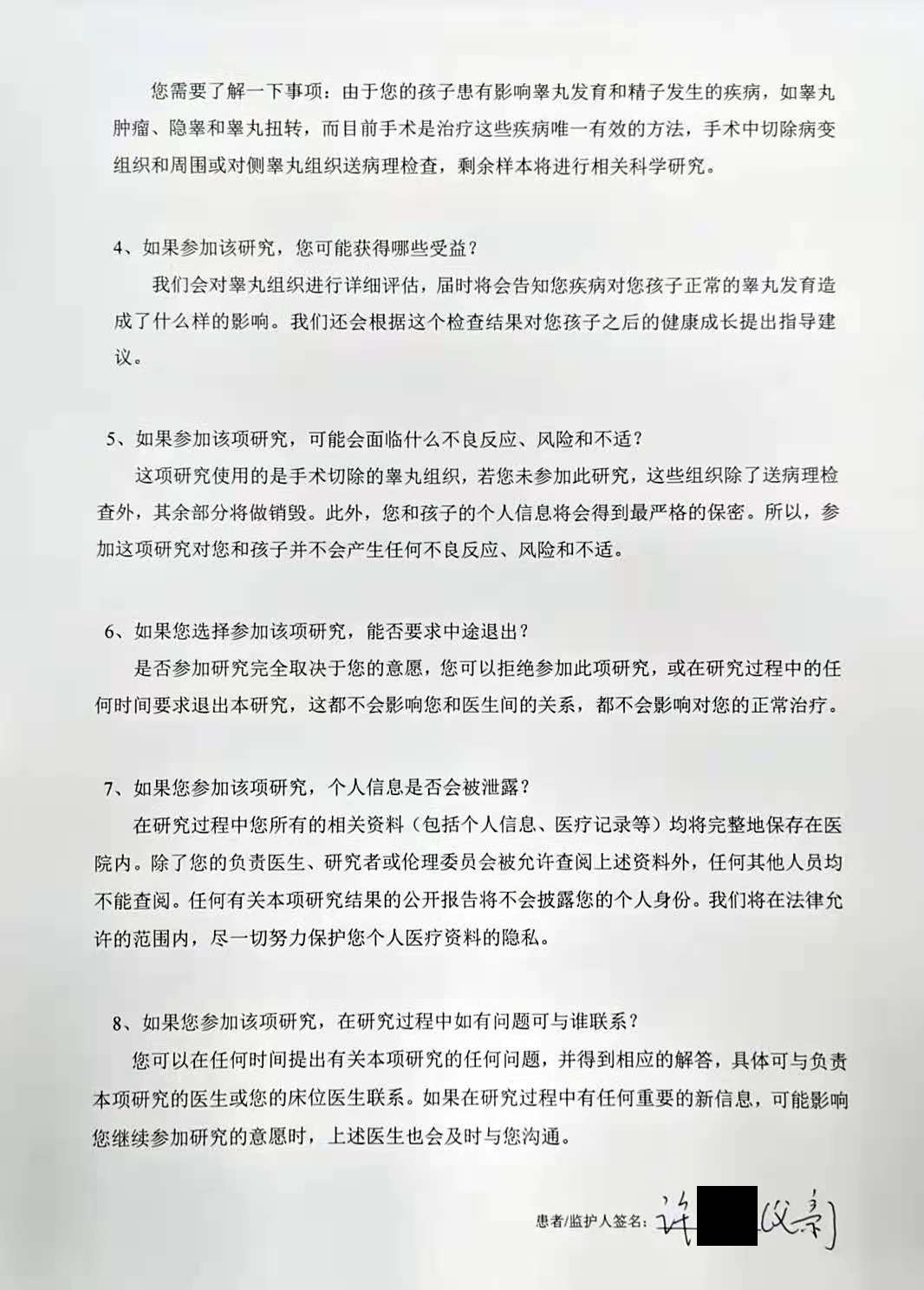

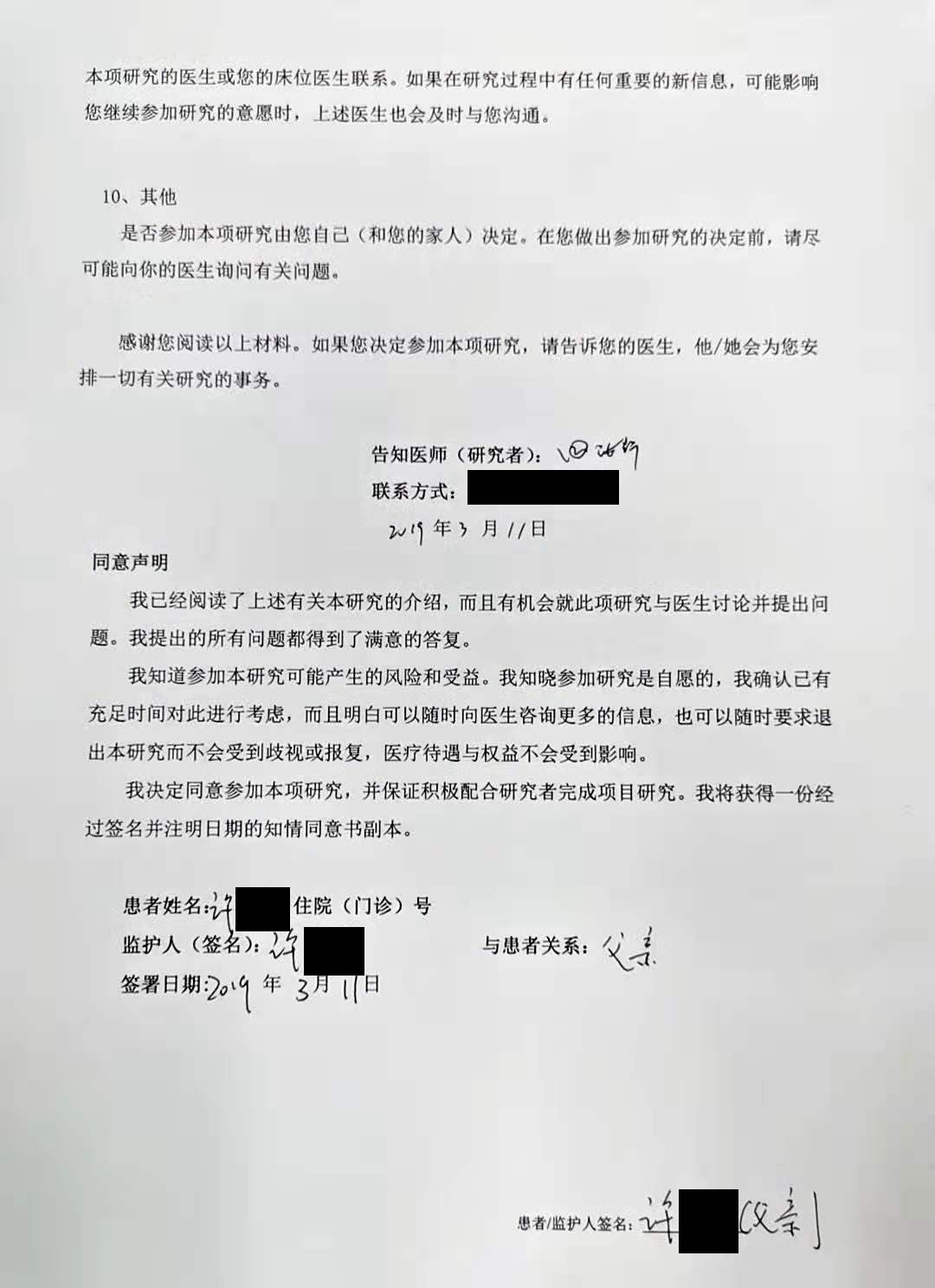


**Informed consent for research in English (11-years-old child)**

The same with the informed consent for 2-years-old child written above.

**Informed consent for surgery in Chinese (17-years-old child)**

**
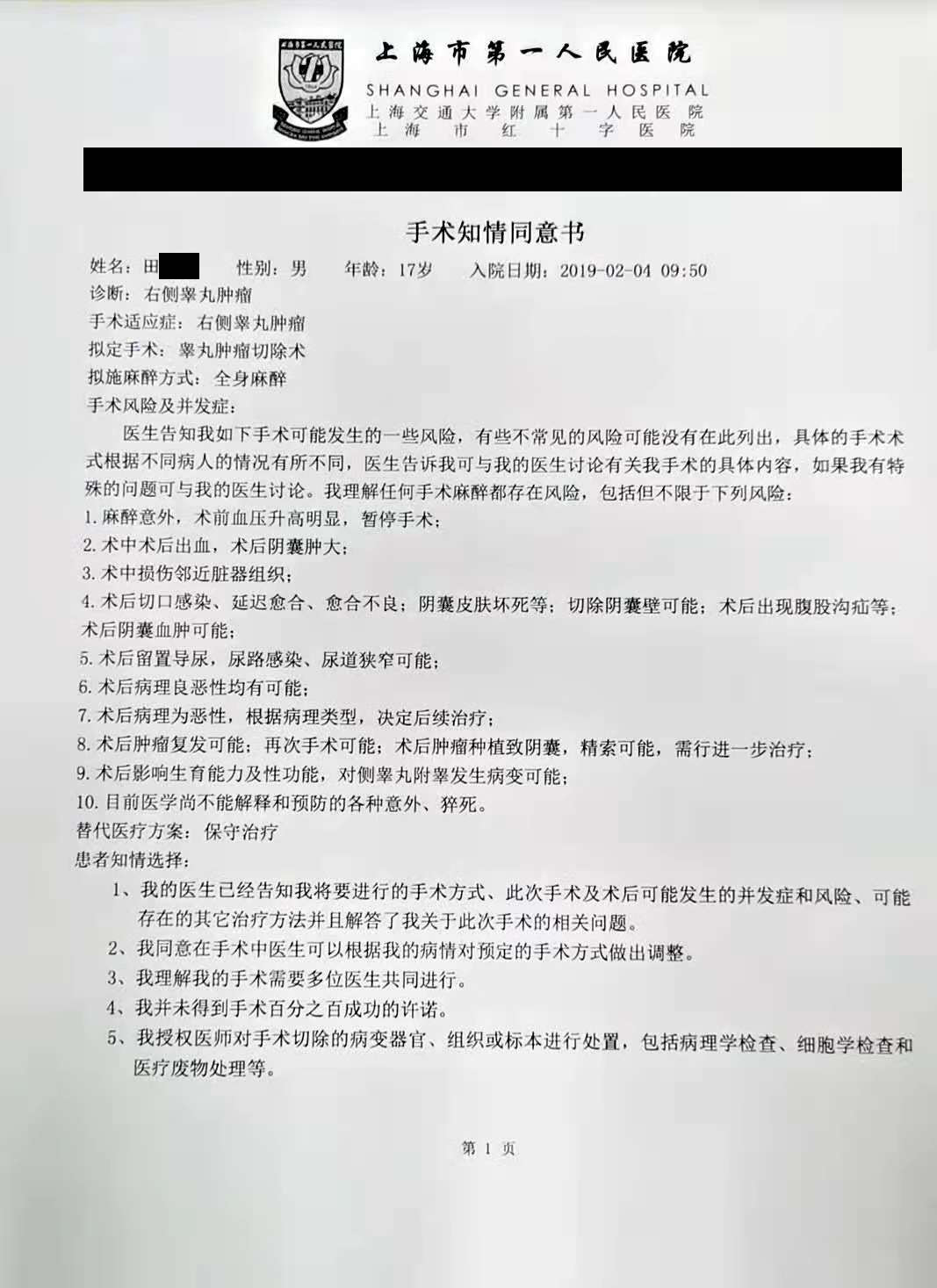

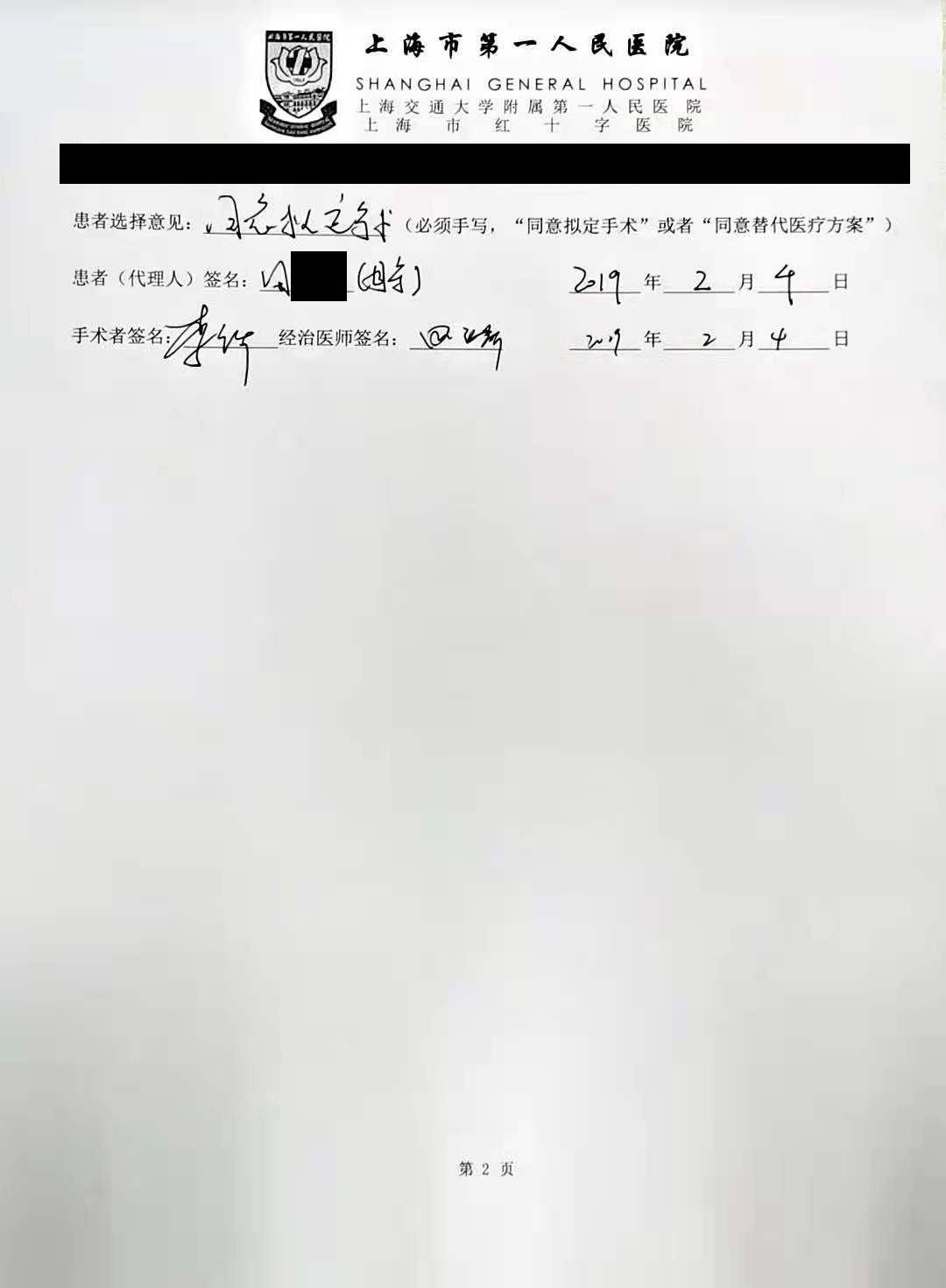
**

**Informed consent for surgery in English (17-years-old child)**

**Name:** **Gender:** male **Age:** 17 years old

**Date of Admission:** 2019-02-04 09:50

**Diagnosis:** Right testicular tumor

**Surgical indication:** Right testicular tumor

**Proposed surgery:** Testicular tumor resection

**Method of anesthesia:** General anesthesia

**Surgical risks and complications:**

The doctor informed me of the possible risks of the following surgery. Some uncommon risks may not be listed here. The specific surgical procedures vary according to the conditions of different patients. The doctor told me that I can discuss with my doctor about my surgery if I have specific problems. I understand that any surgical anesthesia has risks, including but not limited to the following risks:

1. Anesthesia accident; cardiovascular and cerebrovascular accident, which may be life-threatening in serious cases; the blood pressure increased significantly before operation and the operation may be suspended;

2. Intraoperative and postoperative bleeding; postoperative scrotal enlargement;

3. Injury to adjacent organs and tissues during operation;

4. Postoperative incision infection; delayed healing; poor healing; scrotal skin necrosis, etc.; removal of the scrotal wall may be possible; postoperative inguinal hernia, etc.; postoperative scrotal hematoma;

5. Postoperative indwelling catheterization; urinary tract infection; urethral stricture;

6. The tumour may be benign or malignant according to pathological examination;

7. If the tumour is malignant, follow-up treatment is determined according to the pathological type;

8. Postoperative tumor recurrence; reoperation; postoperative tumor implantation to the scrotum, spermatic cord may occur and further treatment is required;

9. Postoperative fertility and sexual function may be affected; the contralateral testis and epididymis may have lesions;

10. All kinds of accidents and sudden deaths that currently cannot be explained and prevented by medicine;

**Alternative medical treatment:** conservative treatment;

**Patient’s choice:**

1. My doctor has informed me of the operation to be performed, the possible complications and risks of this operation, other possible treatment methods, and has answered my questions about the operation.

2. I agree that the doctor can adjust the scheduled operation method according to my condition during the operation.

3. I understand that my surgery needs to be performed by multiple doctors.

4. I am not promised of certain success of the operation.

5. I authorize doctors to dispose the impaired organs, tissues or specimens removed by surgery, including pathological examinations, cytological examinations, and medical waste disposal.

**Patient’s choice:**  (Must be handwritten, "Agree to the proposed surgery" or "Agree to an alternative medical plan)

**Patient (agent) signature: Date:**

**Signature of surgeon: Signature of attending doctor: Date:**

**Informed consent for research in Chinese (17-years-old child)**


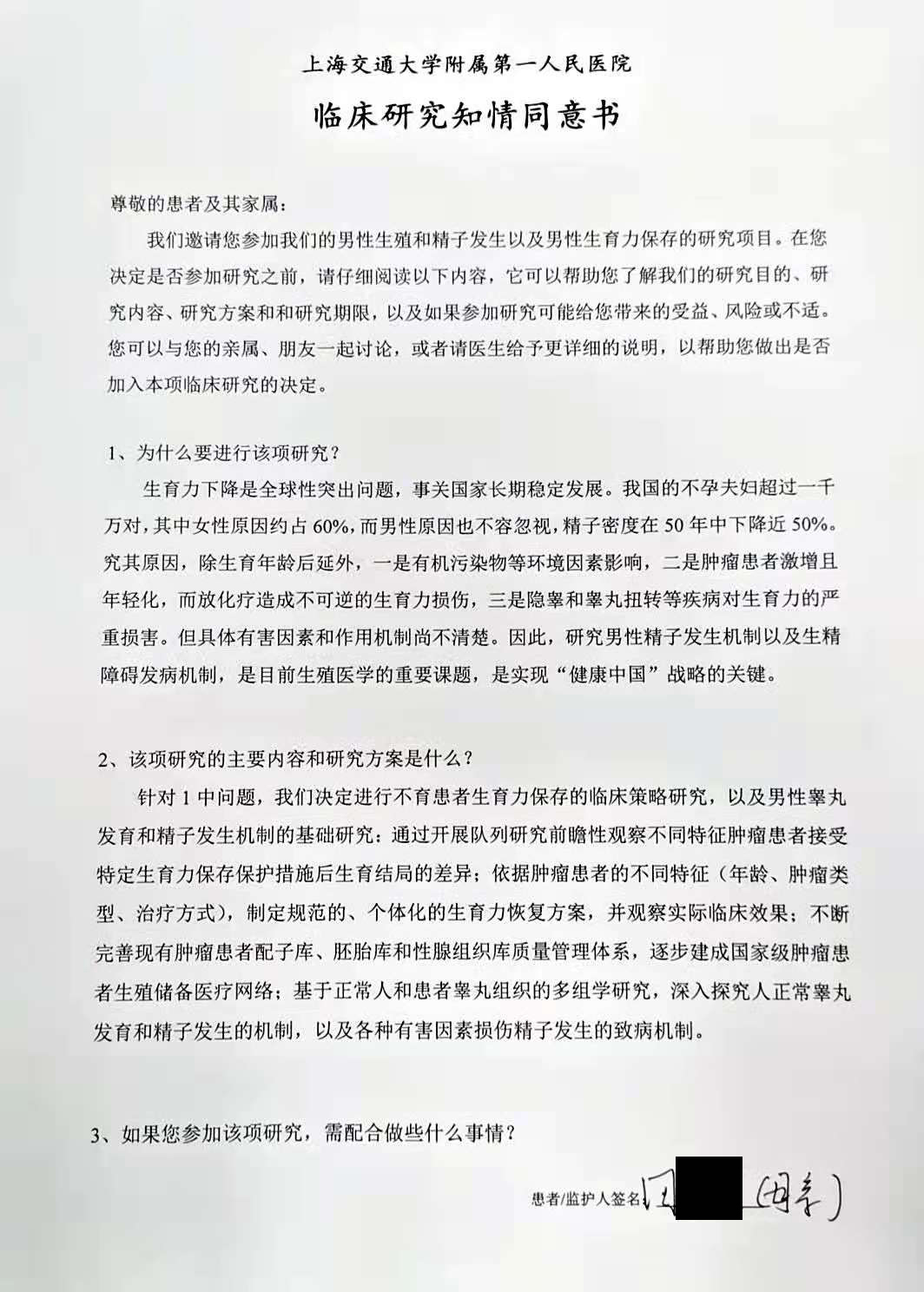

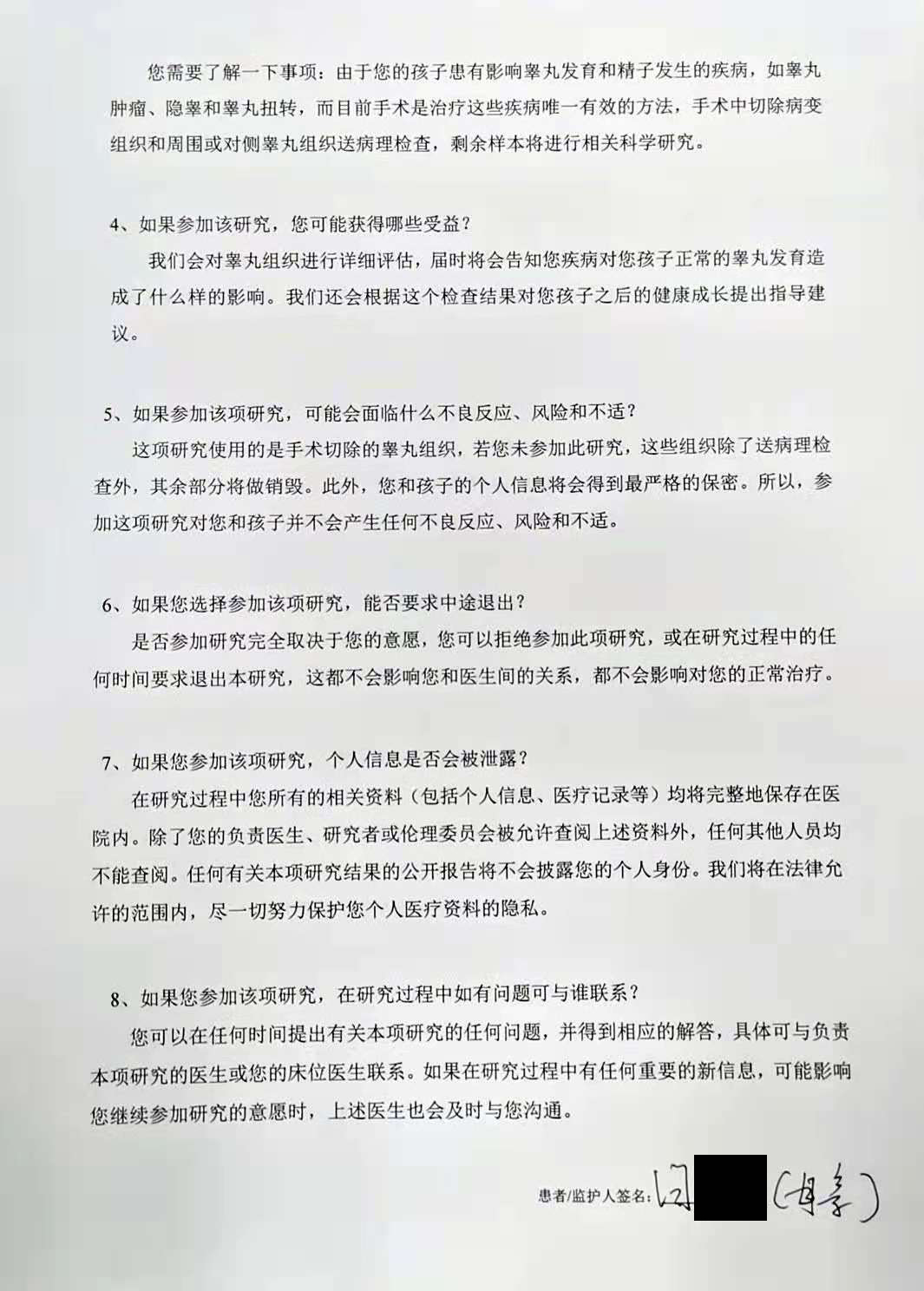

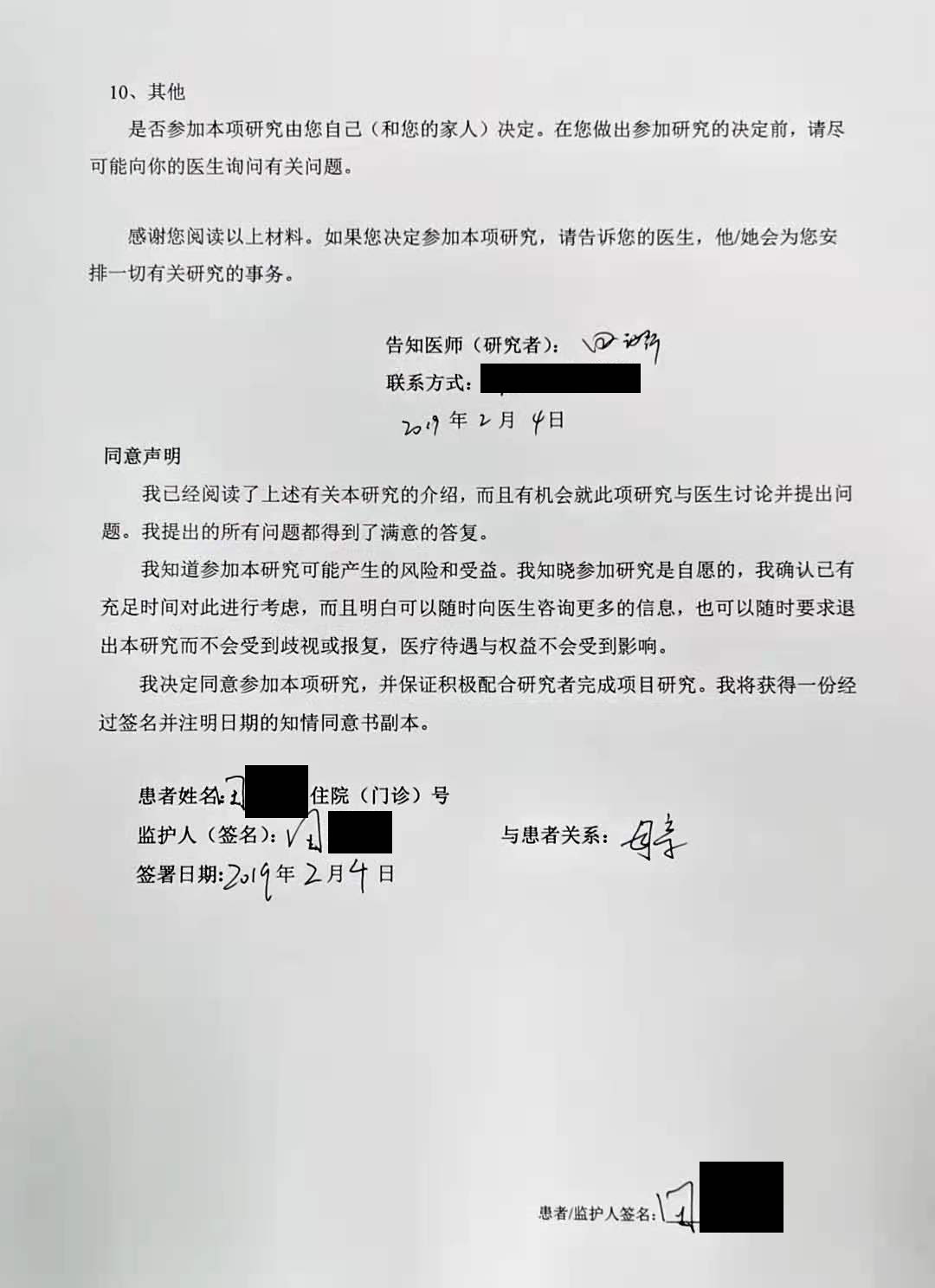


**Informed consent for research in English (17-years-old child)**

The same with the informed consent for 2-years-old child written above.
